# Electrochemical Enantioselective C–H Annulation by Achiral Rhodium(III)/Chiral Brønsted Base Domino Catalysis

**DOI:** 10.1021/acscatal.4c01886

**Published:** 2024-05-10

**Authors:** Yanjun Li, Jiawei Xu, João C. A. Oliveira, Alexej Scheremetjew, Lutz Ackermann

**Affiliations:** † Institut für Organische und Biomolekulare Chemie, Georg-August-Universität Göttingen, Tammannstraße 2, 37077 Göttingen, Germany

**Keywords:** electrocatalysis, enantioselective C−H annulation, rhodium(III), chiral Brønsted base, domino catalysis

## Abstract

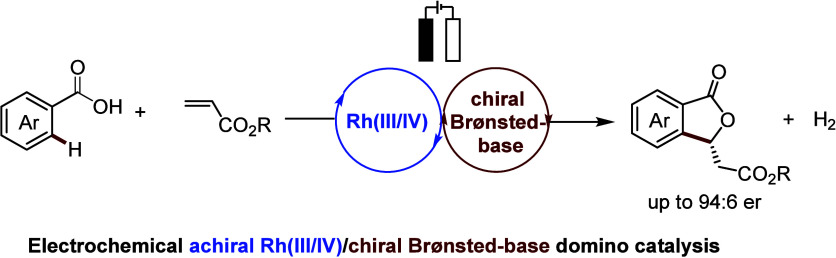

Rhodium(III)-catalyzed enantioselective C–H activation has emerged as a powerful tool for assembling enabling chiral molecules. However, this approach is significantly hampered by the cumbersome synthetic routes for preparing chiral rhodium catalysts. In sharp contrast, we herein report on an electrochemical domino catalysis system that exploits an achiral Cp*-rhodium catalyst along with an easily accessible chiral Brønsted base for an enantioselective C–H activation/annulation reaction of alkenes by benzoic acids. Our strategy offers an environmentally benign and most user-friendly approach for assembling synthetically useful chiral phthalides in good enantioselectivity, employing electricity as the sustainable oxidant.

## Introduction

**Scheme 1:**
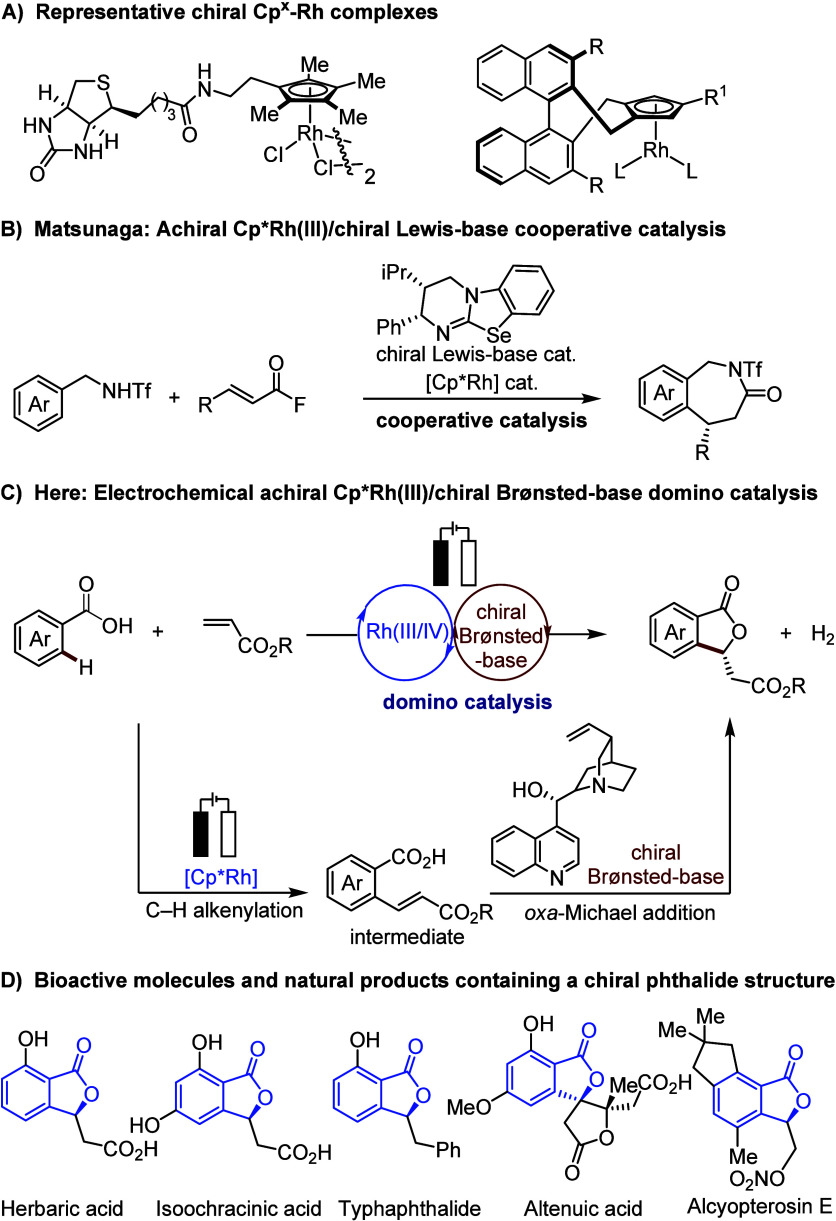
(A) Representative Chiral Cp^x^-Rh Complexes; (B,C) Enantioselective C–H Annulation Using Achiral Cp^x^-Rhodium Catalysts Combined with Chiral Organocatalysts; (D) Related Bioactive Molecules and Natural Products Containing a Chiral Phthalide Structure

Rhodium-catalyzed C–H activation has emerged as an increasingly powerful platform for constructing C–C and C–Het bonds.^1−4^ Rhodium(III) complexes featuring a pentamethylcyclopentadienyl (Cp*) or related cyclopentadienyl-type (Cp^x^) ligand have proven to be particularly effective catalysts for C–H activation reactions due to their robustness, outstanding reactivity, and high selectivity.^1,2^ Pioneering studies on rhodium(III)-catalyzed asymmetric C–H activation reactions were achieved using chiral Cp^x^ ligand environments by Ward and Rovis,^5,6^ as well as Cramer.^7^ Subsequently, various chiral Cp^x^-rhodium complexes have been elegantly devised by Cramer,^8−11^ You,^12,13^ Antonchick/Waldmann,^14^ Perekalin,^15^ Blakey,^16^ and Wang,^17,18^ among others.^19−23^ These well-designed chiral Cp^x^-rhodium complexes (Scheme 1A) enabled a series of significant enantioselective C–H functionalization reactions.^5−29^ However, the practicality of these methods was hindered by the relatively cumbersome synthetic routes toward these chiral Cp^x^ ligands. Thus, alternative approaches for rhodium(III)-catalyzed enantioselective C–H activation have gained considerable attention, namely using achiral Cp^x^-rhodium catalyst combined with easily available chiral additives, such as chiral anions^30^ carboxylic acids,^31−35^ a chiral transient directing group,^36^ or a chiral Lewis-base.^37^ For instance, Matsunaga elegantly reported a Cp*-rhodium(III) complex with a chiral disulfonate anion, which enabled enantioselective C–H alkylation reactions.^30^ Subsequently, the same group revealed a [Cp*RhCl_2_]_2_/chiral carboxylic acid-catalyzed enantioselective C–H functionalization of diarylmethanamines.^35^ Wang applied a transient directing group strategy to realize rhodium(III)-catalyzed enantioselective dimerization of aldehydes.^36^ Recently, an achiral Cp*-rhodium(III)/chiral Lewis base isochalcogenureas (ICU) cooperative catalysis for enantioselective C–H activation/[4 + 3] annulation was developed by Matsunaga (Scheme 1B).^37^ Despite these indisputable advances, the use of achiral Cp^x^-rhodium complexes in combination with external chiral sources to achieve enantioselective C–H activation continues to be scarce. Therefore, the pursuit of novel cooperative catalytic systems in rhodium-catalyzed enantioselective C–H functionalization is highly sought-after, albeit particularly challenging.

Organic electrosynthesis considerably improves the sustainability of molecular synthesis by utilizing electricity as an eco-friendly alternative to stoichiometric amounts of redox reagents, with the potential to contribute to a green hydrogen economy.^38−44^ Electrochemical transition-metal-catalyzed enantioselective C–H functionalization has been identified as a versatile platform for the construction of chiral molecules.^45−58^ These electrochemical pathways commonly rely on the interaction of a unique chiral catalyst with the substrates.^45−55^ In sharp contrast, electrochemical cooperative catalysis,^56−59^ namely two catalysts working in concert through two distinct catalytic cycles, continues to be underdeveloped.

Within our continuous interest in metallaelectrocatalyzed C–H activation,^60−66^ we questioned whether it would indeed be possible to introduce a domino catalytic system enabled by cooperative rhodium(III) catalysis and asymmetric Brønsted base catalysis^67^ (Scheme 1C). Thus, an alkenylated intermediate would be generated via electrooxidative rhodium-catalyzed C–H olefination and would then participate in a chiral Brønsted base-catalyzed enantioselective *oxa*-Michael addition reaction, generating the chiral phthalide product. As a consequence of our efforts, we have now identified the electrochemical domino catalysis for enantioselective C–H annulation (Scheme 1C), which we report herein. Notable features of our findings include: (a) commercially available Cp*-rhodium and cinchonine were used as catalysts, avoiding the use of chiral Cp^x^-rhodium catalysts prepared in lengthy steps, (b) the first electrochemical domino catalysis for enantioselective C–H functionalization, and (c) the use of bulk commodity chemicals as starting materials to produce chiral phthalides, which are important structural motifs found in biologically active compounds and natural products^68^ (Scheme 1D).

## Results and Discussion

Our studies were initiated by probing various reaction conditions for the envisioned electrochemical achiral Cp*-rhodium/chiral Brønsted base domino catalysis. Benzoic acid **1a** and alkene **2a** were chosen as model substrates, and different chiral Brønsted base catalysts were initially probed under the reaction with [Cp*RhCl_2_]_2_ as the catalyst, NaOPiv as the additive, *n*Bu_4_NPF_6_ as the electrolyte, and DCE as the solvent (Table 1; see also the Supporting Information, Table S1). Representative chiral Brønsted bases, such as chiral thiourea **B1**, chiral amine **B2**, and cinchona derivative catalyst **B3**, proved to be ineffective, resulting in only trace amounts of product formation (entries 1–3). Inspired by the work of ruthenium/cinchonine catalytic system,^69^ we tested the readily available and inexpensive cinchonine **B4** (entry 4). Encouragingly, product **3aa** was obtained in 85% yield with an 86:14 enantiomeric ratio (er). Further investigation of cinchona derivative catalysts (**B5**–**B7**) revealed that the enantioselectivity of the product was not improved (entries 5–7). Then, we explored the effect of other conditions (entries 8–10). The er value of **3aa** could be further improved to 92:8 when we used Cp*Rh(OAc)_2_ as the catalyst, *n*Bu_4_NBARF as the electrolyte, and CPME as the solvent (entry 10). Increasing the constant current to 1 mA resulted in a relatively lower yield (entry 11). Control experiments indicated that Cp*Rh(OAc)_2_ (entry 12), chiral Brønsted base (entry 13), and electricity (entry 14) were essential to the reaction.

**Table 1:**
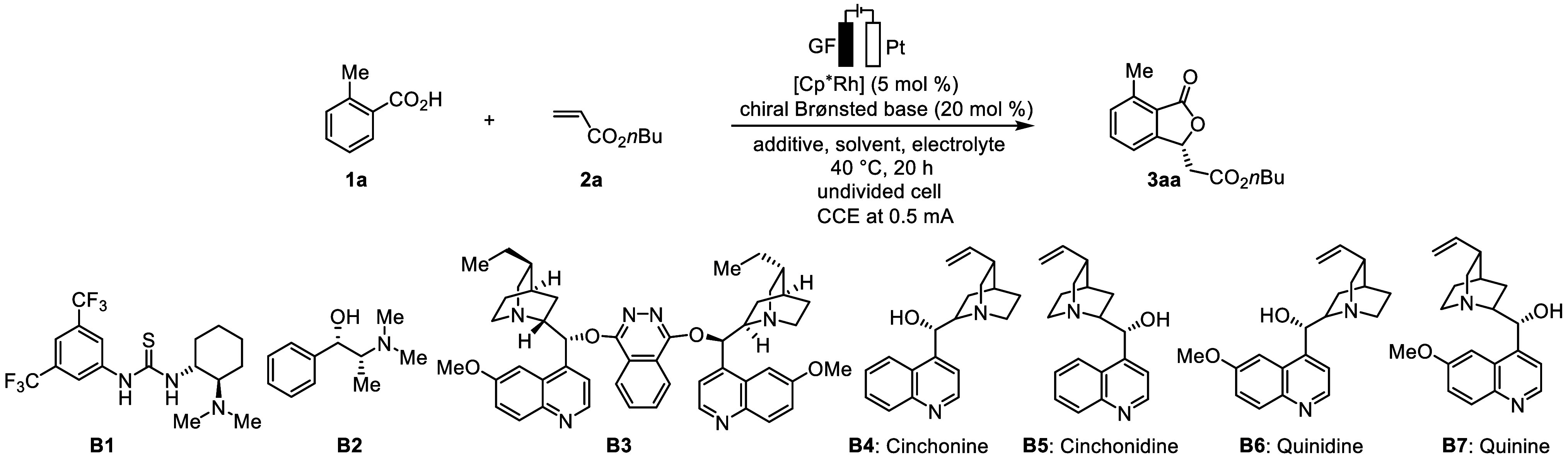
Optimization of the Reaction Conditions^a^

entry	[Cp*Rh]	Brønsted base	additive	solvent	electrolyte	yield (%)	er
1	[Cp*RhCl_2_]_2_	**B1**	NaOPiv	DCE	*n*Bu_4_NPF_6_	0	
2	[Cp*RhCl_2_]_2_	**B2**	NaOPiv	DCE	*n*Bu_4_NPF_6_	<10	
3	[Cp*RhCl_2_]_2_	**B3**	NaOPiv	DCE	*n*Bu_4_NPF_6_	<10	
4	[Cp*RhCl_2_]_2_	**B4**	NaOPiv	DCE	*n*Bu_4_NPF_6_	85	86:14
5	[Cp*RhCl_2_]_2_	**B5**	NaOPiv	DCE	*n*Bu_4_NPF_6_	83	16.5:83.5
6	[Cp*RhCl_2_]_2_	**B6**	NaOPiv	DCE	*n*Bu_4_NPF_6_	78	83.5:16.5
7	[Cp*RhCl_2_]_2_	**B7**	NaOPiv	DCE	*n*Bu_4_NPF_6_	90	20.5:79.5
8	[Cp*RhCl_2_]_2_	**B4**	NaOPiv	DCE:dioxane 3:1	*n*Bu_4_NPF_6_	81	88:12
9	Cp*Rh(OAc)_2_	**B4**		DCE:dioxane 3:1	*n*Bu_4_NPF_6_	75	89.5:10.5
**10**	Cp*Rh(OAc)_2_	**B4**		**CPME**	***n*Bu_4_NBARF**	**86**	**92:8**
11^b^	Cp*Rh(OAc)_2_	**B4**		CPME	*n*Bu_4_NBARF	75	92:8
12		**B4**		CPME	*n*Bu_4_NBARF	0	
13	Cp*Rh(OAc)_2_			CPME	*n*Bu_4_NBARF	28	50:50
14^c^	Cp*Rh(OAc)_2_	**B4**		CPME	*n*Bu_4_NBARF	18	92:8

a Reaction conditions: undivided cell, **1a** (0.20 mmol), **2a** (0.60 mmol), [Cp*Rh] (5 mol %), chiral Brønsted base (20 mol %), additive (20 mol %), electrolyte (0.10 mmol), solvent (4.0 mL), 40 °C, constant current at 0.5 mA, 20 h, graphite felt (GF) anode (10 mm × 15 mm × 6 mm), Pt-plate cathode (10 mm × 15 mm × 0.25 mm). Yield was determined by ^1^H NMR using triphenylmethane as the internal standard. The er value was determined by HPLC. DCE = 1,2-dichloroethane, CPME = cyclopentyl methyl ether, BARF = tetrakis[3,5-bis(trifluoromethyl)phenyl]borate, CCE = constant current electrolysis.

b CCE at 1.0 mA.

c Without electric current.

**Scheme 2:**
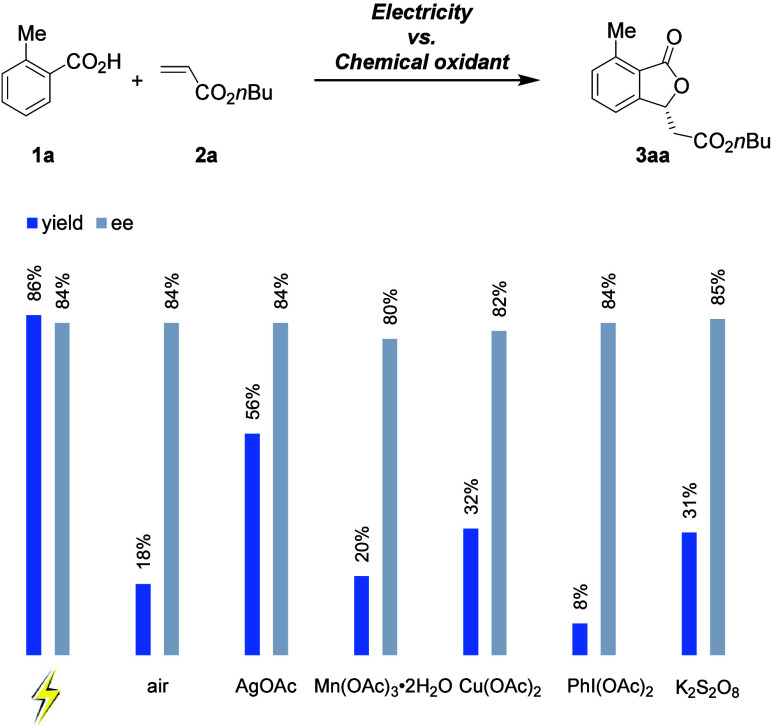
Chemical Yields and Enantioselectivities: Electricity vs Chemical Oxidants

To highlight the merits of the electrochemistry strategy, we conducted control experiments with common chemical oxidants, such as air, AgOAc, Mn(OAc)_3_•2H_2_O, Cu(OAc)_2_, PhI(OAc)_2_, or K_2_S_2_O_8_, in lieu of electricity (Scheme 2). Although high levels of enantioselectivities were achieved, only electrocatalysis delivered high chemical yields.^70^ Thus, electricity is proposed to serve dual roles. On the one hand it is a terminal oxidant, and on the other hand it enables oxidation-induced reductive elimination through a rhodium (III/IV) manifold.^70^

**Scheme 3:**
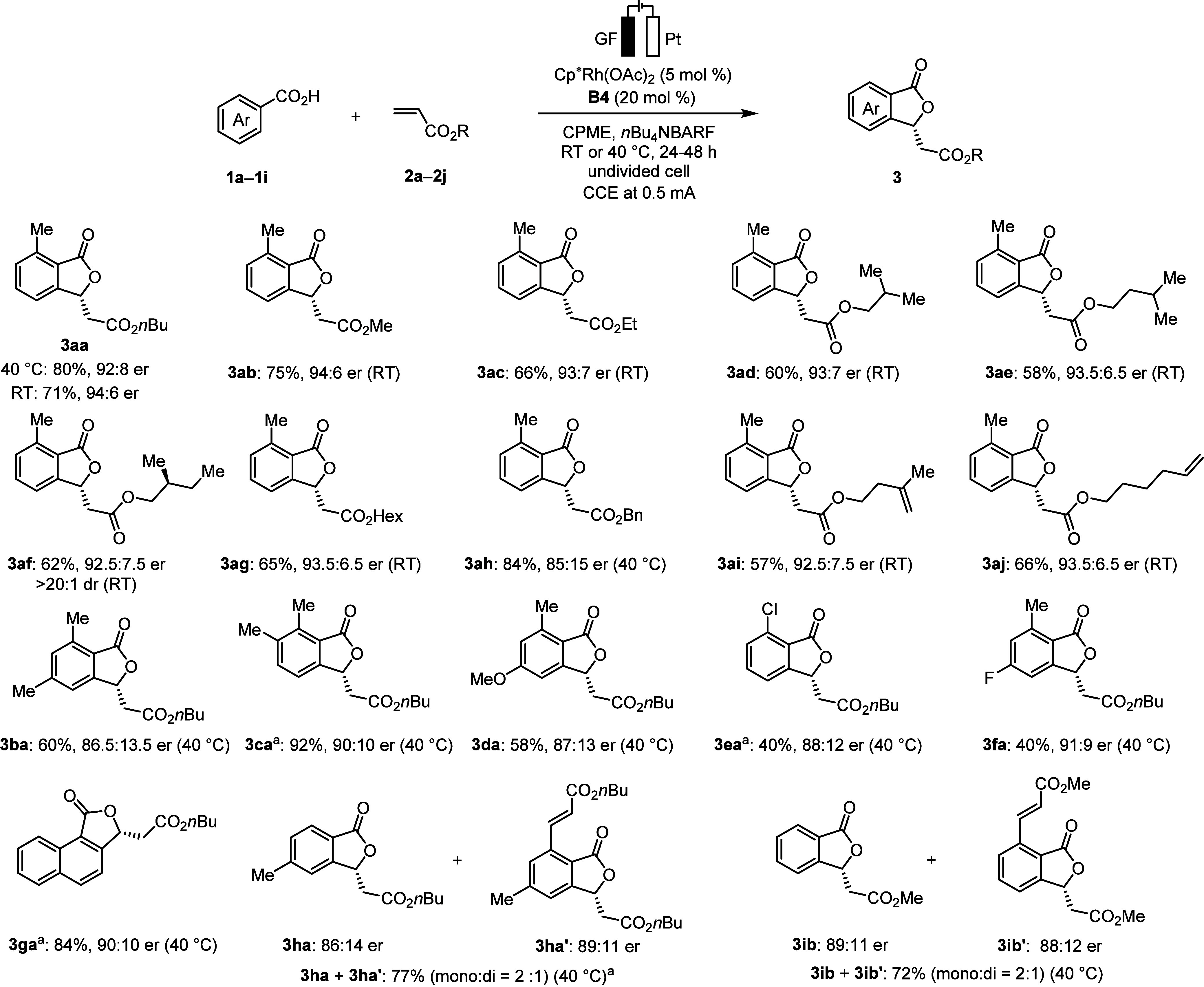
Scope of Electrochemical Domino Catalysis for Enantioselective C–H Annulation DCE:1,4-dioxane (3:1) as solvent instead of CPME.

With the optimal electrocatalysis conditions in hand, we next explored the generality of our approach (Scheme 3). Acrylates with different groups gave the desired chiral phthalides with good enantiomeric ratios (up to 94:6 er). Acrylate **2a** afforded the product **3aa** with a higher enantiomeric ratio at ambient temperature compared with 40 °C, albeit with a slight decrease in yield. Notably, chiral acrylate **2f** provided product **3af** with excellent levels of diastereoselectivity (>20:1 dr) and good er. The reaction with an acrylate substrate bearing two competing olefins selectively delivered a single annulated product (**3ai**, **3aj**). Thereafter, we explored the versatility of electrochemical domino catalysis with various benzoic acids. Different functional groups on the benzoic acids such as methyl (**3ba**, **3ca**), methoxyl (**3da**), chloro (**3ea**), fluoro (**3fa**), and naphthyl (**3ga**), were well tolerated. The *ortho*-unsubstituted benzoic acids with two accessible *ortho*-C–H bonds on treatment with acrylate under the standard conditions delivered products with about 2:1 mono/di ratio and good enantiomeric ratio (**3ha**/**3ha′**, **3ib**/**3ib′**).

**Scheme 4:**
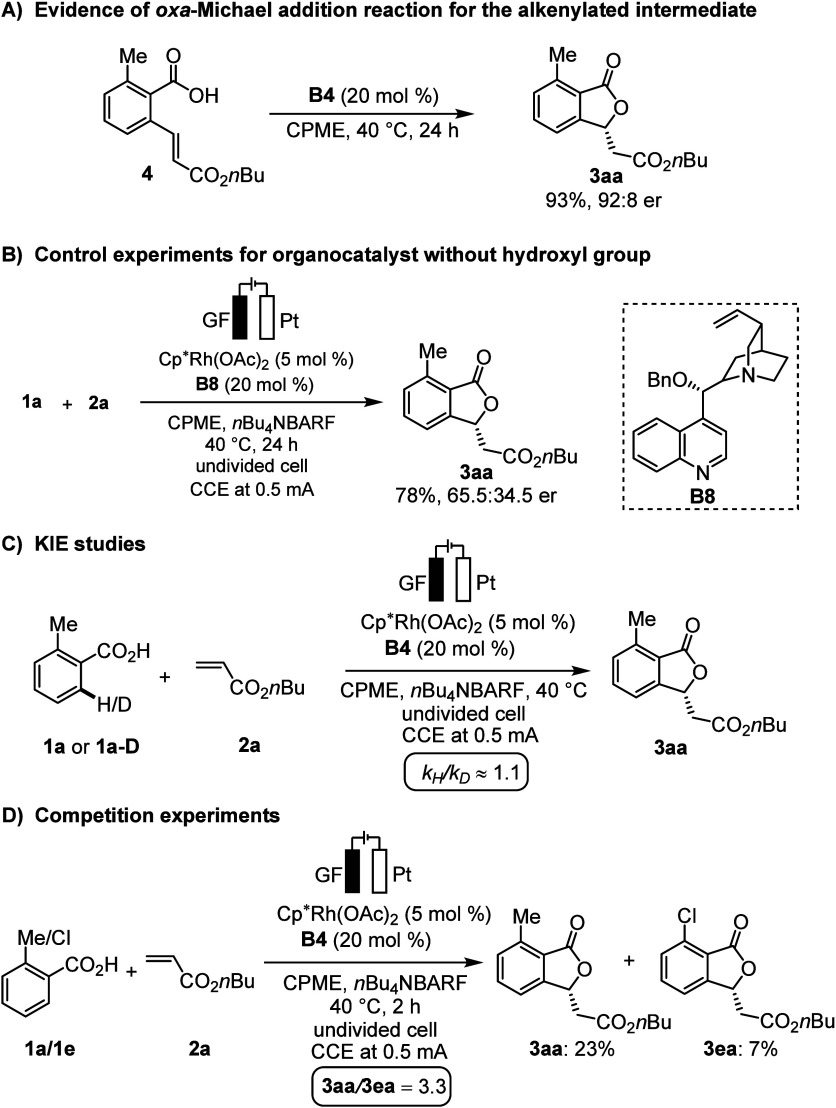
Key Mechanistic Findings

In order to shed light on the modus operandi of our electrochemical domino catalysis, we performed the reaction on alkenylated benzoic acid intermediate **4** using **B4** as a catalyst (Scheme 4A). Product **3aa** was obtained in 93% yield with 92:8 er. This experiment illustrated that the chiral Brønsted base catalyst can activate the intermediate **4** to undergo an enantioselective *oxa*-Michael addition reaction. Utilizing **B8** with a protected alcohol as organocatalyst resulted in a considerably lower enantiomeric ratio (65.5:34.5 er), being supportive of the hydroxyl group serving as hydrogen bond donor (Scheme 4B).^71^ Next, kinetic isotope effect (KIE) studies were performed by parallel reactions of **1a** or **1a-D** with **2a** (Scheme 4C). The KIE of *k*_H_/*k*_D_ ≈ 1.1 suggested that C–H cleavage is not involved in the rate-determining step. Furthermore, we performed competitive experiments using differently substituted benzoic acids, unveiling the intrinsically higher reactivity of electron-rich arenes (Scheme 4D). These observations deviate from a concerted metalation–deprotonation (CMD) mechanism and align more coherently with a base-assisted internal electrophilic-type substitution (BIES) mechanism.^72^

**Scheme 5:**
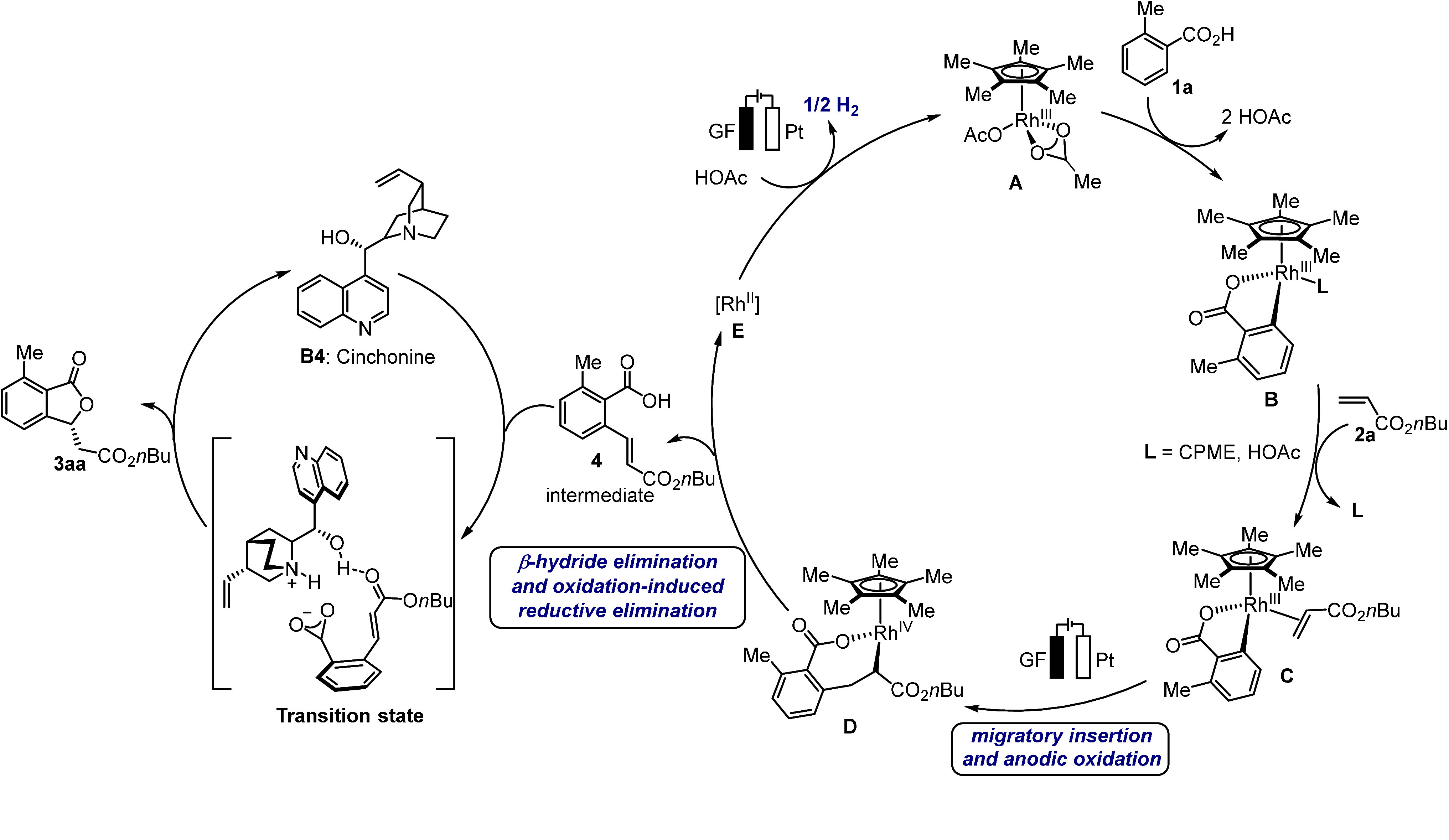
Proposed Catalytic Cycle

Based on our experimental findings, a plausible catalytic cycle is depicted in Scheme 5. The mechanism commences with facile C–H activation by carboxylate assistance, which forms rhodacycle **B**. Thereafter, coordination followed by migratory insertion of the acrylate takes place, which enables the formation of the seven-membered intermediate **D**. Then, an anodic oxidation of rhodium(III), β-hydride elimination and reductive elimination sequence delivers the rhodium(II) complex **E** and the intermediate **4**.^73^ Finally, the anodic oxidation regenerates the active catalytic rhodium(III) complex **A**, while the intermediate **4** undergoes enantioselective *oxa*-Michael addition in the presence of **B4** to afford the chiral product **3aa** through the shown transition state.^74^ An alternative pathway is considered in the Supporting Information.^70^

## Conclusions

In summary, we have reported on an unprecedented electrochemical Cp*-rhodium/chiral Brønsted base-catalyzed enantioselective C–H activation reaction. A mutually compatible dual catalysis system proved essential for the asymmetric domino catalysis, featuring robust, user-friendly achiral Cp*-rhodium catalyst in concert with a readily available chiral Brønsted base. A broad range of benzoic acids were efficiently converted to the desired chiral phthalides. Notably, the catalytic system employed electricity as the sustainable oxidant instead of expensive and toxic silver salts, while generating H_2_ as the only byproduct. These novel findings offer exciting potential for further asymmetric catalysis systems enabled by the merger of achiral transition-metal catalysts and chiral organocatalysts.

## Supporting Information Available

Experimental procedures, optimization studies, mechanistic experiments, characterization of the new compounds, HPLC chromatograms, and NMR spectra (PDF)
