# Supplementary material for: Electrochemical Enantioselective C–H Annulation by Achiral Rhodium(III)/Chiral Brønsted Base Domino Catalysis
Source: ACS Catal. 2024 May 10;14(11):8160–7. doi: 10.1021/acscatal.4c01886 (PMC11165455; doi:10.1021/acscatal.4c01886)
Supplement: Supplementary file 1 — cs4c01886_si_001.pdf [file cs4c01886_si_001.pdf]

Supporting Information

Electrochemical Enantioselective C−H Annulation by Achiral Rhodium(III)/Chiral Brønsted-Base Domino Catalysis

Yanjun Li[1+], Jiawei Xu[1+], João C. A. Oliveira[1], Alexej Scheremetjew[1] & Lutz Ackermann[1]*

[1] Institut für Organische und Biomolekulare Chemie
Georg-August-Universität
Tammannstraße 2, 37077 Göttingen, Germany

[+] These authors contributed equally to this work.

* Corresponding author. E-mail: Lutz.Ackermann@chemie.uni-goettingen.de

Table of Contents

1. General Remarks S3

2. Optimization of the Reaction Conditions S4

3. Substrate Scope S6

4. Key Mechanistic Findings S32

5. An Alternative Catalytic Cycle S36

6. Product Transformation S37

7. References S38

8. NMR-Spectra S39

1. General Remarks

Catalytic reactions were carried out in vials. Substrates acrylates **2f**,[1] **2i**[2] and **2j**[3] were prepared by previously reported methods. Other chemicals were obtained from commercial sources and were used without further purification. Platinum electrodes (10 mm × 15 mm × 0.25 mm, 99.9%; obtained from ChemPur® Karlsruhe, Germany) and graphite felt (GF) electrodes (10 mm × 15 mm × 6 mm, SIGRACELL®GFA 6 EA, obtained from SGL Carbon, Wiesbaden, Germany) were connected using stainless steel adapters. Electrocatalysis was conducted using a Metrohm MULTI AUTOLAB M204 potentiostat or ROHDE & SCHWARZ HMP4040 Potentiostat in two electrode constant current mode. Yields refer to isolated compounds, estimated to be >95% pure as determined by ^{1}H-NMR. TLC: Macherey-Nagel, TLC plates Alugram®Sil G/UV254. Detection under UV light at 254 nm. Chromatography: Separations were carried out on Merck Silica 60 (0.040–0.063 mm, 70–230 mesh ASTM). All IR spectra were recorded on a BRUKER ALPHA-P spectrometer. ESI-MS: Finnigan LCQ. High resolution mass spectrometry (HRMS): APEX IV 7T FTICR, Bruker Daltonic. HPLC chromatograms were recorded on an Agilent 1290 Infinity using CHIRALPAK® IA-3, ID-3, IE-3, and IF-3 columns (3.0 µm particle size; Ø: 4.6 mm and 250 mm length). Optical rotations were measured with Perkin Elmer 343 polarimeter at the stated temperature under a Na/Hg lamp, λ = 589 nm (c in g/100 ml). ^{1}H, ^{13}C, and ^{19}F NMR-spectra were recorded at 300 (^{1}H), 400 (^{1}H), 75, 101 [^{13}C, APT (Attached Proton Test)], and 282, 377 (^{19}F) MHz respectively, on Varian Bruker Avance III 400, Bruker Avance III HD 400 or instruments in $CDCl_3$. If not otherwise specified, chemical shifts (δ) are given in ppm.

2. Optimization of the Reaction Conditions

2.1 Optimization of Catalysts and Solvent

General Procedure: The electrocatalysis was carried out in an undivided cell, with a graphite felt (GF) anode (25 mm × 10 mm × 6.0 mm) and a platinum cathode (25 mm × 10 mm × 0.125 mm). Benzoic acid **1a** (0.20 mmol), acrylate **2a** (0.60 mmol), chiral Brønsted base (0.04 mmol, 20 mol %), electrolyte (0.10 mmol), rhodium catalyst (5 mol %) and solvent (4.0 mL) were placed in a 8 mL cell. Electrocatalysis was performed at 40 °C with a constant current of 0.5 mA maintained for 20 h. Then, the reaction mixture was diluted with EtOAc (2.0 mL). The platinum cathode and the graphite felt anode were washed with EtOAc. The solvents were combined and removed in *vacuo*. Triphenylmethane (48.9 mg, 0.20 mmol) was added as the internal standard to determine the ^{1}H NMR yield. The er value was determined by chiral HPLC analysis.

Table S1. Optimization of the reaction conditions.[a]

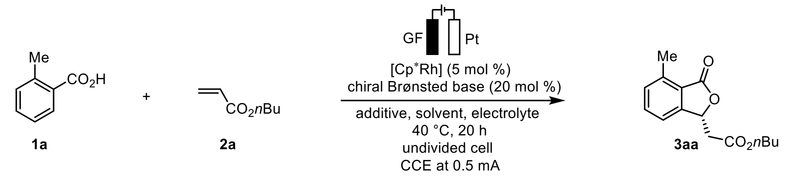

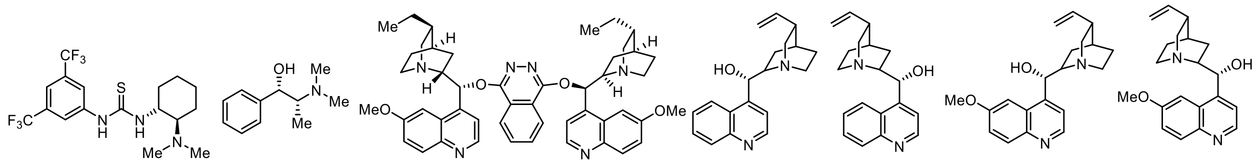

Entry	[Cp*Rh]	Brønsted base	Additive	Solvent	Electrolyte	Yield (%)	er
1	$[Cp^*RhCl_2]_2$	**B1**	NaOPiv	DCE	nBu$_4$NPF$_6$	0	--
2	$[Cp^*RhCl_2]_2$	**B2**	NaOPiv	DCE	nBu$_4$NPF$_6$	<10	--
3	$[Cp^*RhCl_2]_2$	**B3**	NaOPiv	DCE	nBu$_4$NPF$_6$	<10	--
4	$[Cp^*RhCl_2]_2$	**B4**	NaOPiv	DCE	nBu$_4$NPF$_6$	85	86:14
5	$[Cp^*RhCl_2]_2$	**B5**	NaOPiv	DCE	nBu$_4$NPF$_6$	83	16.5:83.5
6	$[Cp^*RhCl_2]_2$	**B6**	NaOPiv	DCE	nBu$_4$NPF$_6$	78	83.5:16.5
7	$[Cp^*RhCl_2]_2$	**B7**	NaOPiv	DCE	nBu$_4$NPF$_6$	90	20.5:79.5
8	$[Cp^*RhCl_2]_2$	**B4**	NaOPiv	DCE: dioxane 3:1	nBu$_4$NPF$_6$	81	88:12
9	$Cp^*Rh(OAc)_2$	**B4**	-	DCE: dioxane 3:1	nBu$_4$NPF$_6$	75	89.5:10.5
10	$Cp^*Rh(OAc)_2$	**B4**	-	CPME	nBu$_4$NBARF	86	92:8
11[b]	$Cp^*Rh(OAc)_2$	**B4**	-	CPME	nBu$_4$NBARF	75	92:8
12	-	**B4**	-	CPME	nBu$_4$NBARF	0	--
13	$Cp^*Rh(OAc)_2$	**-**	-	CPME	nBu$_4$NBARF	28	50:50
14[c]	$Cp^*Rh(OAc)_2$	**B4**	-	CPME	nBu$_4$NBARF	18	92:8

[a] Reaction conditions: undivided cell, **1a** (0.20 mmol), **2a** (0.60 mmol), [Cp*Rh] (5 mol %), chiral Brønsted base (20 mol %), additive (20 mol %), electrolyte (0.10 mmol), solvent (4.0 mL), 40 °C, constant current at 0.5 mA, 20 h, graphite felt (GF) anode (10 mm × 15 mm × 6 mm), Pt-plate cathode (10 mm × 15 mm × 0.25 mm).

Yield was determined by ^{1}H NMR using triphenylmethane as the internal standard. The er value was determined by HPLC. DCE = 1,2-dichloroethane, CPME = cyclopentyl methyl ether, BARF = tetrakis[3,5-bis(trifluoromethyl)phenyl]borate, CCE = constant current electrolysis. [b] CCE at 1.0 mA. [c] Without current.

2.2 Reaction Setup

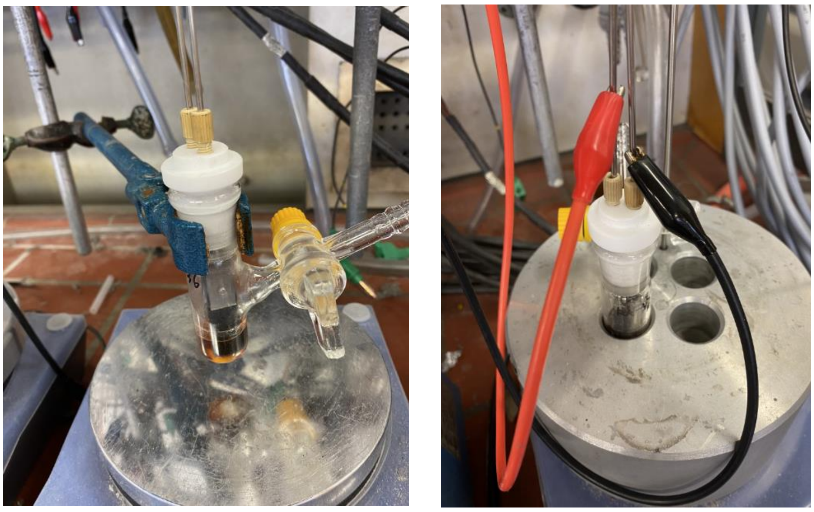

Figure S1. Reaction setup.

2.3 Reaction Comparisons with External Chemical Oxidants

General Procedure: A suspension of benzoic acid **1a** (0.20 mmol), acrylate **2a** (0.60 mmol), **B4** (12.4 mg, 0.04 mmol, 20 mol %), oxidant (0.40 mmol), Cp*Rh(OAc)$_2$ (3.7 mg, 0.01 mmol, 5 mol %) and CPME (4.0 mL) was stirred at 40 °C for 20 h. Chemical oxidants used were AgOAc, Mn(OAc)$_3$·2H$_2$O, Cu(OAc)$_2$, PhI(OAc)$_2$, and K$_2$S$_2$O$_8$. Additionally, the same reaction was performed under air as a terminal oxidant. Triphenylmethane (48.9 mg, 0.20 mmol) was added as the internal standard to determine the ^{1}H NMR yield.

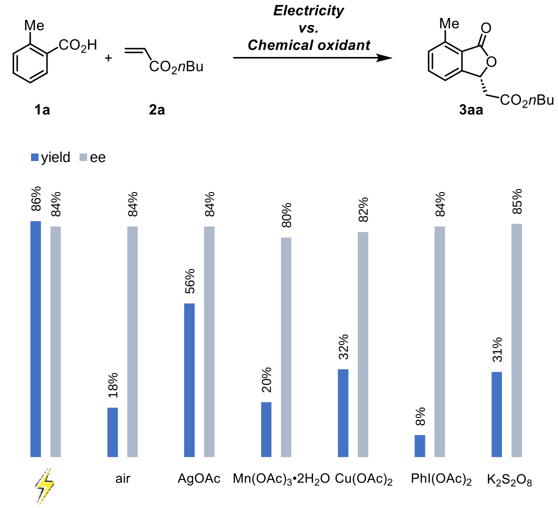

Figure S2. Yield and enantioselectivity comparison between electricity and chemical oxidants.

3. Substrate Scope

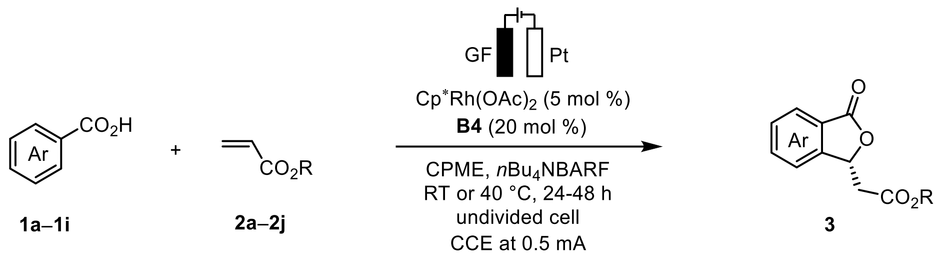

General Procedure: The electrocatalysis was carried out in an undivided cell, with a graphite felt (GF) anode (25 mm × 10 mm × 6.0 mm) and a platinum cathode (25 mm × 10 mm × 0.125 mm). Benzoic acid (0.20 mmol), acrylate (0.60 mmol), **B4** (12.4 mg, 0.04 mmol, 20 mol %), *n*Bu_4NBARF (110.0 mg, 0.20 mmol), Cp*Rh$(OAc)_2$ (3.7 mg, 0.01 mmol, 5 mol %) and CPME (4.0 mL) were placed in a 8 mL cell. Electrocatalysis was performed at 40 °C or room temperature with a constant current of 0.5 mA maintained for 24-48 h. Then, the reaction mixture was diluted with EtOAc (2.0 mL). The platinum cathode and the graphite felt anode were washed with EtOAc. Evaporation of the solvent and subsequent column chromatography on silica gel afforded the corresponding products.

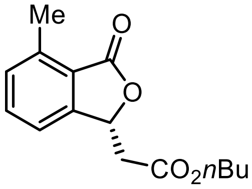

3aa

butyl (*R*)-2-(4-methyl-3-oxo-1,3-dihydroisobenzofuran-1-yl)acetate (3aa)

When performed at 40 °C: the general procedure was followed using benzoic acid **1a** (27.2 mg, 0.20 mmol), acrylate **2a** (76.8 mg, 0.60 mmol) at 40 °C for 24 h. Purification by column chromatography on silica gel (*n*-hexane/EtOAc: 5/1) yielded **3aa** (42.0 mg, 80%) as a yellow oil. ^{1}H NMR (300 MHz, $CDCl_3$) δ 7.53 (t, *J* = 7.6 Hz, 1H), 7.39 – 7.21 (m, 2H), 5.81 (t, *J* = 6.5 Hz, 1H), 4.15 (t, *J* = 6.7 Hz, 2H), 2.87 (d, *J* = 6.6 Hz, 2H), 2.69 (s, 3H), 1.62 (tt, *J* = 8.5, 5.2 Hz, 2H), 1.36 (h, *J* = 7.3 Hz, 2H), 0.93 (t, *J* = 7.3 Hz, 3H). ^{13}C NMR (75 MHz, $CDCl_3$) δ 170.10 (C_q), 169.47 (C_q), 149.29 (C_q), 139.94 (C_q), 133.96 (CH), 131.13 (CH), 123.42 (C_q), 119.26 (CH), 76.09 (CH), 65.15 (CH_2), 39.78 (CH_2), 30.52 (CH_2), 19.08 (CH_2), 17.38 (CH_3), 13.70 (CH_3). $[\alpha_D^{20}]$ = +2.7 (c = 2.1, CH_2Cl_2). HPLC separation (Chiralpak® IE-3, *n*-hexane/*i*-PrOH 80:20, 1.0 mL/min, detection at 273 nm): t_r (major) = 17.9 min, t_r (minor) = 20.2 min, 92:8 er.

Spectral data were consistent with data reported in the literature.[4]

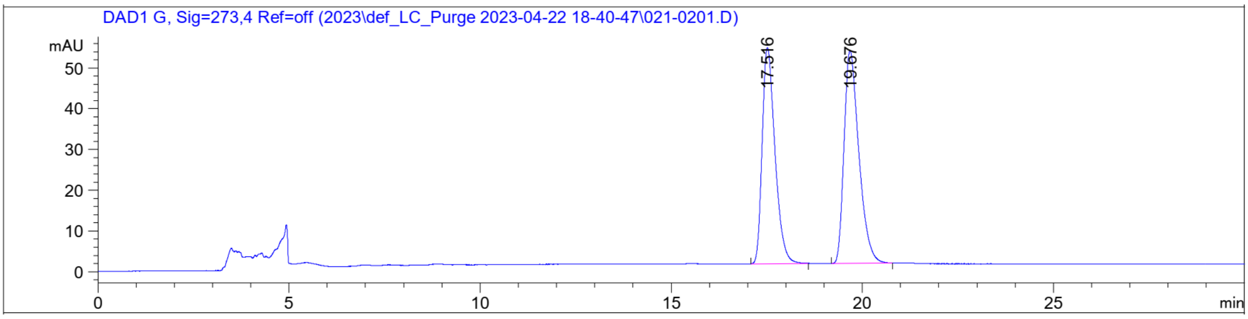

Peak #	RetTime [min]	Type	Width [min]	Area [mAU*s]	Height [mAU]	Area %
1	17.516	BB	0.3342	287.54520	12.36840	46.5087
2	19.677	BB	0.3694	330.71579	12.23245	53.4913

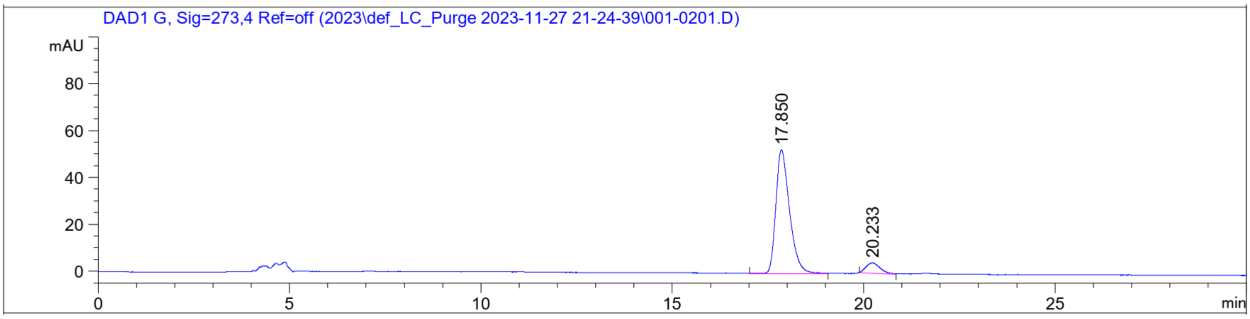

Peak #	RetTime [min]	Type	Width [min]	Area [mAU*s]	Height [mAU]	Area %
1	17.850	MM R	0.3112	1328.66382	52.91357	91.9461
2	20.233	MM R	0.4337	116.38187	4.47278	8.0539

When performed at room temperature: the general procedure was followed using benzoic acid **1a** (27.2 mg, 0.20 mmol), acrylate **2a** (76.8 mg, 0.60 mmol) at room temperature for 36 h. Purification by column chromatography on silica gel (*n*-hexane/EtOAc: 5/1) yielded **3aa** (37.2 mg, 71%) as a yellow oil. HPLC separation (Chiralpak® IE-3, *n*-hexane/*i*-PrOH 80:20, 1.0 mL/min, detection at 273 nm): t_r (major) = 17.7 min, t_r (minor) = 20.0 min, 94:6 er.

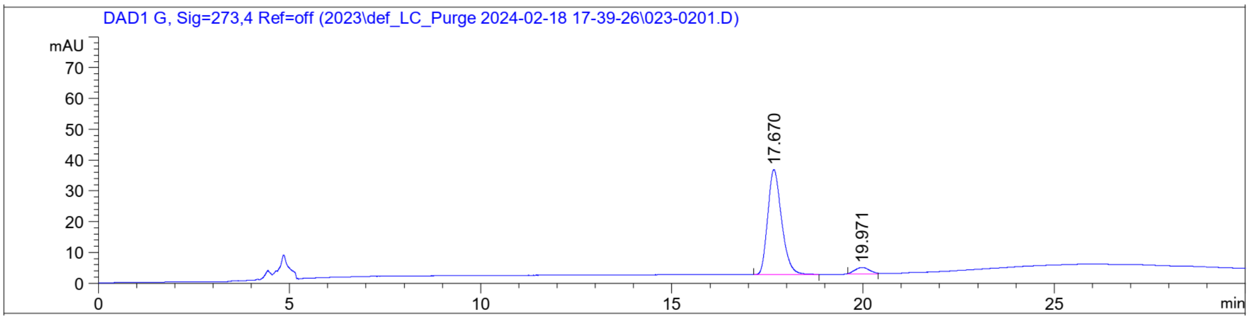

Peak #	RetTime [min]	Type	Width [min]	Area [mAU*s]	Height [mAU]	Area %
1	17.670	BB	0.3760	860.01587	34.13165	93.9595
2	19.971	MM R	0.4425	55.28857	2.08259	6.0405

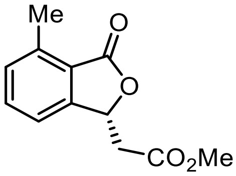

3ab

methyl (*R*)-2-(4-methyl-3-oxo-1,3-dihydroisobenzofuran-1-yl)acetate (3ab)

The general procedure was followed using benzoic acid **1a** (27.2 mg, 0.20 mmol), acrylate **2b** (51.7 mg, 0.60 mmol) at room temperature for 36 h. Purification by column chromatography on silica gel (*n*-hexane/EtOAc: 5/1) yielded **3ab** (33.0 mg, 75%) as a yellow oil. ^{1}H NMR (300 MHz, $CDCl_3$) δ 7.52 (t, J = 7.6 Hz, 1H), 7.33 – 7.23 (m, 2H), 5.80 (t, J = 6.6 Hz, 1H), 3.75 (s, 3H), 2.86 (d, J = 6.6 Hz, 2H), 2.67 (s, 3H). ^{13}C NMR (75 MHz, $CDCl_3$) δ 170.00 (C_q), 169.83 (C_q), 149.19 (C_q), 139.95 (C_q), 134.00 (CH), 131.17 (CH), 123.34 (C_q), 119.25 (CH), 76.01 (CH), 52.19 (CH_3), 39.62 (CH_2), 17.34 (CH_3). $[\alpha^{20}_{D}]$ = +17.9 (c = 1.5, CH_2Cl_2). HPLC separation (Chiralpak® ID-3, *n*-hexane/*i*-PrOH 80:20, 1.0 mL/min, detection at 273 nm): t_r (major) = 16.2 min, t_r (minor) = 26.3 min, 94:6 er.

Spectral data were consistent with data reported in the literature.[5]

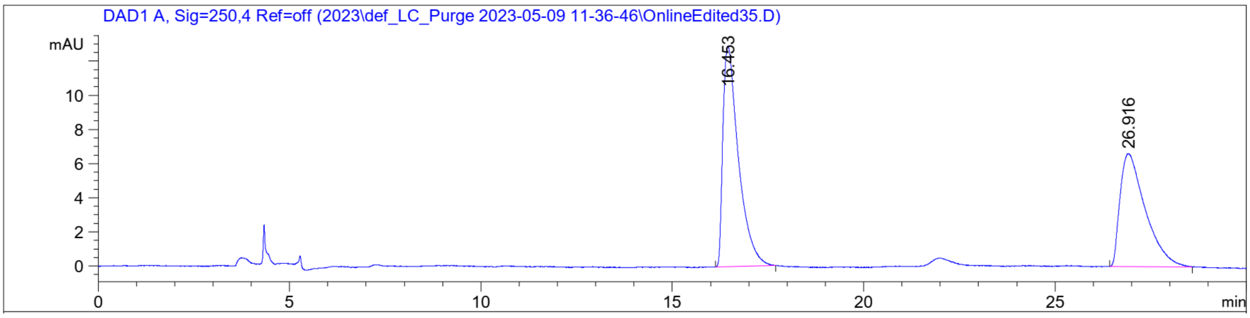

Peak #	RetTime [min]	Type	Width [min]	Area [mAU*s]	Height [mAU]	Area %
1	16.453	BB	0.4007	365.09241	12.91898	54.6585
2	26.916	BB	0.5370	302.85977	6.61696	45.3415

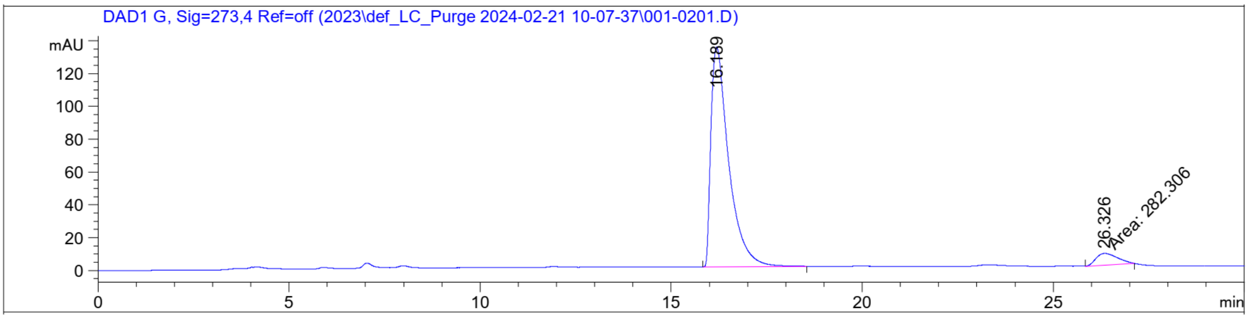

Peak #	RetTime [min]	Type	Width [min]	Area [mAU*s]	Height [mAU]	Area %
1	16.189	BB	0.4869	4328.56592	133.98424	93.8774
2	26.326	MM	0.4682	282.30603	7.20515	6.1226

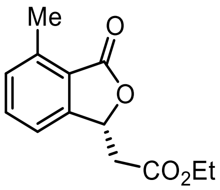

3ac

ethyl (*R*)-2-(4-methyl-3-oxo-1,3-dihydroisobenzofuran-1-yl)acetate (3ac)

The general procedure was followed using benzoic acid **1a** (27.2 mg, 0.20 mmol), acrylate **2c** (60.0 mg, 0.60 mmol) at room temperature for 36 h. Purification by column chromatography on silica gel (*n*-hexane/EtOAc: 5/1) yielded **3ac** (30.9 mg, 66%) as a yellow oil. ^{1}H NMR (300 MHz, $CDCl_3$) δ 7.52 (t, J = 7.6 Hz, 1H), 7.31 – 7.21 (m, 2H), 5.80 (t, J = 6.5 Hz, 1H), 4.20 (q, J = 7.2 Hz, 2H), 2.85 (d, J = 6.5 Hz, 2H), 2.67 (s, 3H), 1.25 (t, J = 7.1 Hz, 3H). ^{13}C NMR (75 MHz, $CDCl_3$) δ 170.07 (C_q), 169.35 (C_q), 149.28 (C_q), 139.90 (C_q), 133.96 (CH), 131.12 (CH), 123.40 (C_q), 119.27 (CH), 76.08 (CH), 61.22 (CH_2), 39.80 (CH_2), 17.34 (CH_3), 14.12 (CH_3). $[\alpha]_D^{20}$ = +20.1 (c = 1.5, CH_2Cl_2). HPLC separation (Chiralpak® IE-3, *n*-hexane/*i*-PrOH 80:20, 1.0 mL/min, detection at 273 nm): t_r (major) = 20.9 min, t_r (minor) = 27.1 min, 93:7 er.

Spectral data were consistent with data reported in the literature.[4]

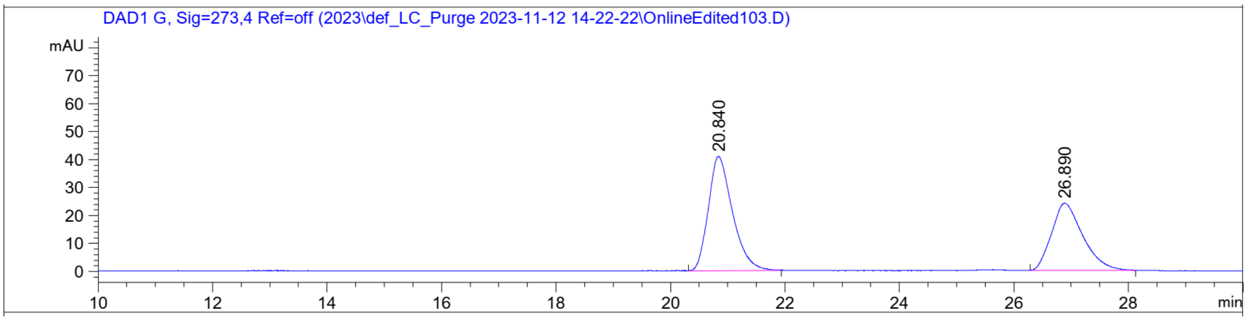

Peak #	RetTime [min]	Type	Width [min]	Area [mAU*s]	Height [mAU]	Area %
1	20.840	BB	0.4037	1192.57275	40.83255	56.7210
2	26.890	BB	0.4484	909.95111	24.05344	43.2790

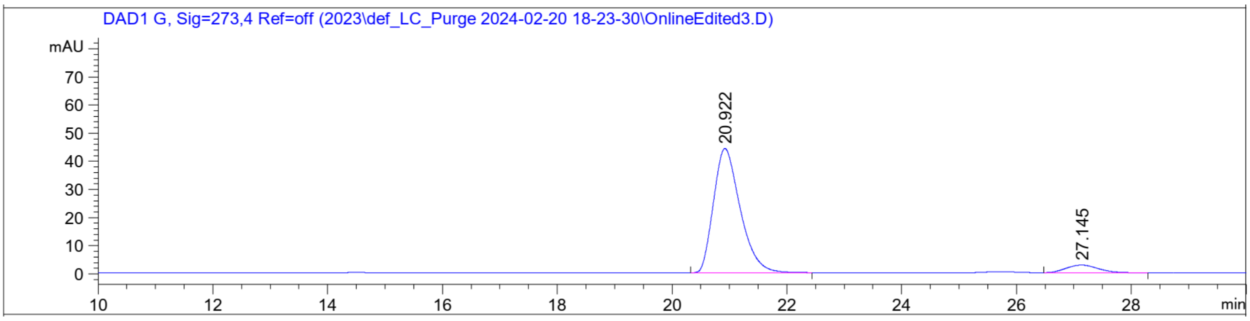

Peak #	RetTime [min]	Type	Width [min]	Area [mAU*s]	Height [mAU]	Area %
1	20.922	BB	0.4845	1424.37671	44.13440	93.1364
2	27.145	BB	0.4591	104.96731	2.70288	6.8636

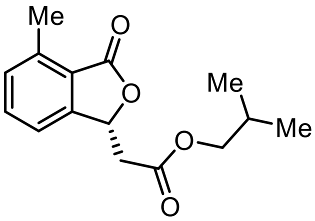

3ad

isobutyl (*R*)-2-(4-methyl-3-oxo-1,3-dihydroisobenzofuran-1-yl)acetate (3ad)

The general procedure was followed using benzoic acid **1a** (27.2 mg, 0.20 mmol), acrylate **2d** (76.8 mg, 0.60 mmol) at room temperature for 48 h. Purification by column chromatography on silica gel (*n*-hexane/EtOAc: 5/1) yielded **3ad** (31.5 mg, 60%) as a yellow oil. ^{1}H NMR (300 MHz, $CDCl_3$) δ 7.31 (t, J = 7.6 Hz, 1H), 7.10 – 7.04 (m, 2H), 5.60 (t, J = 6.5 Hz, 1H), 3.73 (dd, J = 6.7, 0.8 Hz, 2H), 2.67 (d, J = 6.6 Hz, 2H), 2.47 (s, 3H), 1.72 (dt, J = 13.4, 6.7 Hz, 1H), 0.71 (dd, J = 6.7, 1.9 Hz, 6H). ^{13}C NMR (75 MHz, $CDCl_3$) δ 170.05 (C_q), 169.42 (C_q), 149.28 (C_q), 139.91 (C_q), 133.96 (CH), 131.12 (CH), 123.41 (C_q), 119.25 (CH), 76.08 (CH), 71.30 (CH_2), 39.75 (CH_2), 27.64 (CH_3), 19.04 (CH_3), 17.34 (CH_3). $[\alpha^{20}_{D}]$ = +7.9 (c = 1.5, CH_2Cl_2). HPLC separation (Chiralpak® IE-3, *n*-hexane/*i*-PrOH 80:20, 1.0 mL/min, detection at 273 nm): t_r (major) = 15.9 min, t_r (minor) = 18.3 min, 93:7 er.

Spectral data were consistent with data reported in the literature.[5]

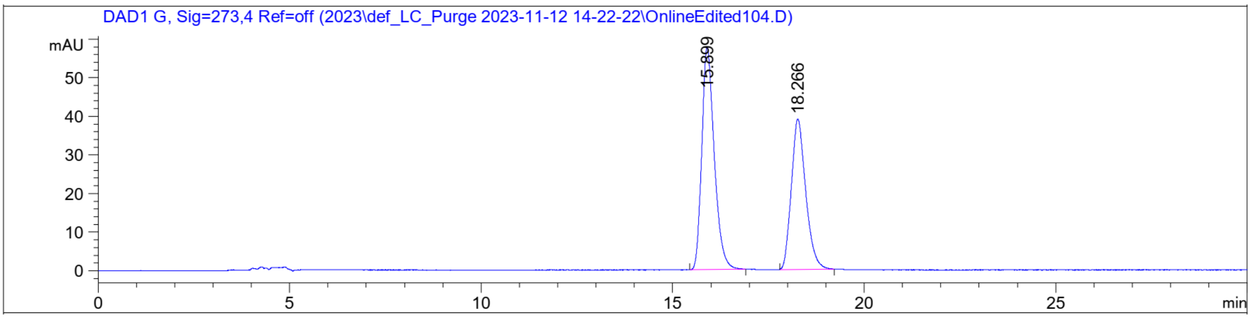

Peak #	RetTime [min]	Type	Width [min]	Area [mAU*s]	Height [mAU]	Area %
1	15.899	BB	0.3276	1303.91174	57.69933	56.2131
2	18.266	BB	0.3661	1015.67505	38.96603	43.7869

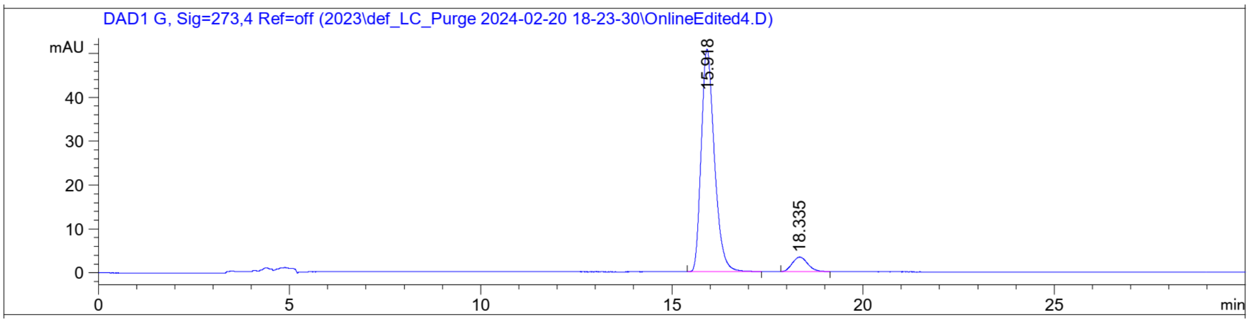

Peak #	RetTime [min]	Type	Width [min]	Area [mAU*s]	Height [mAU]	Area %
1	15.918	BB	0.3654	1214.52612	50.72168	92.9502
2	18.335	BB	0.3329	92.11553	3.29760	7.0498

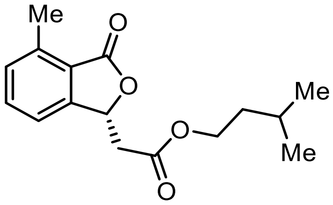

3ae

isopentyl (*R*)-2-(4-methyl-3-oxo-1,3-dihydroisobenzofuran-1-yl)acetate (3ae)

The general procedure was followed using benzoic acid **1a** (27.2 mg, 0.20 mmol), acrylate **2e** (85.3 mg, 0.60 mmol) at room temperature for 48 h. Purification by column chromatography on silica gel (*n*-hexane/EtOAc: 5/1) yielded **3ae** (32.1 mg, 58%) as a yellow oil. ^{1}H NMR (300 MHz, $CDCl_3$) δ 7.51 (t, J = 7.6 Hz, 1H), 7.31 – 7.24 (m, 2H), 5.79 (t, J = 6.5 Hz, 1H), 4.16 (t, J = 6.9 Hz, 2H), 2.85 (d, J = 6.5 Hz, 2H), 2.67 (s, 3H), 1.72 – 1.56 (m, 1H), 1.50 (q, J = 7.0 Hz, 2H), 0.90 (d, J = 6.6 Hz, 6H). ^{13}C NMR (75 MHz, $CDCl_3$) δ 170.04 (C_q), 169.40 (C_q), 149.27 (C_q), 139.88 (C_q), 133.94 (CH), 131.10 (CH), 123.41 (C_q), 119.25 (CH), 76.06 (CH), 63.89 (CH_2), 39.77 (CH_2), 37.16 (CH_2), 24.97 (CH), 22.42 (CH_3), 22.39 (CH_3), 17.32 (CH_3). IR (ATR): 2961, 1756, 1602,

1469, 1382, 1163, 1008, 787 cm^{-1}. MS (ESI) *m/z* (relative intensity): 299 (100) $[M + Na]^+$. HR-MS (ESI): *m/z* calcd. for $[C_{16}H_{20}O_4 + Na]^+$ 299.1254, found 299.1254. $[\alpha_D^{20}]$ = +21.0 (c = 1.5, CH_2Cl_2). HPLC separation (Chiralpak® IE-3, *n*-hexane/*i*-PrOH 80:20, 1.0 mL/min, detection at 273 nm): t_r (major) = 15.9 min, t_r (minor) = 17.7 min, 93.5:6.5 er.

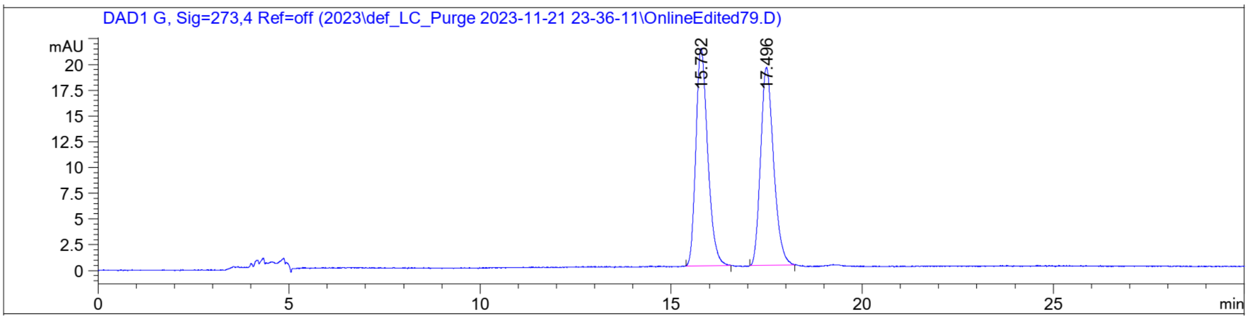

Peak #	RetTime [min]	Type	Width [min]	Area [mAU*s]	Height [mAU]	Area %
1	15.782	BB	0.2902	454.28854	21.09587	49.9469
2	17.496	BB	0.3355	455.25418	19.20983	50.0531

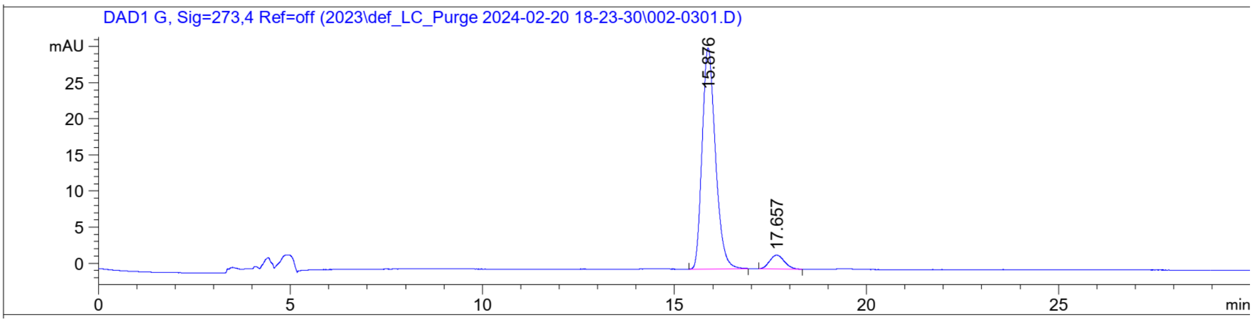

Peak #	RetTime [min]	Type	Width [min]	Area [mAU*s]	Height [mAU]	Area %
1	15.876	BB	0.3641	734.65247	30.60440	93.5492
2	17.657	BB	0.3143	50.65851	1.90457	6.4508

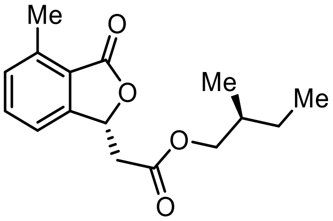

3af

(*S*)-2-methylbutyl 2-((*R*)-4-methyl-3-oxo-1,3-dihydroisobenzofuran-1-yl)acetate (3af)

The general procedure was followed using benzoic acid **1a** (27.2 mg, 0.20 mmol), acrylate **2f** (85.2 mg, 0.60 mmol) at room temperature for 48 h. Purification by column chromatography on silica gel (*n*-hexane/EtOAc: 5/1) yielded **3af** (34.3 mg, 62%, >20:1 dr) as a yellow oil. ^{1}H NMR (400 MHz, $CDCl_3$) δ 7.52 (t, *J* = 7.6 Hz, 1H), 7.34 – 7.24 (m, 2H), 5.80 (t, *J* = 6.5 Hz, 1H), 4.04 (dd, *J*

= 10.7, 6.0 Hz, 1H), 3.94 (dd, *J* = 10.7, 6.7 Hz, 1H), 2.88 (d, *J* = 6.6 Hz, 2H), 2.67 (s, 3H), 1.75 – 1.65 (m, 1H), 1.40 (ddd, *J* = 15.0, 7.5, 5.6 Hz, 1H), 1.22 – 1.10 (m, 1H), 0.89 (dd, *J* = 8.4, 7.0 Hz, 6H). ^{13}C NMR (101 MHz, $CDCl_3$) δ 170.06 (C_q), 169.48 (C_q), 149.30 (C_q), 139.92 (C_q), 133.97 (CH), 131.13 (CH), 123.42 (C_q), 119.26 (CH), 76.09 (CH), 69.88 (CH_2), 39.77 (CH_2), 34.03 (CH), 25.96 (CH_2), 17.35 (CH_3), 16.33 (CH_3), 11.19 (CH_3). IR (ATR): 2962, 1757, 1602, 1462, 1290, 1165, 1008, 788 cm^{-1}. MS (ESI) *m/z* (relative intensity): 299 (100) [M + Na]$^+$. HR-MS (ESI): *m/z* calcd. for $[C_{16}H_{20}O_4 + Na]^+$ 299.1254, found 299.1257. $[\alpha]_D^{20}$ = +21.1 (c = 1.4, CH_2Cl_2). HPLC separation (Chiralpak® IE-3, *n*-hexane/*i*-PrOH 80:20, 1.0 mL/min, detection at 273 nm): t_r (major) = 15.4 min, t_r (minor) = 17.9 min, 92.5:7.5 er.

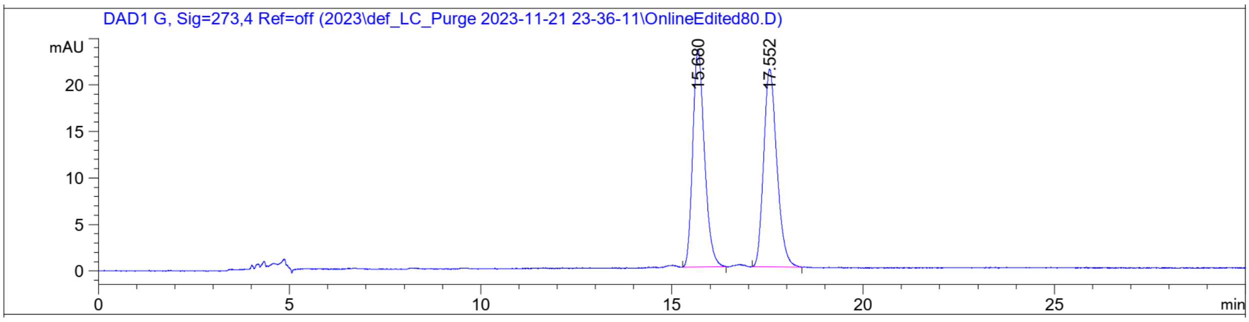

Peak #	RetTime [min]	Type	Width [min]	Area [mAU*s]	Height [mAU]	Area %
1	15.680	BB	0.3036	499.30734	23.42753	49.4387
2	17.552	BB	0.3194	510.64462	21.24870	50.5613

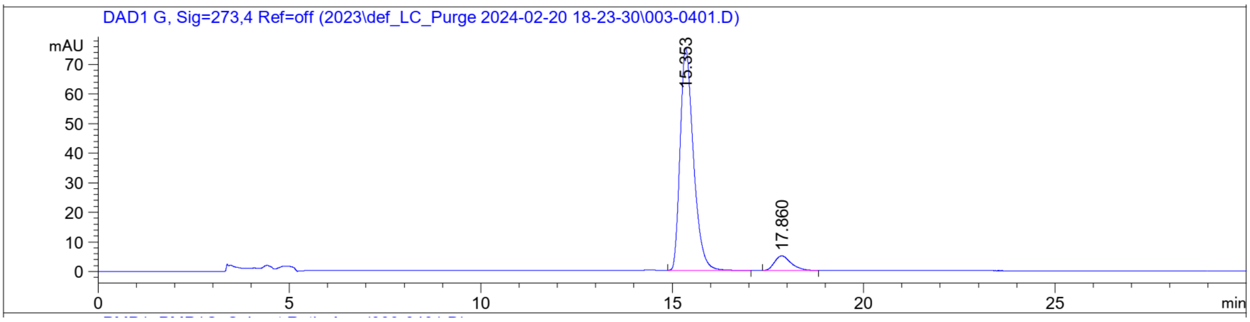

Peak #	RetTime [min]	Type	Width [min]	Area [mAU*s]	Height [mAU]	Area %
1	15.353	BB	0.3627	1817.06580	75.24091	92.4097
2	17.860	BB	0.3960	149.24893	4.94697	7.5903

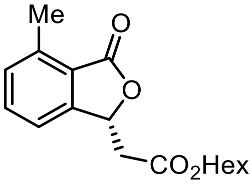

3ag

hexanoic (*R*)-2-(4-methyl-3-oxo-1,3-dihydroisobenzofuran-1-yl)acetic anhydride (3ag)

The general procedure was followed using benzoic acid **1a** (27.2 mg, 0.20 mmol), acrylate **2g** (102.1 mg, 0.60 mmol) at room temperature for 48 h. Purification by column chromatography on silica gel (*n*-hexane/EtOAc: 5/1) yielded **3ag** (39.6 mg, 65%) as a yellow oil. ^{1}H NMR (300 MHz, $CDCl_3$) δ 7.52 (t, *J* = 7.6 Hz, 1H), 7.31 – 7.26 (m, 2H), 5.80 (t, *J* = 6.5 Hz, 1H), 4.14 (t, *J* = 6.8 Hz, 2H), 2.87 (d, *J* = 6.5 Hz, 2H), 2.68 (s, 3H), 1.61 (dt, *J* = 8.3, 6.7 Hz, 2H), 1.37 – 1.26 (m, 6H), 0.94 – 0.80 (m, 3H). ^{13}C NMR (75 MHz, $CDCl_3$) δ 170.05 (C_q), 169.43 (C_q), 149.29 (C_q), 139.90 (C_q), 133.94 (CH), 131.11 (CH), 123.41 (C_q), 119.26 (CH), 76.08 (CH), 65.41 (CH_2), 39.77 (CH_2), 31.38 (CH_2), 28.46 (CH_2), 25.50 (CH_2), 22.50 (CH_2), 17.34 (CH_3), 13.99 (CH_3). $[\alpha^{20}_{D}]$ = +7.4 (c = 2.0, CH_2Cl_2). HPLC separation (Chiralpak® IE-3, *n*-hexane/*i*-PrOH 80:20, 1.0 mL/min, detection at 273 nm): t_r (major) = 15.7 min, t_r (minor) = 17.7 min, 93.5:6.5 er.

Spectral data were consistent with data reported in the literature.[4]

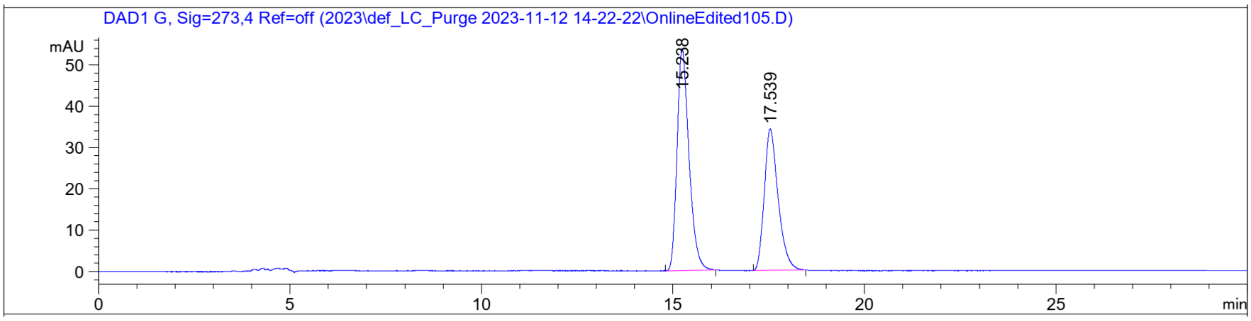

Peak #	RetTime [min]	Type	Width [min]	Area [mAU*s]	Height [mAU]	Area %
1	15.238	BB	0.3145	1162.86243	53.72841	57.4414
2	17.539	BB	0.3534	861.57037	34.26498	42.5586

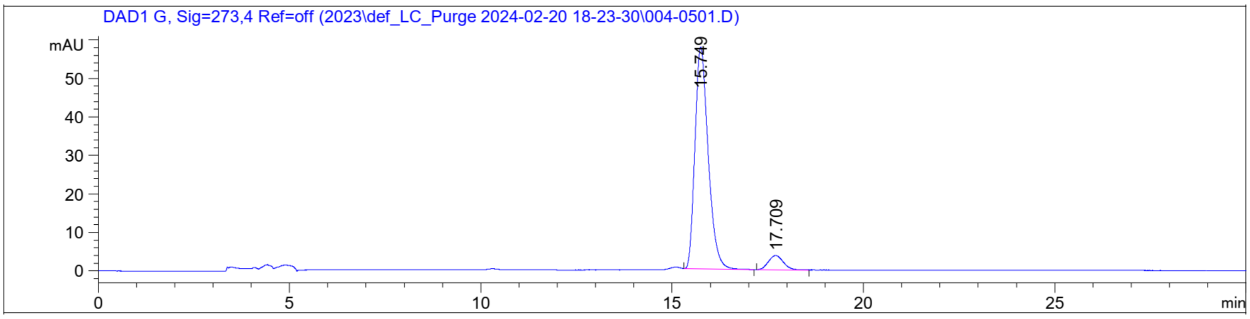

Peak #	RetTime [min]	Type	Width [min]	Area [mAU*s]	Height [mAU]	Area %
1	15.749	BB	0.3663	1385.23560	57.64788	93.3647
2	17.709	BB	0.3192	98.44675	3.67868	6.6353

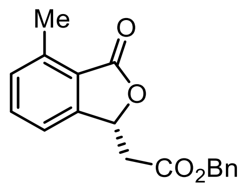

3ah

benzyl (*R*)-2-(4-methyl-3-oxo-1,3-dihydroisobenzofuran-1-yl)acetate (3ah)

The general procedure was followed using benzoic acid **1a** (27.2 mg, 0.20 mmol), acrylate **2h** (97.3 mg, 0.60 mmol) at 40 °C for 24 h. Purification by column chromatography on silica gel (*n*-hexane/EtOAc: 5/1) yielded **3ah** (49.7 mg, 84%) as a yellow oil. ^{1}H NMR (300 MHz, $CDCl_3$) δ 7.49 (t, *J* = 7.6 Hz, 1H), 7.39 – 7.31 (m, 5H), 7.30 – 7.25 (m, 1H), 7.20 (dd, *J* = 7.8, 1.0 Hz, 1H), 5.82 (t, *J* = 6.5 Hz, 1H), 5.19 (d, *J* = 2.7 Hz, 2H), 2.92 (dd, *J* = 6.5, 1.1 Hz, 2H), 2.67 (s, 3H). ^{13}C NMR (75 MHz, $CDCl_3$) δ 170.00 (C_q), 169.18 (C_q), 149.14 (C_q), 139.94 (C_q), 135.27 (C_q), 133.97 (CH), 131.15 (CH), 128.64 (CH), 128.50 (CH), 128.45 (CH), 123.37 (C_q), 119.26 (CH), 75.97 (CH), 67.02 (CH_2), 39.78 (CH_2), 17.35 (CH_3). $[\alpha]_D^{20}$ = +3.1 (c = 2, $CHCl_3$). HPLC separation (Chiralpak® ID-3, *n*-hexane/*i*-PrOH 80:20, 1.0 mL/min, detection at 273 nm): t_r (major) = 19.4 min, t_r (minor) = 24.6 min, 85:15 er.

Spectral data were consistent with data reported in the literature.[5]

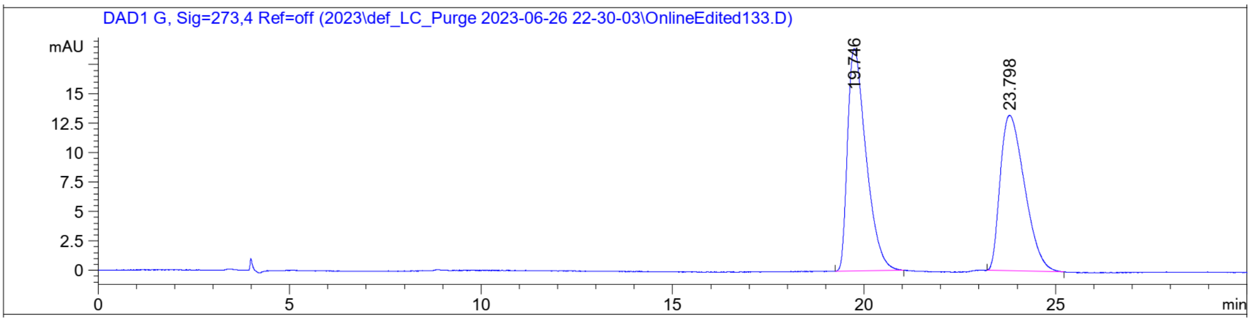

Peak #	RetTime [min]	Type	Width [min]	Area [mAU*s]	Height [mAU]	Area %
1	19.746	BB	0.4707	637.00623	18.92438	52.1237
2	23.798	BB	0.5227	585.09912	13.23612	47.8763

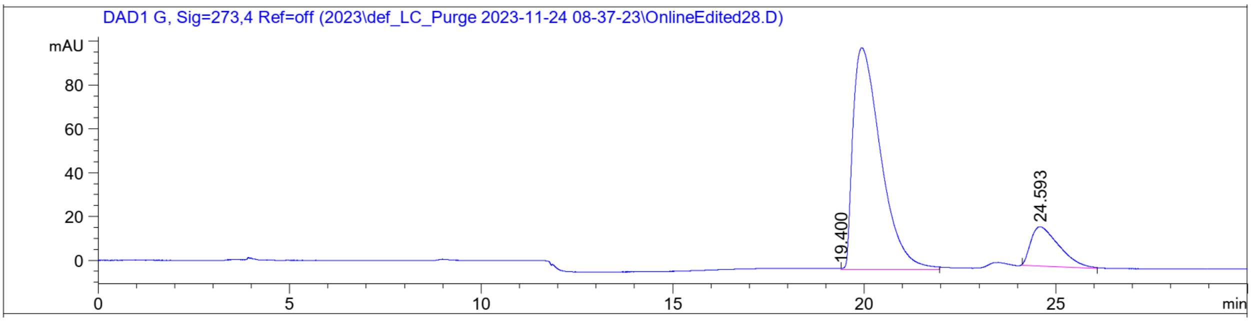

Peak #	RetTime [min]	Type	Width [min]	Area [mAU*s]	Height [mAU]	Area %
1	19.400	MM R	0.5063	5117.23828	5.51438e-1	84.7688
2	24.593	MM R	0.6645	919.46375	17.91640	15.2312

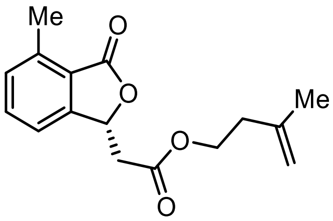

3ai

3-methylbut-3-en-1-yl (*R*)-2-(4-methyl-3-oxo-1,3-dihydroisobenzofuran-1-yl)acetate (3ai)

The general procedure was followed using benzoic acid **1a** (27.2 mg, 0.20 mmol), acrylate **2i** (84.1 mg, 0.60 mmol) at room temperature for 48 h. Purification by column chromatography on silica gel (*n*-hexane/EtOAc: 5/1) yielded **3ai** (31.3 mg, 57%) as a yellow oil. ^{1}H NMR (300 MHz, $CDCl_3$) δ 7.52 (t, *J* = 7.6 Hz, 1H), 7.33 – 7.25 (m, 2H), 5.80 (t, *J* = 6.5 Hz, 1H), 4.83 – 4.71 (m, 2H), 4.27 (td, *J* = 6.9, 1.0 Hz, 2H), 2.86 (d, *J* = 6.6 Hz, 2H), 2.68 (s, 3H), 2.40 – 2.31 (m, 2H), 1.75 (t, *J* = 1.1 Hz, 3H). ^{13}C NMR (75 MHz, $CDCl_3$) δ 170.03 (C_q), 169.33 (C_q), 149.25 (C_q), 141.28 (C_q), 139.91 (C_q), 133.95 (CH), 131.13 (CH), 123.39 (C_q), 119.28 (CH), 112.50 (C_q), 76.03 (CH), 63.34 (CH_2), 39.76 (CH_2), 36.55 (CH_2), 22.45 (CH_3), 17.34 (CH_3). $[\alpha]_D^{20}$ = +15 (c = 1.5, CH_2Cl_2). HPLC separation (Chiralpak® IE-3, *n*-hexane/*i*-PrOH 80:20, 1.0 mL/min, detection at 273 nm): t_r (major) = 17.3 min, t_r (minor) = 21.1 min, 92.5:7.5 er.

Spectral data were consistent with data reported in the literature.[4]

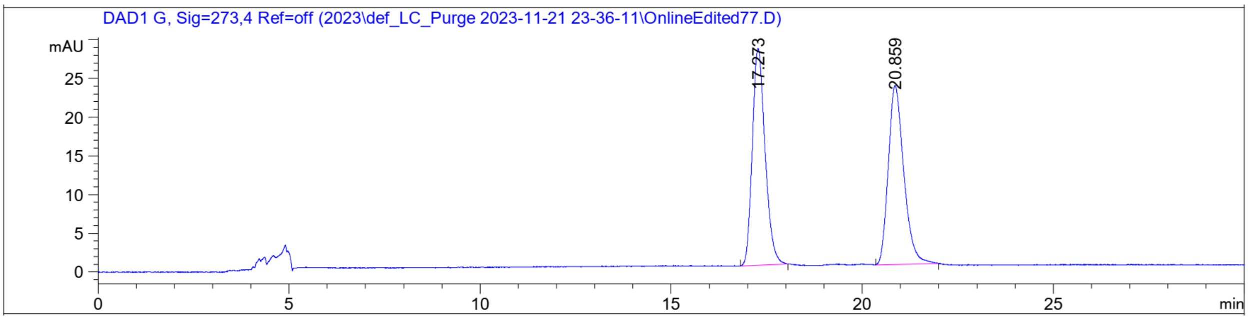

Peak #	RetTime [min]	Type	Width [min]	Area [mAU*s]	Height [mAU]	Area %
1	17.273	BB	0.3248	649.39557	27.98032	49.8845
2	20.859	BB	0.3904	652.40393	23.10802	50.1155

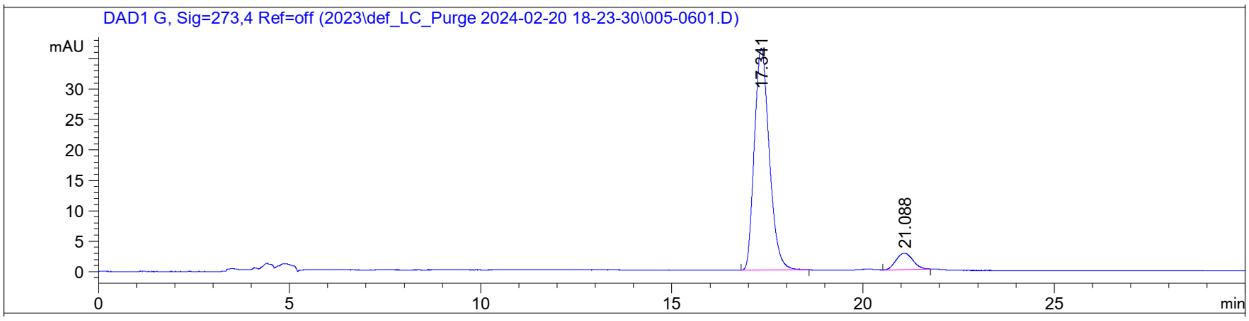

Peak #	RetTime [min]	Type	Width [min]	Area [mAU*s]	Height [mAU]	Area %
1	17.341	BB	0.4175	990.21606	36.49783	92.4320
2	21.088	BB	0.3567	81.07486	2.68150	7.5680

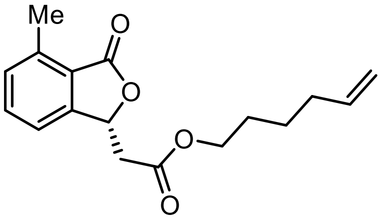

3aj

hex-5-en-1-yl (*R*)-2-(4-methyl-3-oxo-1,3-dihydroisobenzofuran-1-yl)acetate (3aj)

The general procedure was followed using benzoic acid **1a** (27.2 mg, 0.20 mmol), acrylate **2j** (92.5 mg, 0.60 mmol) at room temperature for 48 h. Purification by column chromatography on silica gel (*n*-hexane/EtOAc: 5/1) yielded **3aj** (38.1 mg, 66%) as a yellow oil. ^{1}H NMR (300 MHz, $CDCl_3$) δ 7.53 (t, *J* = 7.6 Hz, 1H), 7.31 – 7.23 (m, 2H), 5.85 – 5.67 (m, 2H), 5.06 – 4.90 (m, 2H), 4.15 (t, *J* = 6.6 Hz, 2H), 2.87 (d, *J* = 6.5 Hz, 2H), 2.68 (s, 3H), 2.13 – 2.02 (m, 2H), 1.73 – 1.58 (m, 2H), 1.49 – 1.38 (m, 2H). ^{13}C NMR (75 MHz, $CDCl_3$) δ 170.04 (C_q), 169.41 (C_q), 149.26 (C_q), 139.91 (C_q), 138.18 (CH), 133.96 (CH), 131.13 (CH), 123.41 (C_q), 119.25 (CH), 114.94 (CH_2), 76.06 (CH), 65.16 (CH_2), 39.76 (CH_2), 33.21 (CH_2), 27.92 (CH_2), 25.08 (CH_2), 17.35 (CH_3). IR (ATR): 2929, 1758, 1062, 1482, 1346, 1020, 1166, 1008, 787 cm^{-1}. MS (ESI) *m*/*z* (relative intensity): 311 (100) $[M + Na]^+$. HR-MS (ESI): *m*/*z* calcd. for $[C_{17}H_{20}O_4 + Na]^+$ 311.1254, found 311.1257. $[\alpha]_D^{20}$ = +20.5 (c = 1.2, CH_2Cl_2). HPLC separation (Chiralpak® IE-3, *n*-hexane/*i*-PrOH 80:20, 1.0 mL/min, detection at 273 nm): t_r (major) = 17.4 min, t_r (minor) = 19.7 min, 93.5:6.5 er.

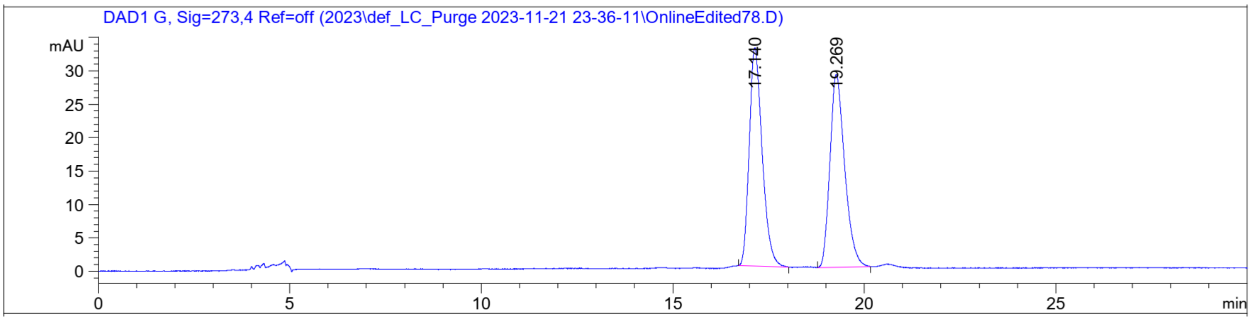

Peak #	RetTime [min]	Type	Width [min]	Area [mAU*s]	Height [mAU]	Area %
1	17.140	BB	0.3399	767.29181	32.68354	49.8373
2	19.269	BB	0.3510	772.30011	28.87590	50.1627

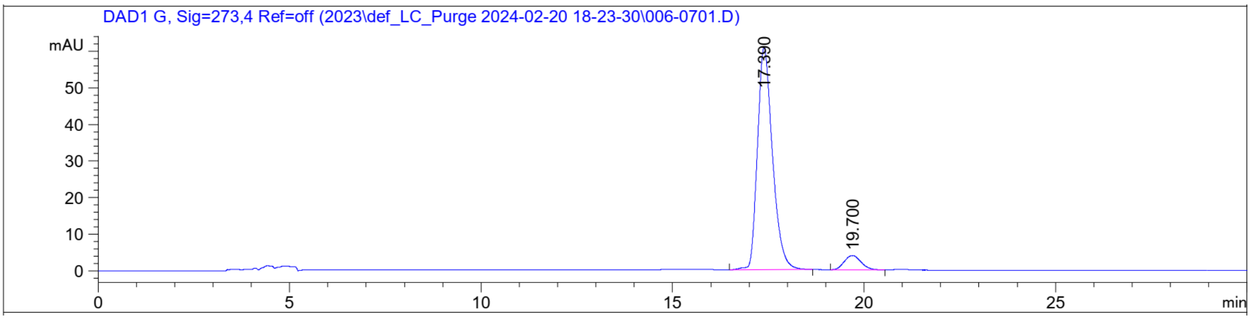

Peak #	RetTime [min]	Type	Width [min]	Area [mAU*s]	Height [mAU]	Area %
1	17.390	BB	0.4102	1661.34070	60.90958	93.3422
2	19.700	BB	0.3624	118.49756	3.92354	6.6578

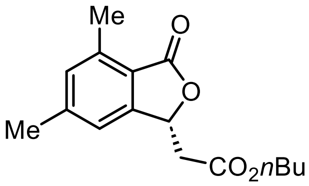

3ba

butyl (*R*)-2-(4,6-dimethyl-3-oxo-1,3-dihydroisobenzofuran-1-yl)acetate (3ba)

The general procedure was followed using benzoic acid **1b** (30 mg, 0.20 mmol), acrylate **2a** (76.9 mg, 0.60 mmol) at 40 °C for 24 h. Purification by column chromatography on silica gel (*n*-hexane/EtOAc: 10/1) yielded **3ba** (32.7 mg, 60%) as a yellow oil. ^{1}H NMR (300 MHz, $CDCl_3$) δ = 7.09 (s, 1H), 7.05 (s, 1H), 5.75 (t, J = 6.5 Hz, 1H), 4.15 (t, *J* = 6.7 Hz, 2H), 2.84 (d, *J* = 6.6 Hz, 2H), 2.63 (s, 3H), 2.42 (s, 3H), 1.61 (dq, *J* = 8.4, 6.5 Hz, 2H), 1.44 – 1.29 (m, 2H), 0.92 (t, *J* = 7.4 Hz, 3H). ^{13}C NMR (75 MHz, $CDCl_3$) δ = 170.2 (C_q), 169.6 (C_q), 150.0 (C_q), 145.2 (C_q), 139.6 (C_q), 132.34 (CH), 121.0 (C_q), 119.8 (CH), 75.9 (CH), 65.2 (CH_2), 39.9 (CH_2), 30.6 (CH_2), 22.0 (CH_3), 19.1 (CH_3), 17.3 (CH_2), 13.8 (CH_3). HPLC separation (Chiralpak® ID-3, *n*-hexane/*i*-PrOH 90:10, 1.0 mL/min, detection at 273 nm): t_r (major) = 20.3 min, t_r (minor) = 27.8 min, 86.5:13.5 er.

Spectral data were consistent with data reported in the literature.[4]

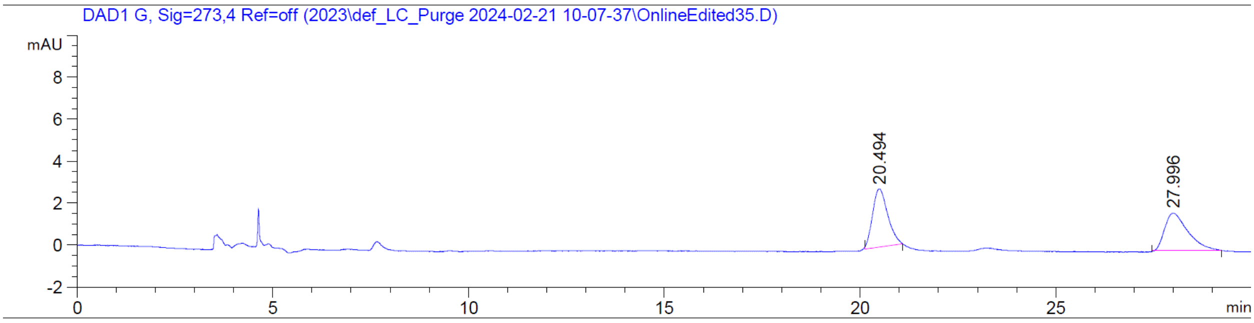

Peak #	RetTime [min]	Type	Width [min]	Area [mAU*s]	Height [mAU]	Area %
1	20.490	BB	0.4150	235.95343	7.58262	52.8921
2	28.002	BB	0.5234	210.14975	4.74736	47.1079

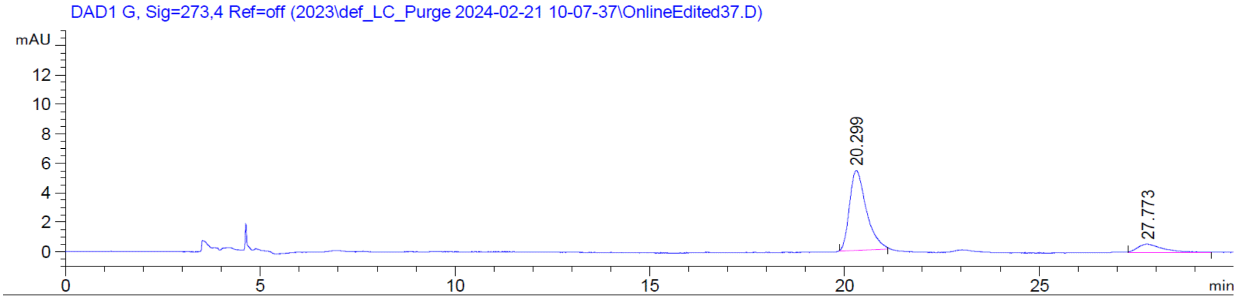

Peak #	RetTime [min]	Type	Width [min]	Area [mAU*s]	Height [mAU]	Area %
1	20.299	MM R	0.4696	162.05933	5.41110	86.4489
2	27.773	MM R	0.7545	25.40319	5.61132e-1	13.5511

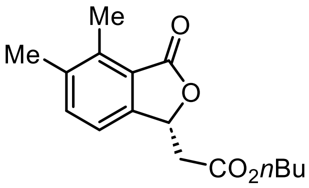

3ca

butyl (*R*)-2-(4,5-dimethyl-3-oxo-1,3-dihydroisobenzofuran-1-yl)acetate (3ca)

The general procedure was followed using benzoic acid **1c** (30 mg, 0.20 mmol), acrylate **2a** (76.9 mg, 0.60 mmol) using DCE:1,4-dioxane (3:1, 4 mL) as the solvent instead of CPME at 40 °C for 24 h. Purification by column chromatography on silica gel (*n*-hexane/EtOAc: 10/1) yielded **3ca** (50.6 mg, 92%) as a yellow oil. ^{1}H NMR (300 MHz, $CDCl_3$) δ = 7.40 (d, *J* = 7.7 Hz, 1H), 7.15 (d, *J* = 7.7 Hz, 1H), 5.74 (t, *J* = 6.5 Hz, 1H), 4.14 (t, *J* = 6.7 Hz, 2H), 2.83 (d, *J* = 6.5 Hz, 2H), 2.62 (s, 3H), 2.34 (s, 3H), 1.60 (dtd, *J* = 8.5, 7.0, 5.8 Hz, 2H), 1.45 – 1.27 (m, 2H), 0.92 (t, *J* = 7.3 Hz, 3H). ^{13}C NMR (75 MHz, $CDCl_3$) δ = 170.5 (C_q), 169.6 (C_q), 147.1 (C_q), 138.8 (C_q), 138.5 (C_q), 135.6 (CH), 123.4 (C_q), 118.7 (CH), 75.4 (CH), 65.1 (CH_2), 40.0 (CH_2), 30.6 (CH_2), 19.2 (CH_3), 19.1 (CH_2), 13.7 (CH_3), 13.3 (CH_3). HPLC separation (Chiralpak® IE-3, *n*-hexane/*i*-PrOH 80:20, 1.0 mL/min, detection at 273 nm): t_r (major) = 18.3 min, t_r (minor) = 24.2 min, 90:10 er.

Spectral data were consistent with data reported in the literature.[4]

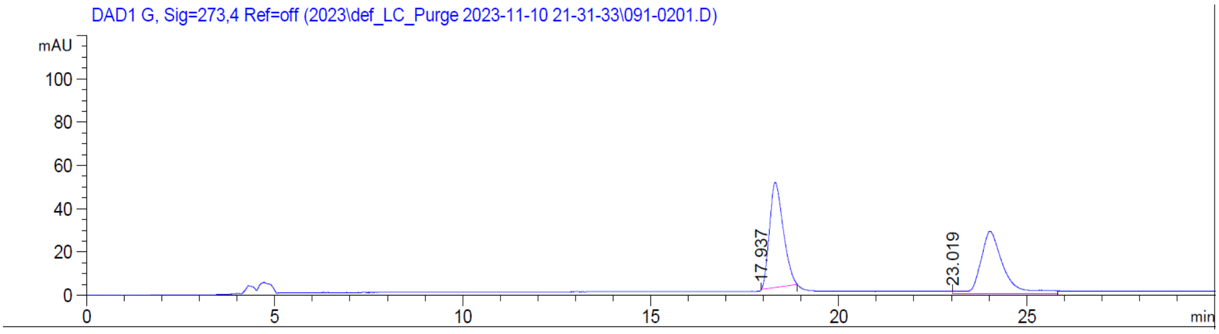

Peak #	RetTime [min]	Type	Width [min]	Area [mAU*s]	Height [mAU]	Area %
1	17.937	PP R	0.4217	1231.42041	0.00000	50.5864
2	23.019	MM R	0.6964	1202.86975	1.09789	49.4136

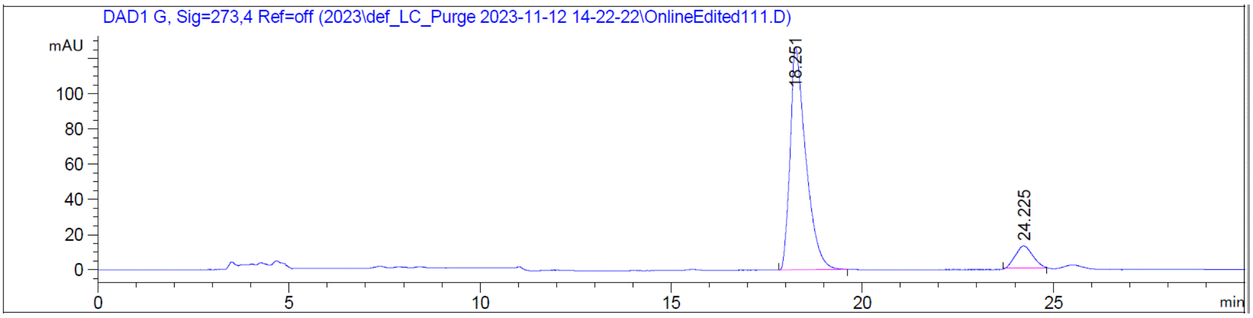

Peak #	RetTime [min]	Type	Width [min]	Area [mAU*s]	Height [mAU]	Area %
1	18.251	BB	0.4027	3619.09839	126.53008	89.7230
2	24.225	MM R	0.5378	414.53458	12.84779	10.2770

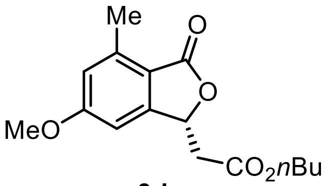

3da

butyl (*R*)-2-(6-methoxy-4-methyl-3-oxo-1,3-dihydroisobenzofuran-1-yl)acetate (3da)

The general procedure was followed using benzoic acid **1d** (33.2 mg, 0.20 mmol), acrylate **2a** (76.9 mg, 0.60 mmol) at 40 °C for 24 h. Purification by column chromatography on silica gel (*n*-hexane/EtOAc: 10/1) yielded **3da** (33.7 mg, 58%) as a yellow oil. ^{1}H NMR (300 MHz, $CDCl_3$) δ = 6.78 (d, *J* = 2.2 Hz, 1H), 6.72 (d, *J* = 2.1 Hz, 1H), 5.71 (t, *J* = 6.6 Hz, 1H), 4.14 (t, *J* = 6.7 Hz, 2H), 3.85 (s, 3H), 2.99 – 2.75 (m, 2H), 2.62 (s, 3H), 1.61 (tt, *J* = 8.5, 6.5 Hz, 2H), 1.46 – 1.27 (m, 2H), 0.92 (t, *J* = 7.3 Hz, 3H). ^{13}C NMR (75 MHz, $CDCl_3$) δ = 169.8 (C_q), 169.6 (C_q), 164.5 (C_q), 152.2 (C_q), 141.7 (C_q), 117.7 (CH), 116.1 (C_q), 103.9 (CH), 75.6 (CH), 65.2 (CH_2), 55.8 (CH_3),

39.9 (CH_2), 30.6 (CH_2), 19.1 (CH_2), 17.6 (CH_3), 13.7 (CH_3). MS (ESI) *m/z* (relative intensity): 315 (100) $[M + Na]^+$. HR-MS (ESI): *m/z* calcd. for $[C_{16}H_{20}O_5 + Na]^+$ 315.1208, found 315.1204. $[\alpha_D^{20}]$ = +36.0 (c = 0.4, CH_2Cl_2). HPLC separation (Chiralpak® IF-3, *n*-hexane/i-PrOH 80:20, 1.0 mL/min, detection at 273 nm): t_r (major) = 10.6 min, t_r (minor) = 14.1 min, 87:13 er.

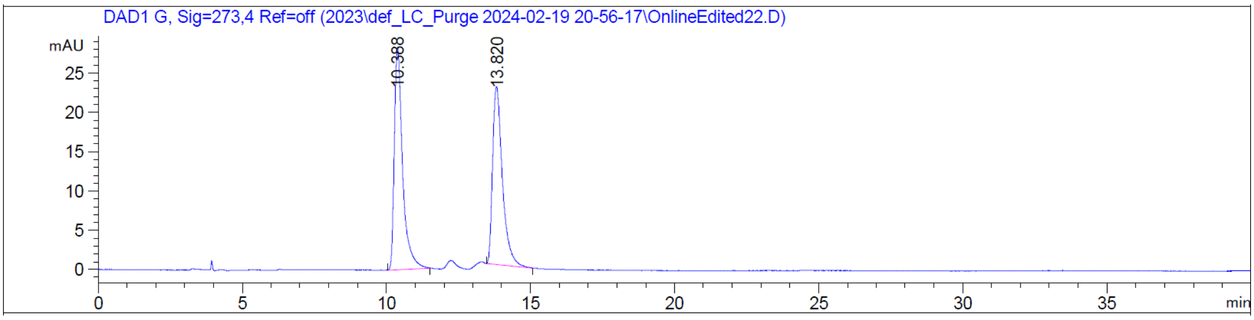

Peak #	RetTime [min]	Type	Width [min]	Area [mAU*s]	Height [mAU]	Area %
1	10.388	BB	0.2951	565.56421	28.30445	51.4476
2	13.820	BB	0.3497	533.73688	22.66706	48.5524

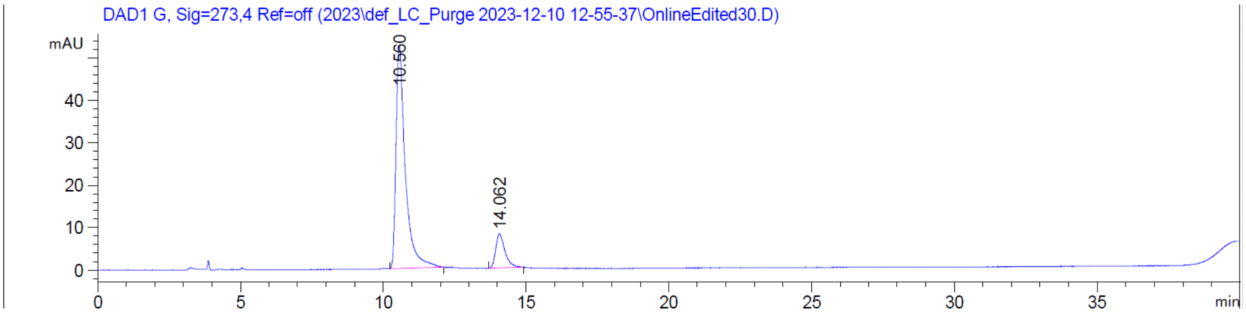

Peak #	RetTime [min]	Type	Width [min]	Area [mAU*s]	Height [mAU]	Area %
1	10.560	BB	0.3262	1186.43542	52.58155	86.7432
2	14.062	BB	0.2781	181.32083	8.04012	13.2568

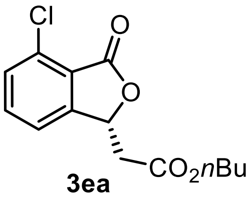

3ea

butyl (*R*)-2-(4-chloro-3-oxo-1,3-dihydroisobenzofuran-1-yl)acetate (3ea)

The general procedure was followed using benzoic acid **1e** (78.3 mg, 0.20 mmol), acrylate **2a** (76.9 mg, 0.60 mmol) using DCE:1,4-dioxane (3:1, 4 mL) as the solvent instead of CPME at 40 °C for 24 h. Purification by column chromatography on silica gel (*n*-hexane/EtOAc: 10/1) yielded **3ea**

(22.4 mg, 40%) as a yellow oil. ^{1}H NMR (300 MHz, $CDCl_3$) δ = 7.59 (t, J = 7.7 Hz, 1H), 7.50 – 7.37 (m, 2H), 5.80 (t, J = 6.4 Hz, 1H), 4.11 (t, J = 6.7 Hz, 2H), 2.94 – 2.80 (m, 2H), 1.57 (dq, J = 8.5, 6.6 Hz, 2H), 1.40 – 1.26 (m, 2H), 0.89 (t, J = 7.3 Hz, 3H). ^{13}C NMR (75 MHz, $CDCl_3$) δ = 169.1 (C_q), 166.8 (C_q), 151.2 (C_q), 135.3 (CH), 133.4 (C_q), 130.9 (CH), 122.7 (C_q), 120.6 (CH), 75.7 (CH), 65.3 (CH_2), 39.3 (CH_2), 30.5 (CH_2), 19.0 (CH_2), 13.7 (CH_3). IR (ATR): 2959, 1770, 1603, 1463, 1309, 1209, 1176, 1076, 1012, 680 cm^{-1}. MS (ESI) m/z (relative intensity): 305 (100) $[M + Na]^+$. HR-MS (ESI): m/z calcd. for $[C_{14}H_{15}ClO_4 + Na]^+$ 305.0556, found 305.0554. $[\alpha]^{20}_{D}$ = -3.3 (c = 0.3, CH_2Cl_2). HPLC separation (Chiralpak® IA-3, n-hexane/i-PrOH 90:10, 1.0 mL/min, detection at 273 nm): t_r (major) = 10.6 min, t_r (minor) = 12.4 min, 88:12 er.

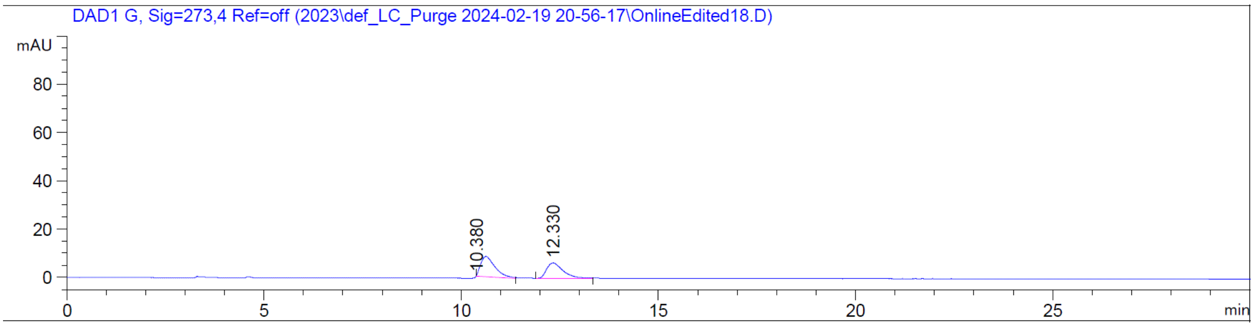

Peak #	RetTime [min]	Type	Width [min]	Area [mAU*s]	Height [mAU]	Area %
1	10.380	MM R	0.0930	213.73320	1.60985e-1	54.1199
2	12.330	MM R	0.4802	181.19231	6.28930	45.8801

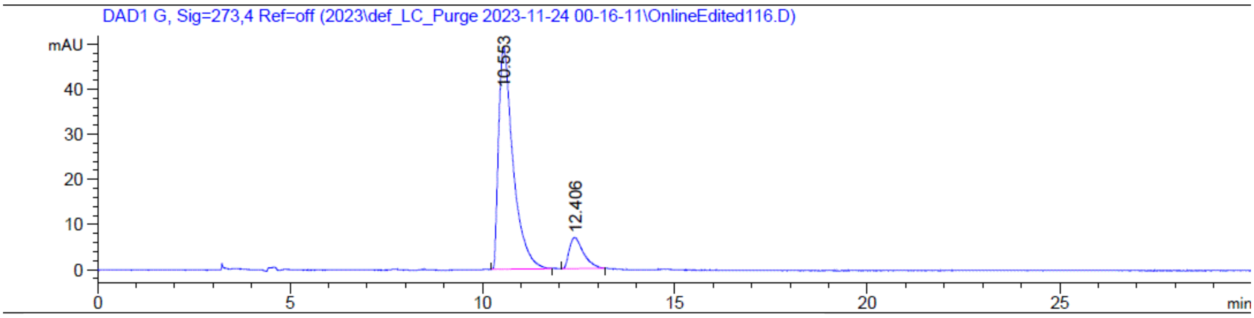

Peak #	RetTime [min]	Type	Width [min]	Area [mAU*s]	Height [mAU]	Area %
1	10.553	BB	0.3757	1287.62219	49.29489	87.9453
2	12.406	BB	0.3065	176.49533	6.87409	12.0547

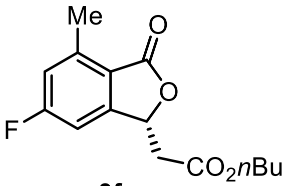

3fa

butyl (*R*)-2-(6-fluoro-4-methyl-3-oxo-1,3-dihydroisobenzofuran-1-yl)acetate (3fa)

The general procedure was followed using benzoic acid **1f** (30.8 mg, 0.20 mmol), acrylate **2a** (76.9 mg, 0.60 mmol) at 40 °C for 24 h. Purification by column chromatography on silica gel (*n*-hexane/EtOAc: 10/1) yielded **3fa** (22.4 mg, 40%) as a yellow oil. ^{1}H NMR (300 MHz, $CDCl_3$) δ = 7.09 – 6.90 (m, 2H), 5.76 (t, *J* = 6.6 Hz, 1H), 4.15 (t, *J* = 6.7 Hz, 2H), 3.05 – 2.75 (m, 2H), 2.67 (s, 3H), 1.74 – 1.52 (m, 2H), 1.36 (dp, *J* = 9.6, 7.3 Hz, 2H), 0.92 (t, *J* = 7.3 Hz, 3H). ^{13}C NMR (75 MHz, $CDCl_3$) δ = 169.36 (C_q), 169.06 (C_q), 167.9 (C_q), 164.5 (C_q), 152.2 (d, *J* = 10.6 Hz, C_q), 143.1 (d, *J* = 10.2 Hz, C_q), 118.8 (d, *J* = 23.1 Hz, CH), 107.0 (d, *J* = 24.6 Hz, CH), 75.6 (d, *J* = 3.0 Hz, CH), 65.3 (CH_2), 39.5 (CH_2), 30.6 (CH_2), 19.1 (CH_2), 17.6 (d, *J* = 1.4 Hz, CH_3), 13.7 (CH_3). ^{19}F NMR (282 MHz, $CDCl_3$) δ = -103.43. IR (ATR): 2960, 1769, 1732, 1604, 1478, 1349, 1319, 1276, 1176, 1017, 682 cm^{-1}. MS (ESI) *m/z* (relative intensity): 303 (100) $[M + Na]^+$. HR-MS (ESI): *m/z* calcd. for $[C_{15}H_{17}FO_4 + Na]^+$ 303.1008, found 303.1003. $[\alpha_D^{20}]$ = +3.0 (c = 0.5, CH_2Cl_2). HPLC separation (Chiralpak® IE-3, *n*-hexane/i-PrOH 80:20, 1.0 mL/min, detection at 273 nm): t_r (major) = 11.4 min, t_r (minor) = 13.3 min, 91:9 er.

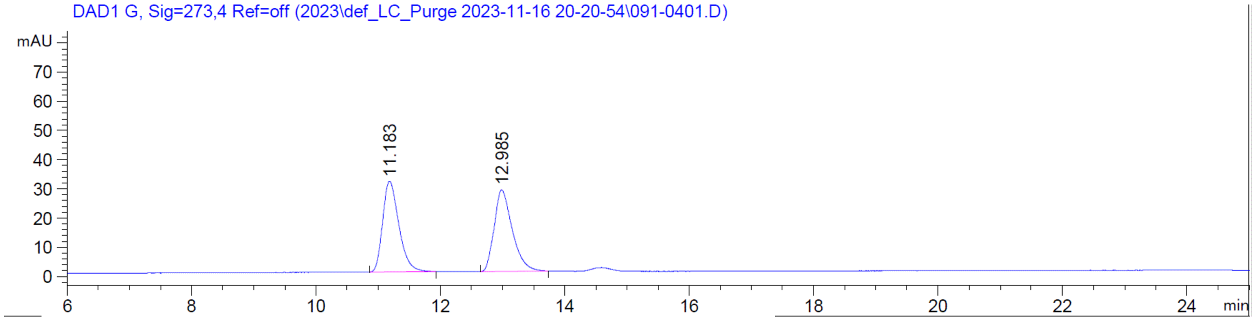

Peak #	RetTime [min]	Type	Width [min]	Area [mAU*s]	Height [mAU]	Area %
1	11.183	BB	0.2754	562.06116	30.87442	50.0404
2	12.985	BB	0.2809	561.15332	27.87517	49.9596

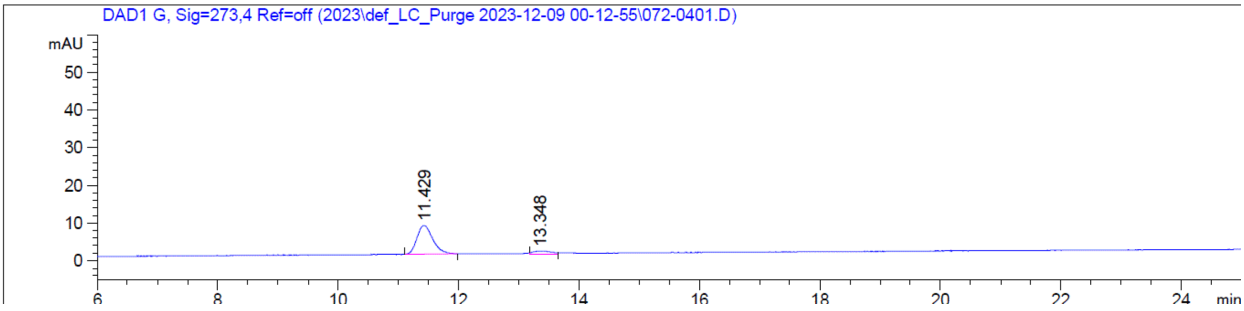

Peak #	RetTime [min]	Type	Width [min]	Area [mAU*s]	Height [mAU]	Area %
1	11.429	BB	0.2273	138.16652	7.62871	91.1723
2	13.348	MM R	0.3280	13.37792	6.79761e-1	8.8277

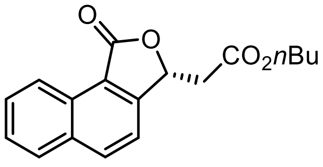

3ga

butyl (*R*)-2-(1-oxo-1,3-dihydronaphtho[1,2-c]furan-3-yl)acetate (3ga)

The general procedure was followed using benzoic acid **1g** (30.8 mg, 0.20 mmol), acrylate **2a** (76.9 mg, 0.60 mmol) using DCE:1,4-dioxane (3:1, 4 mL) as the solvent instead of CPME at 40 °C for 24 h. Purification by column chromatography on silica gel (*n*-hexane/EtOAc: 10/1) yielded **3ga** (50.1 mg, 84%) as a yellow oil. ^{1}H NMR (400 MHz, $CDCl_3$) δ = 9.01 (dd, J = 8.3, 1.1 Hz, 1H), 8.15 (d, J = 8.4 Hz, 1H), 7.98 (d, J = 8.2 Hz, 1H), 7.74 (ddd, J = 8.3, 7.0, 1.3 Hz, 1H), 7.65 (ddd, J = 8.2, 7.0, 1.3 Hz, 1H), 7.53 (d, J = 8.4 Hz, 1H), 5.96 (t, J = 6.5 Hz, 1H), 4.18 (t, J = 6.7 Hz, 2H), 2.96 (d, J = 6.6 Hz, 2H), 1.68 – 1.58 (m, 2H), 1.45 – 1.32 (m, 2H), 0.92 (t, J = 7.4 Hz, 3H). ^{13}C NMR (101 MHz, $CDCl_3$) δ = 170.3 (C_q), 169.5 (C_q), 150.5 (C_q), 135.9 (CH), 133.6 (C_q), 129.3 (CH), 128.6 (C_q), 127.6 (CH), 123.7 (CH), 120.4 (C_q), 118.6 (CH), 76.5 (CH), 65.4 (CH_2), 39.5 (CH_2), 30.6 (CH_2), 19.2 (CH_2), 13.8 (CH_3). HPLC separation (Chiralpak® IA-3, *n*-hexane/*i*-PrOH 95:5, 1.0 mL/min, detection at 273 nm): t_r (major) = 14.7 min, t_r (minor) = 16.1 min, 90:10 er.

Spectral data were consistent with data reported in the literature.[4]

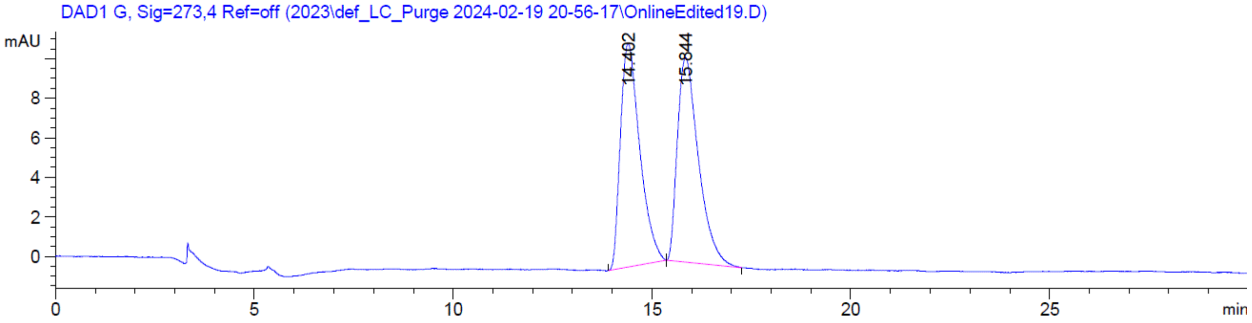

Peak #	RetTime [min]	Type	Width [min]	Area [mAU*s]	Height [mAU]	Area %
1	14.402	BB	0.4704	382.60248	11.31759	50.1046
2	15.844	BB	0.4838	381.00500	10.34175	49.8954

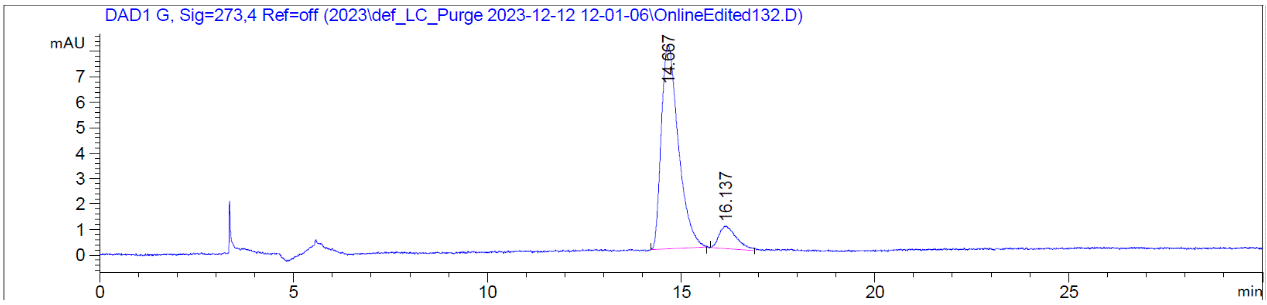

Peak #	RetTime [min]	Type	Width [min]	Area [mAU*s]	Height [mAU]	Area %
1	14.667	BB	0.3628	245.29828	8.01981	89.8943
2	16.137	MM R	0.5194	27.57599	8.84906e-1	10.1057

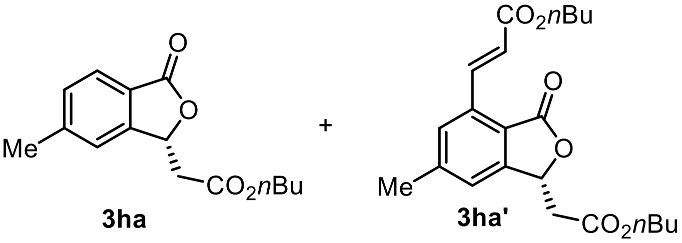

The general procedure was followed using benzoic acid **1h** (27.2 mg, 0.20 mmol), acrylate **2a** (76.9 mg, 0.60 mmol) using DCE:1,4-dioxane (3:1, 4 mL) as the solvent instead of CPME at 40 °C for 24 h. Purification by column chromatography on silica gel (*n*-hexane/EtOAc: 10/1) yielded **3ha** (26.7 mg, 51%) as a yellow solid and **3ha'** (20.2 mg, 26%) as a yellow oil.

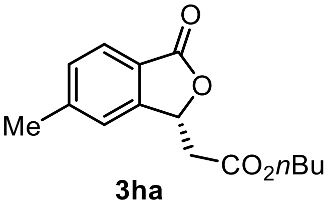

butyl (*R*)-2-(6-methyl-3-oxo-1,3-dihydroisobenzofuran-1-yl)acetate (3ha)

^{1}H NMR (300 MHz, $CDCl_3$) δ = 7.78 (d, J = 7.8 Hz, 1H), 7.35 (ddt, J = 7.8, 1.3, 0.6 Hz, 1H), 7.29 – 7.27 (m, 1H), 5.83 (t, J = 6.6 Hz, 1H), 4.17 (t, J = 6.7 Hz, 2H), 2.97 – 2.80 (m, 2H), 2.49 (s, 3H), 1.67 – 1.57 (m, 2H), 1.42 – 1.33 (m, 2H), 0.94 (t, J = 7.3 Hz, 3H). ^{13}C NMR (75 MHz, $CDCl_3$) δ = 170.0 (C_q), 169.6 (C_q), 149.5 (C_q), 145.7 (C_q), 130.8 (CH), 125.7 (CH), 123.5 (C_q), 122.5 (CH), 76.8 (CH), 65.3 (CH_2), 39.7 (CH_2), 30.6 (CH_2), 22.2 (CH_3), 19.2 (CH_2), 13.8 (CH_3). HPLC separation (Chiralpak® IA-3, *n*-hexane/*i*-PrOH 80:20 1.0 mL/min, detection at 250 nm): t_r (major) = 6.8 min, t_r (minor) = 8.6 min, 86:14 er.

Spectral data were consistent with data reported in the literature.[4]

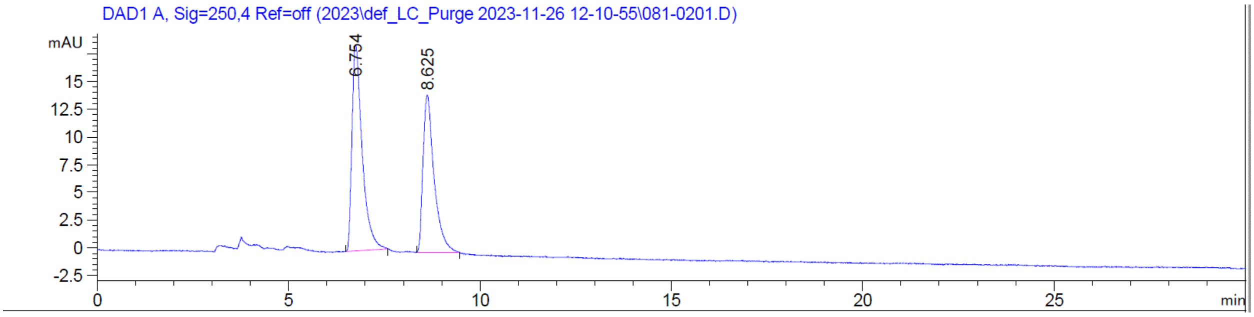

Peak #	RetTime [min]	Type	Width [min]	Area [mAU*s]	Height [mAU]	Area %
1	6.754	BB	0.2622	344.22607	18.57739	54.5787
2	8.625	BB	0.2814	286.47009	14.19989	45.4213

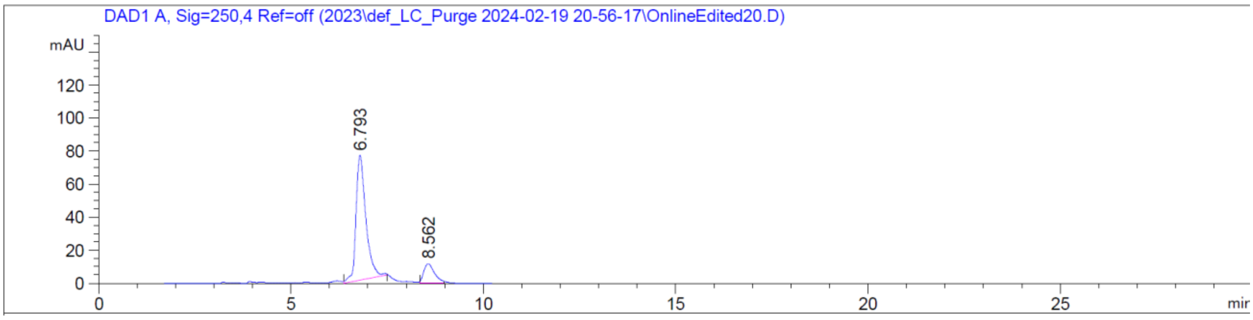

Peak #	RetTime [min]	Type	Width [min]	Area [mAU*s]	Height [mAU]	Area %
1	6.793	MM R	0.2976	1349.80444	75.60114	86.1915
2	8.562	MM R	0.3137	216.24767	11.48977	13.8085

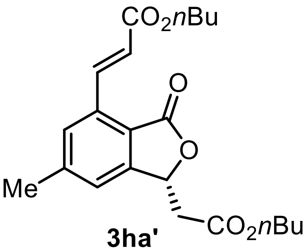

3ha'

butyl (*R*,*E*)-3-(1-(2-butoxy-2-oxoethyl)-6-methyl-3-oxo-1,3-dihydroisobenzofuran-4-yl) acrylate (3ha')

^{1}H NMR (300 MHz, $CDCl_3$) δ = 8.65 (d, *J* = 16.2 Hz, 1H), 7.57 (d, *J* = 1.4 Hz, 1H), 7.26 (s, 1H), 6.59 (d, *J* = 16.2 Hz, 1H), 5.79 (t, *J* = 6.5 Hz, 1H), 4.23 (t, *J* = 6.7 Hz, 2H), 4.16 (t, *J* = 6.7 Hz, 2H), 2.97 – 2.79 (m, 2H), 2.49 (s, 3H), 1.72 – 1.56 (m, 4H), 1.47 – 1.31 (m, 4H), 0.98 – 0.92 (m, 6H). ^{13}C NMR (101 MHz, $CDCl_3$) δ = 169.4 (C_q), 169.0 (C_q), 166.4 (C_q), 150.4 (C_q), 145.6 (C_q), 137.6 (CH), 134.8 (C_q), 127.8 (CH), 123.6 (CH), 122.9 (CH), 120.9 (C_q), 75.9 (CH), 65.3 (CH_2), 64.8 (CH_2), 39.6 (CH_2), 30.8 (CH_2), 30.6 (CH_2), 22.2 (CH_3), 19.3 (CH_2), 19.2 (CH_2), 13.9 (CH_3),

13.8 (CH_3). HPLC separation (Chiralpak® IA-3, *n*-hexane/*i*-PrOH 90:10, 1.0 mL/min, detection at 220 nm): t_r (major) = 12.1 min, t_r (minor) = 13.7 min, 89:11 er.

Spectral data were consistent with data reported in the literature.[8]

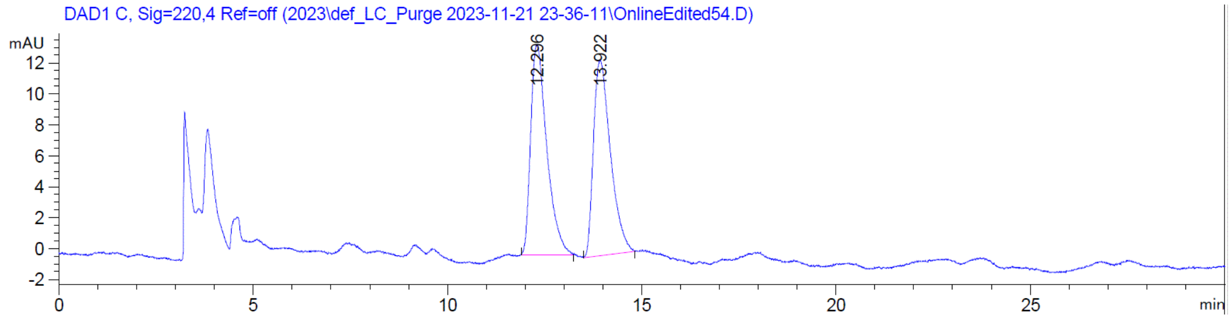

Peak #	RetTime [min]	Type	Width [min]	Area [mAU*s]	Height [mAU]	Area %
1	12.296	BV R	0.3414	391.10355	13.56489	50.4954
2	13.922	BB	0.3593	383.42981	12.66062	49.5046

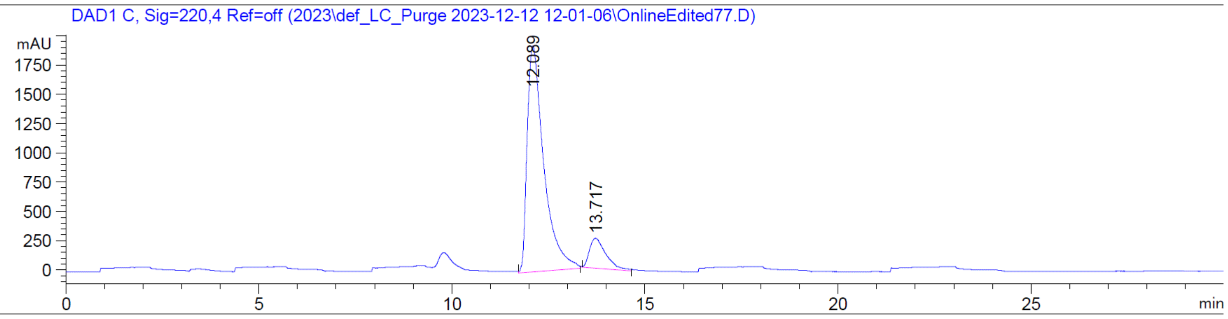

Peak #	RetTime [min]	Type	Width [min]	Area [mAU*s]	Height [mAU]	Area %
1	12.089	MM R	0.4119	5.80308e4	1929.79675	88.5544
2	13.717	MM R	0.4902	7500.46387	255.01825	11.4456

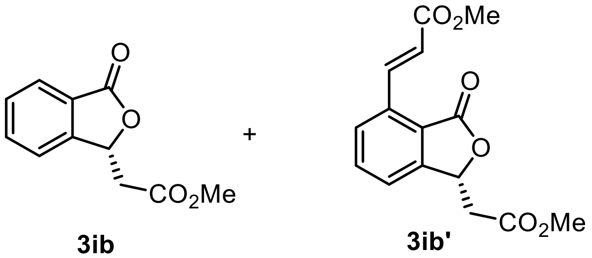

3ib **3ib'**

3ib + **3ib'**: 72% (mono:di = 2:1)

The general procedure was followed using benzoic acid **1i** (24.4 mg, 0.20 mmol), acrylate **2b** (51.7 mg, 0.60 mmol) at 40 °C for 24 h. Purification by column chromatography on silica gel (*n*-hexane/EtOAc: 3/1) yielded **3ib** (20.2 mg, 49%) as a yellow solid and **3ib'** (13.4 mg, 23%) as a yellow oil.

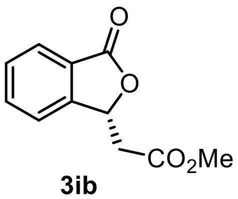

3ib

methyl (*R*)-2-(3-oxo-1,3-dihydroisobenzofuran-1-yl)acetate (3ib)

^{1}H NMR (300 MHz, $CDCl_3$) δ 7.91 (dt, J = 7.5, 1.0 Hz, 1H), 7.68 (td, J = 7.5, 1.1 Hz, 1H), 7.55 (tt, J = 7.5, 0.9 Hz, 1H), 7.50 (dq, J = 7.7, 0.9 Hz, 1H), 5.88 (dd, J = 7.2, 6.1 Hz, 1H), 3.76 (s, 3H), 2.98 – 2.83 (m, 2H). ^{13}C NMR (75 MHz, $CDCl_3$) δ 169.84 (C_q), 169.74 (C_q), 148.71 (C_q), 134.34 (CH), 129.63 (CH), 125.93 (C_q), 125.91 (CH), 122.09 (CH), 76.94 (CH), 52.26 (CH_3), 39.40 (CH_2). HPLC separation (Chiralpak® IA-3, *n*-hexane/*i*-PrOH 80:20, 1.0 mL/min, detection at 273 nm): t_r (major) = 8.2 min, t_r (minor) = 13.1 min, 89:11 er.

Spectral data were consistent with data reported in the literature.[6]

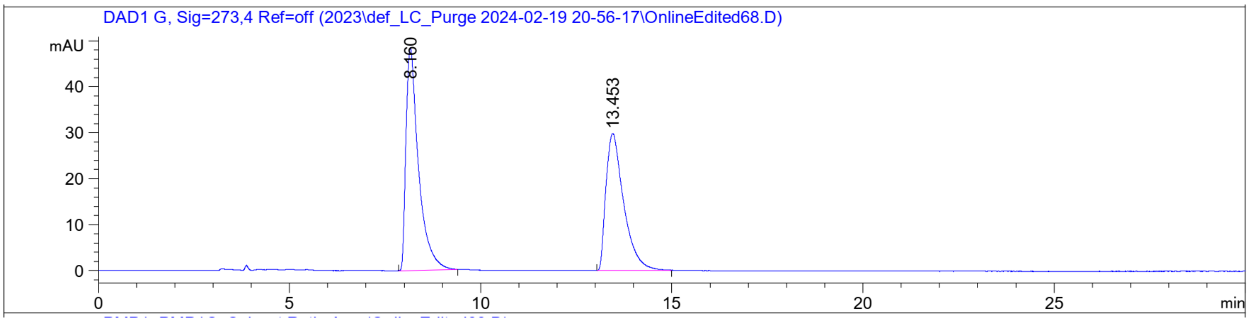

Peak #	RetTime [min]	Type	Width [min]	Area [mAU*s]	Height [mAU]	Area %
1	8.160	BB	0.3383	1101.95850	48.46227	53.4437
2	13.453	BB	0.4809	959.94843	29.79523	46.5563

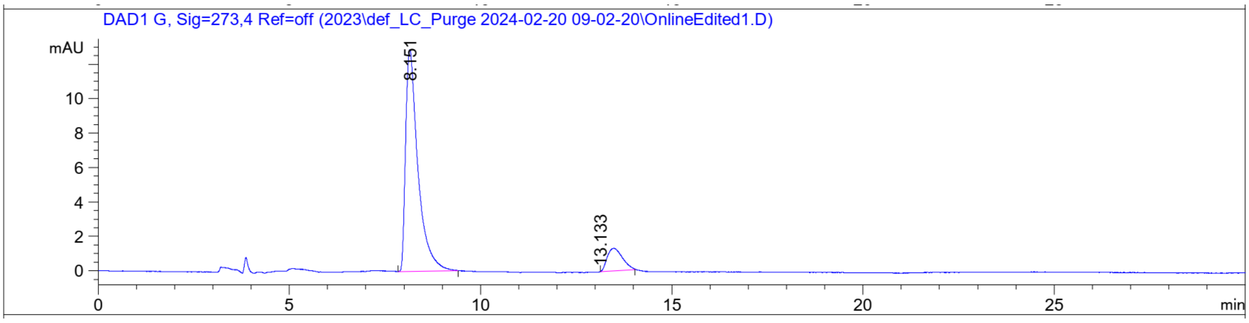

Peak #	RetTime [min]	Type	Width [min]	Area [mAU*s]	Height [mAU]	Area %
1	8.151	BB	0.3295	288.62292	12.87908	88.7818
2	13.133	MM R	0.4611	36.46962	7.85971e-3	11.2182

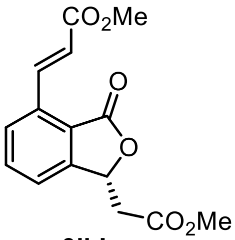

3ib'

methyl (*R,E*)-3-(1-(2-methoxy-2-oxoethyl)-3-oxo-1,3-dihydroisobenzofuran-4-yl)acrylate (3ib')

^{1}H NMR (300 MHz, $CDCl_3$) δ 8.70 (d, J = 16.2 Hz, 1H), 7.77 (dq, J = 7.8, 0.8 Hz, 1H), 7.67 (td, J = 7.7, 0.6 Hz, 1H), 7.49 (dt, J = 7.5, 0.9 Hz, 1H), 6.60 (d, J = 16.2 Hz, 1H), 5.86 (tt, J = 6.9, 0.7 Hz, 1H), 3.83 (s, 3H), 3.76 (s, 3H), 3.03 – 2.83 (m, 2H). ^{13}C NMR (75 MHz, $CDCl_3$) δ 169.62 (C_q), 168.85 (C_q), 166.53 (C_q), 149.67 (C_q), 137.49 (CH), 135.02 (C_q), 134.32 (CH), 126.74 (CH), 123.17 (C_q), 123.09 (CH), 122.67 (CH), 76.01 (CH), 52.30 (CH_3), 51.99 (CH_3), 39.30 (CH_2). HPLC separation (Chiralpak® ID-3, *n*-hexane/*i*-PrOH 80:20, 1.0 mL/min, detection at 250 nm): t_r (major) = 14.4 min, t_r (minor) = 18.4 min, 88:12 er.

Spectral data were consistent with data reported in the literature.[7]

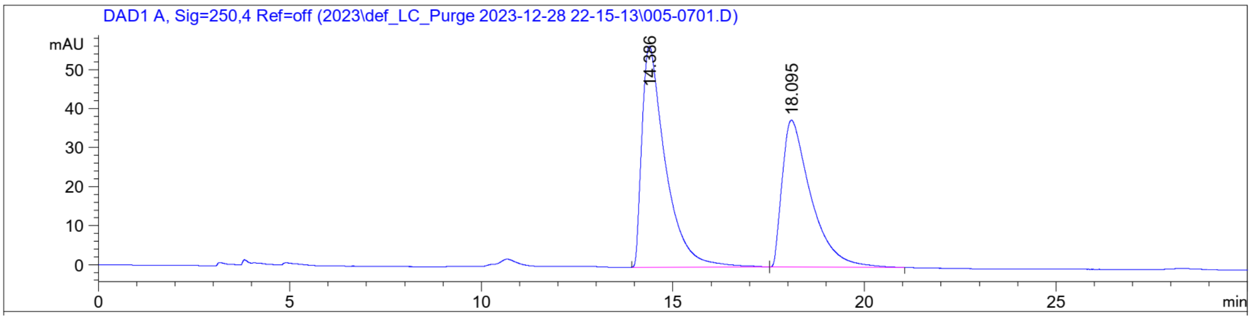

Peak #	RetTime [min]	Type	Width [min]	Area [mAU*s]	Height [mAU]	Area %
1	14.386	BB	0.6226	2421.67627	56.66294	55.1761
2	18.095	BB	0.7199	1967.31616	37.69539	44.8239

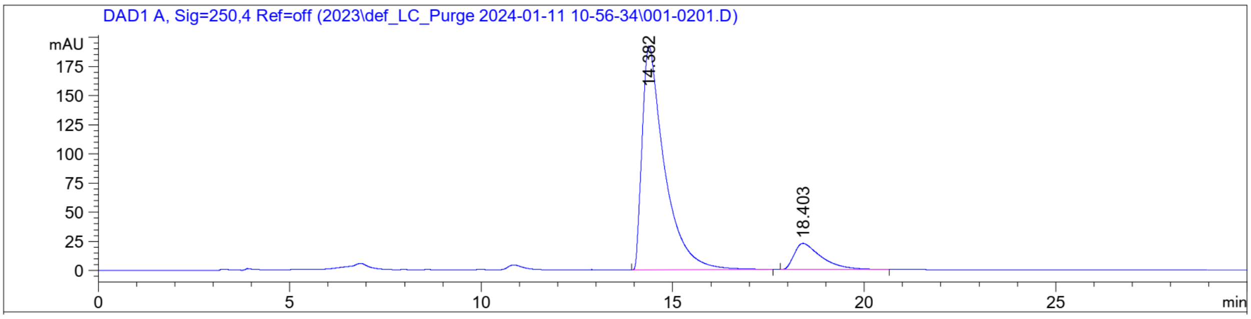

Peak #	RetTime [min]	Type	Width [min]	Area [mAU*s]	Height [mAU]	Area %
1	14.382	BB	0.5792	7780.69043	191.82268	87.6975
2	18.403	BB	0.6533	1091.49817	22.32706	12.3025

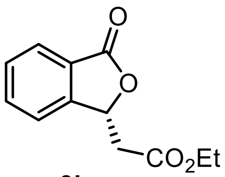

3ic

ethyl (*R*)-2-(3-oxo-1,3-dihydroisobenzofuran-1-yl)acetate (3ic)

The general procedure was followed using benzoic acid **1i** (24.4 mg, 0.20 mmol), acrylate **2c** (60.0 mg, 0.60 mmol) at 40 °C for 24 h. Purification by column chromatography on silica gel (*n*-hexane/EtOAc: 3/1) yielded **3ic** (18.0 mg, 41%) as a yellow oil. ^{1}H NMR (300 MHz, $CDCl_3$) δ 7.91 (dt, *J* = 7.6, 1.0 Hz, 1H), 7.68 (td, *J* = 7.5, 1.1 Hz, 1H), 7.56 (tt, *J* = 7.6, 0.8 Hz, 1H), 7.50 (dq, *J* = 7.6, 0.9 Hz, 1H), 5.89 (t, *J* = 6.6 Hz, 1H), 4.22 (q, *J* = 7.2 Hz, 2H), 2.98 – 2.82 (m, 2H), 1.27 (t, *J* = 7.2 Hz, 3H). ^{13}C NMR (75 MHz, $CDCl_3$) δ 169.88 (C_q), 169.25 (C_q), 148.80 (C_q), 134.28 (CH), 129.56 (CH), 125.98 (C_q), 125.86 (CH), 122.09 (CH), 77.00 (CH), 61.30 (CH_2), 39.59 (CH_2), 14.13 (CH_3). $[\alpha_D^{23}]$ = +7.6 (c = 0.92, $CHCl_3$). The absolute configuration of the product was assigned as *R* by comparing their optical rotation with the literature.[6] HPLC separation (Chiralpak® IA-3, *n*-hexane/*i*-PrOH 80:20, 1.0 mL/min, detection at 273 nm): t_r (major) = 7.3 min, t_r (minor) = 10.1 min, 90.5:9.5 er.

Spectral data were consistent with data reported in the literature.[6]

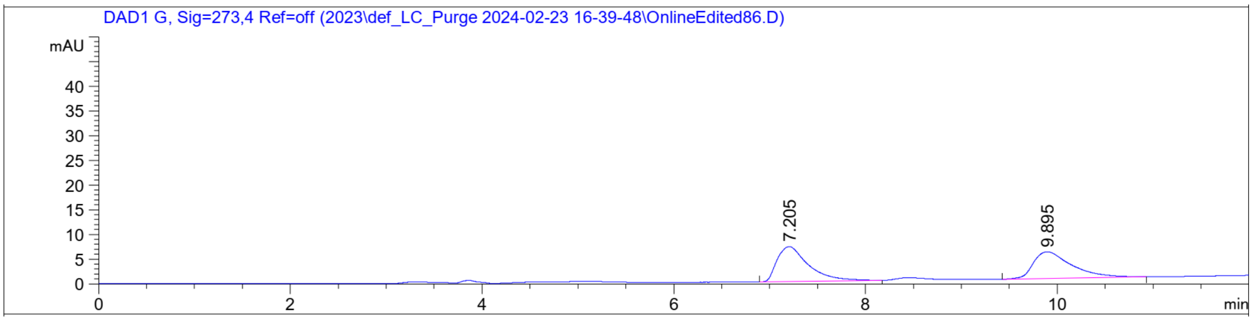

Peak #	RetTime [min]	Type	Width [min]	Area [mAU*s]	Height [mAU]	Area %
1	7.205	BB	0.3361	159.74655	7.05622	51.4897
2	9.895	BB	0.3570	150.50323	5.40270	48.5103

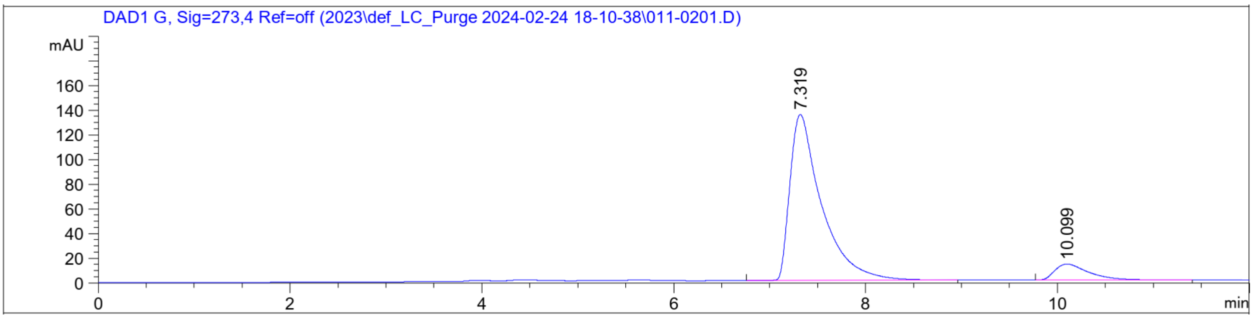

Peak #	RetTime [min]	Type	Width [min]	Area [mAU*s]	Height [mAU]	Area %
1	7.319	BB	0.3328	3091.88330	134.67262	90.5265
2	10.099	BB	0.3650	323.56409	13.01902	9.4735

4. Key Mechanistic Findings

4.1 Evidence of *oxa*-Michael Addition Reaction for the Alkenylated Intermediate

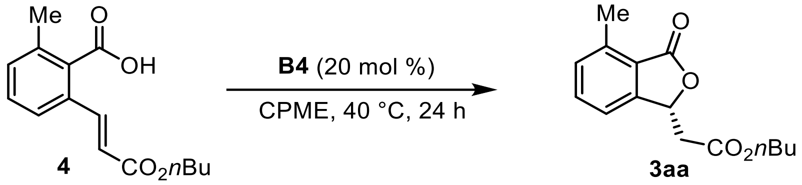

To a 10 mL vial was added alkenylated intermediate **4** (26.2 mg, 0.10 mmol), **B4** (6.2 mg, 0.02 mmol, 20 mol %), and CPME (2.0 mL). The mixture was stirred at 40 °C for 24 h. The resulting mixture was purified by column chromatography on silica gel to afford the product **3aa** (24.3 mg, 93% yield, 92:8 er).

4.2 Control experiments for organocatalyst without hydroxyl group

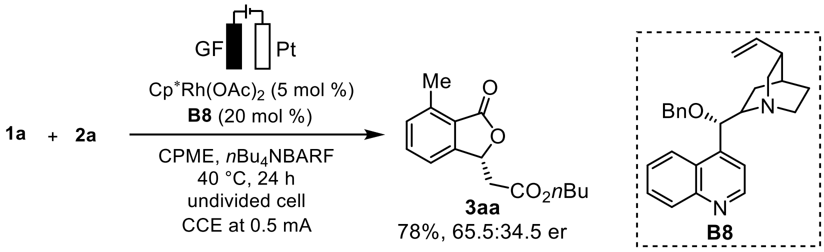

Benzoic acid **1a** (0.20 mmol), acrylate **2a** (0.60 mmol), **B8** (15.4 mg, 0.04 mmol, 20 mol %), nBu$_4$NBARF (110.0 mg, 0.20 mmol), Cp*Rh(OAc)$_2$ (3.7 mg, 0.01 mmol, 5 mol %) and CPME (4.0 mL) were placed in a 8 mL cell. Electrocatalysis was performed at 40 °C with a constant current of 0.5 mA for 24 h. The resulting mixture was purified by column chromatography on silica gel to afford the product **3aa** (40.1 mg, 78% yield, 65.5:34.5 er).

4.3 KIE Studies under Constant Current Electrolysis Conditions

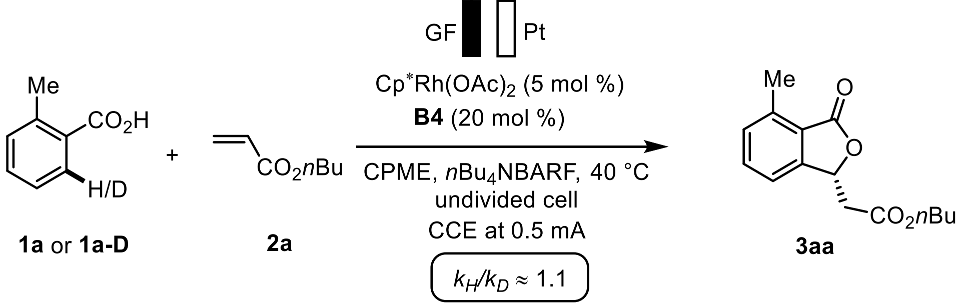

Two parallel reactions of **1a** and **1q**-D with **2a** were performed to determine the KIE by comparison

of the initial reaction rates. The electrocatalysis was carried out in an undivided cell, with a graphite felt (GF) anode (25 mm × 10 mm × 6.0 mm) and a platinum cathode (25 mm × 10 mm × 0.125 mm). Benzoic acid **1a** or **1a-D** (0.20 mmol), acrylate **2a** (0.60 mmol), triphenylmethane (48.8 mg, 0.20 mmol), **B4** (12.4 mg, 0.04 mmol, 20 mol %), *n*Bu_4NBARF (110.0 mg, 0.20 mmol), Cp*Rh$(OAc)_2$ (3.7 mg, 0.01 mmol, 5 mol %) and CPME (4.0 mL) were placed in a 8 mL cell. Electrocatalysis was performed at 40 °C with a constant current of 0.5 mA. Aliquots (100 μL) were periodically removed to provide the following conversions as determined by ^{1}H-NMR. The determined conversions of **3aa** were plotted and a linear fit resulted in a KIE value of $k_H/k_D \approx 1.1$.

Table S2: Conversion-time table.

t / min	0	4	8	12	16	20
**3aa (from 1a)** / %	0	4.9	9.4	12.2	15.1	18.3
**3aa (from 1a-D)** / %	0	4.4	8.3	10.4	13.5	16.7

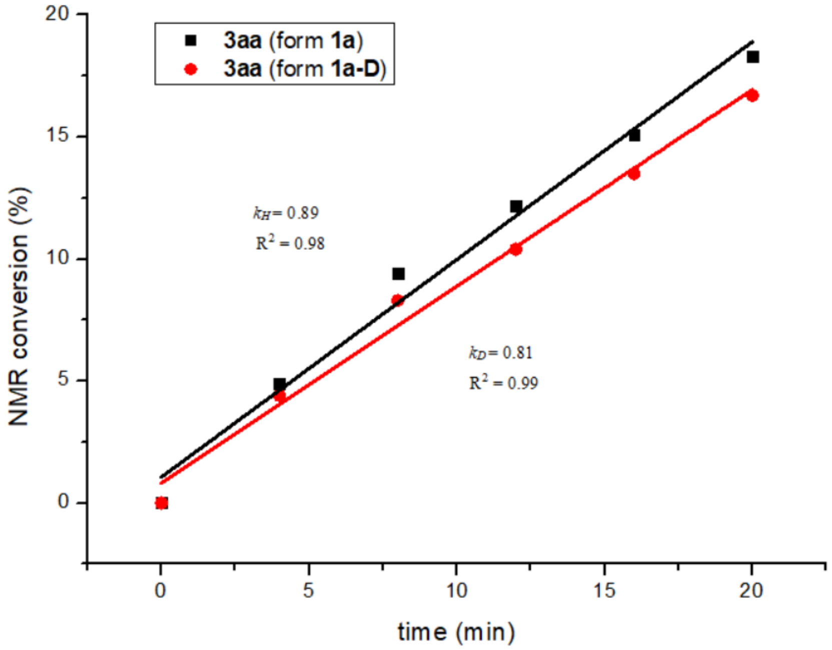

4.4 KIE Studies under Constant Potentials Electrolysis Conditions

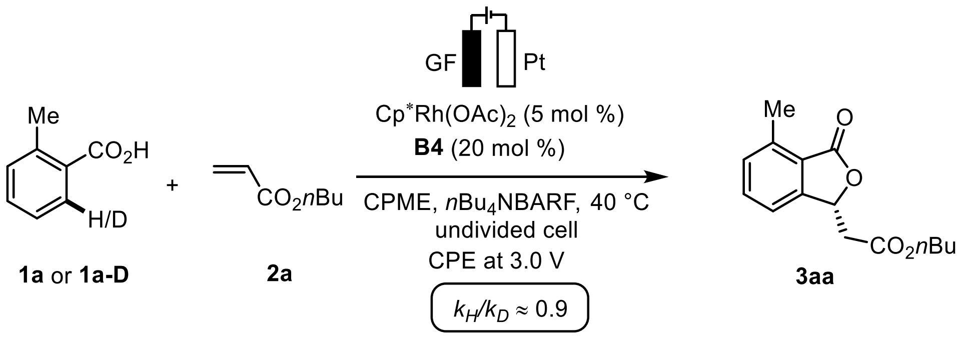

Two parallel reactions of **1a** and **1q**-D with **2a** were performed to determine the KIE by comparison of the initial reaction rates. The electrocatalysis was carried out in an undivided cell, with a graphite felt (GF) anode (25 mm × 10 mm × 6.0 mm) and a platinum cathode (25 mm × 10 mm × 0.125 mm). Benzoic acid **1a** or **1a-D** (0.20 mmol), acrylate **2a** (0.60 mmol), triphenylmethane (48.8 mg, 0.20 mmol), **B4** (12.4 mg, 0.04 mmol, 20 mol %), *n*Bu_4NBARF (110.0 mg, 0.20 mmol), Cp*Rh$(OAc)_2$ (3.7 mg, 0.01 mmol, 5 mol %) and CPME (4.0 mL) were placed in a 8 mL cell. Electrocatalysis was performed at 40 °C with a constant potential of 3.0 V. Aliquots (100 μL) were periodically removed to provide the following conversions as determined by ^{1}H-NMR. The determined conversions of **3aa** were plotted and a linear fit resulted in a KIE value of $k_H/k_D \approx 0.9$.

Table S3: Conversion-time table.

t / min	0	4	8	12	16	20
**3aa (from 1a)** / %	0	6.3	9.9	12.3	16.0	18.0
**3aa (from 1a-D)** / %	0	5.6	10.5	13.1	17.2	19.0

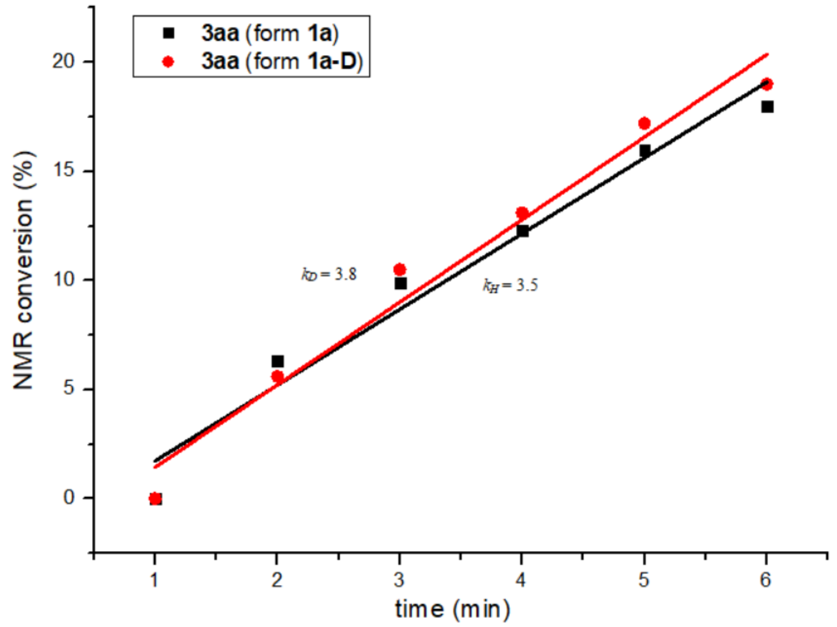

4.5 Competition Experiments

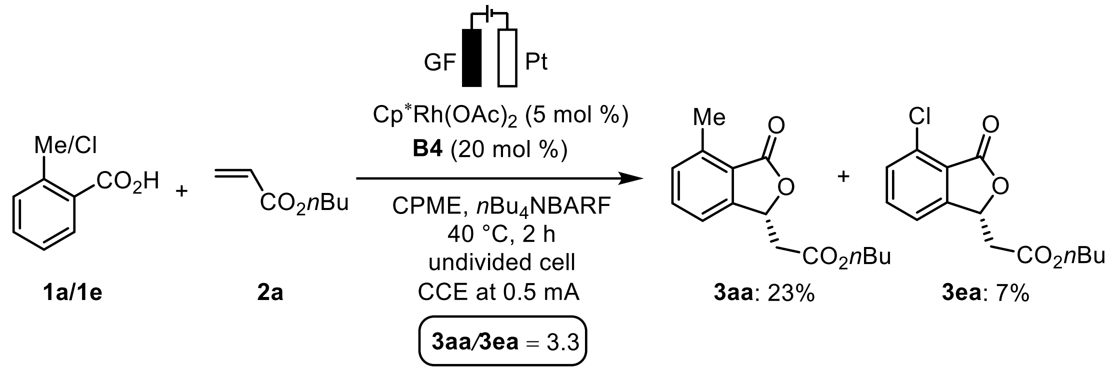

The electrocatalysis was carried out in an undivided cell, with a graphite felt (GF) anode (25 mm × 10 mm × 6.0 mm) and a platinum cathode (25 mm × 10 mm × 0.125 mm). Benzoic acid **1a** (13.6 mg, 0.10 mmol), **1e** (15.7 mg, 0.10 mmol), acrylate **2a** (76.8 mg, 0.60 mmol), **B4** (12.4 mg, 0.04 mmol, 20 mol %), *n*Bu_4NBARF (110.0 mg, 0.20 mmol), Cp*Rh$(OAc)_2$ (3.7 mg, 0.01 mmol, 5 mol %) and CPME (4.0 mL) were placed in a 8 mL cell. Electrocatalysis was performed at 40 °C with a constant current of 0.5 mA maintained for 2 h. Then, the reaction mixture was diluted with EtOAc (2.0 mL). The platinum cathode and the graphite felt anode were washed with EtOAc. Evaporation of the solvent and subsequent column chromatography on silica gel afforded **3aa** (6.1 mg, 23% yield) and **3ae** (2.0 mg, 7% yield).

5. An Alternative Catalytic Cycle

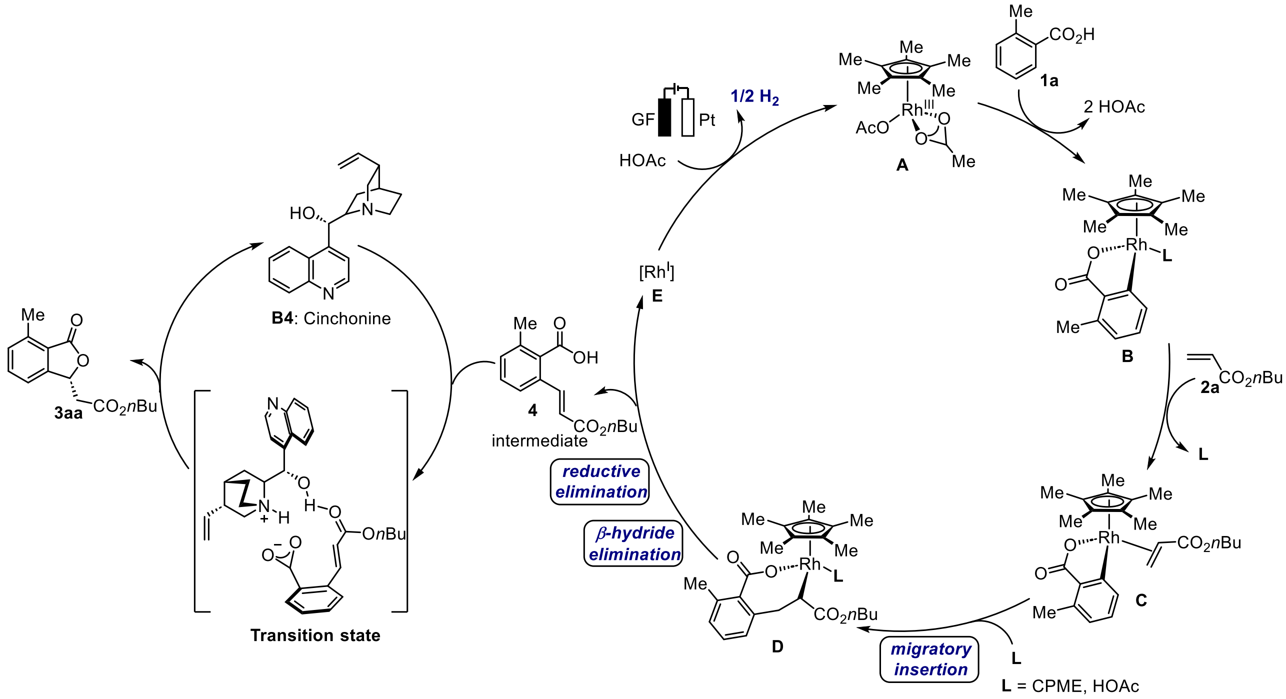

The mechanism commences with a facile C−H activation by carboxylate assistance, which forms rhodacycle **B**. Thereafter, coordination followed by migratory insertion of the acrylate takes place, which enables the formation of seven-membered intermediate **D**. Then, β-hydride elimination and reductive elimination deliver the rhodium(I) complex **E** and the intermediate **4**. Finally, the anodic oxidation regenerates the active catalytic species rhodium(III) complex **A**, while the intermediate **4** undergoes enantioselective *oxa*-Michael addition in the presence of **B4** to afford the chiral product **3aa** through the transition state.

6. Product Transformation

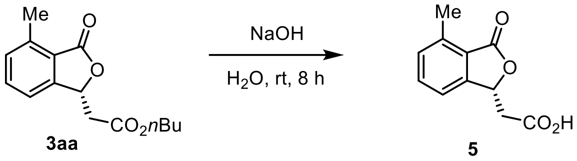

A solution of **3aa** (26.2 mg, 0.10 mmol) in 2 M NaOH (3 mL) was stirred at rt for 8 h. The reaction mixture was acidifed to pH = 2-3 with l M HCl and extracted with ethyl acetate. The organic layer was dried over magnesium sulfate and concentrated under reduced pressure. The residue was purified by flash chromatography using dichloromethane/methanol (5:1) as eluent to give the compound **5** as a brown solid (15.6 mg, 76%). ^{1}H NMR (300 MHz, Chloroform-*d*) δ 7.56 (t, *J* = 7.6 Hz, 1H), 7.34 (d, *J* = 7.1 Hz, 2H), 5.84 (t, *J* = 6.7 Hz, 1H), 2.93 (dt, *J* = 14.4, 6.1 Hz, 2H), 2.70 (s, 3H). ^{13}C NMR (75 MHz, Chloroform-*d*) δ 174.84 (C_q), 170.14 (C_q), 149.10 (C_q), 140.02 (C_q), 134.14 (CH), 131.25 (CH), 123.30 (C_q), 119.35 (CH), 76.06 (CH), 39.97 (CH_2), 17.37 (CH_3).

Spectral data were consistent with data reported in the literature.[9]

7. References

1. Feldman, P. L.; James, M. K.; Brackeen, M. F.; Bilotta, J. M.; Schuster, S. V.; Lahey, A. P.; Lutz, M. W.; Johnson, M. R.; Leighton, H. J. Design, Synthesis, and Pharmacological Evaluation of Ultrashort-to Long-Acting Opioid Analgesics. *J. Med. Chem.* **1991**, *34*, 2202-2208.

2. Liu, D.; Yang, K.; Fang, D.; Li, S.-J.; Lan, Y.; Chen, Y. Formyl Radical Generation from *α*-Chloro *N*-Methoxyphthalimides Enables Selective Aldehyde Synthesis. *Angew. Chem., Int. Ed.* **2023**, *62*, e202213686.

3. D'Annibale, A.; Ciaralli, L.; Bassetti, M.; Pasquini, C. Synthesis of Alkyl-Substituted Six-Membered Lactones through Ring-Closing Metathesis of Homoallyl Acrylates. An Easy Route to Pyran-2-ones, Constituents of Tobacco Flavor. *J. Org. Chem.* **2007**, *72*, 6067-6074.

4. Choi, I.; Messinis, A. M.; Hou, X.; Ackermann, L. A Strategy for Site- and Chemoselective C−H Alkenylation through Osmaelectrooxidative Catalysis. *Angew. Chem., Int. Ed.* **2021**, *60*, 27005-27012.

5. Li, X.-R.; Li, W.-D.; Wei, W.-T.; Fan, J.; Liu, Z.-W.; Shi, X.-Y. Sequential Cobalt/Rhodium-Catalyzed Tandem Cyclization of Aromatic Aldehydes with Acrylates for Preparing 3-Substituted Phthalides in Oxygen Atmosphere and Neat Water. *Asian J. Org. Chem.* **2022**, *11*, e202100725.

6. Youn, S. W.; Song, H. S.; Park, J. H. Asymmetric Domino Multicatalysis for the Synthesis of 3-Substituted Phthalides: Cinchonine/NHC Cooperative System. *Org. Lett.* **2014**, *16*, 1028-1031.

7. Dethe, D. H.; Beeralingappa, N. C.; Siddiqui, S. A.; Chavan, P. N. Asymmetric Ru/Cinchonine Dual Catalysis for the One-Pot Synthesis of Optically Active Phthalides from Benzoic Acids and Acrylates. *J. Org. Chem.* **2022**, *87*, 4617-4630.

8. Baruah, S.; Sultana, S.; Bhorali, P.; Saikia, P.; Gogoi, S. Ru(II)-Catalyzed Cascade Decarbonylative Annulation and Dehydrogenative Alkenylation Reactions: Synthesis of Phthalides. *Org. Biomol. Chem.* **2021**, *19*, 2997-3003.

9. Wei, W.; Li, Z.; Li, W.; Li, J.; Shi, X. Green Method for Constructing Phthalides via Oxidative Coupling of Aromatic Acids and Acrylates in Neat Water and Air. *Chin. J. Org. Chem.* **2023**, *43*, 1177-1186.

8. NMR-Spectra

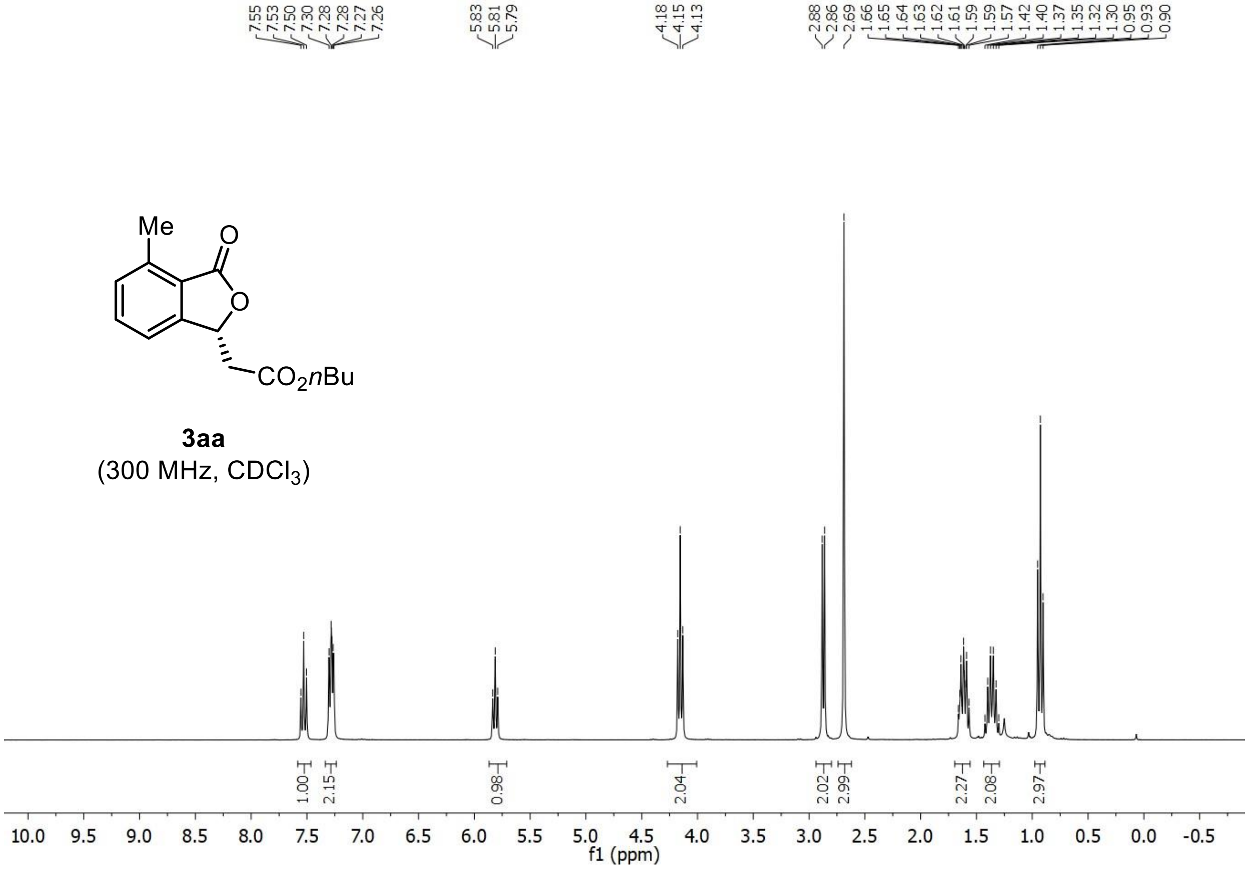

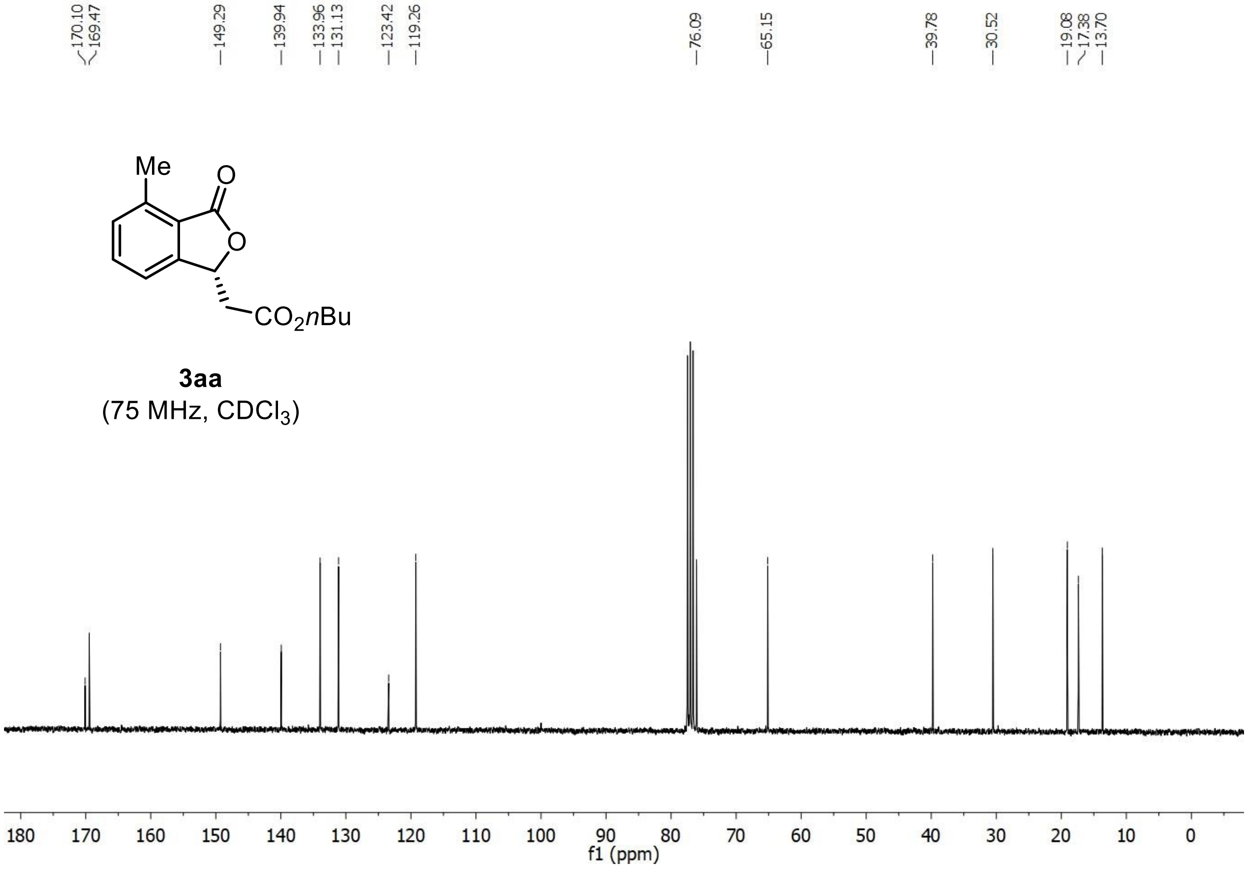

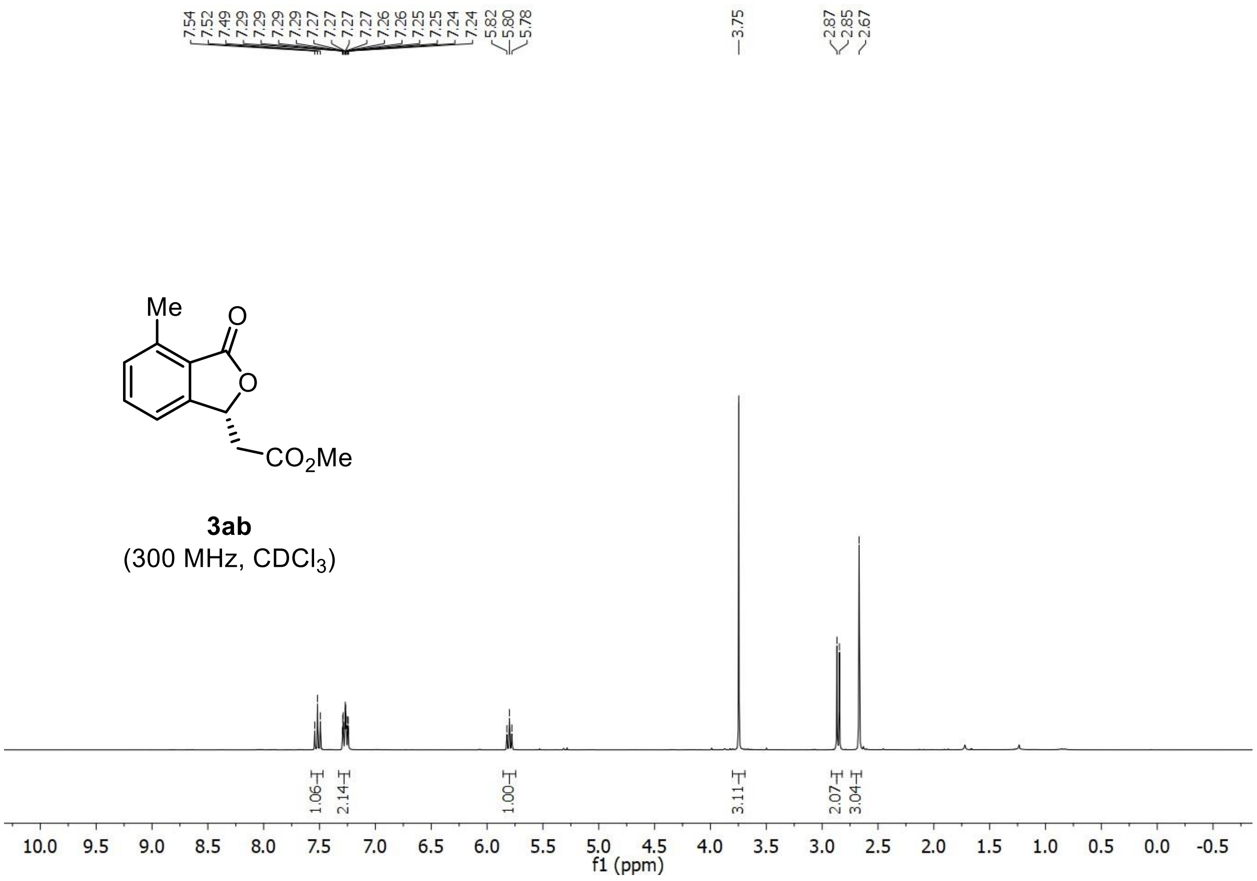

3ab
(300 MHz, $CDCl_3$)

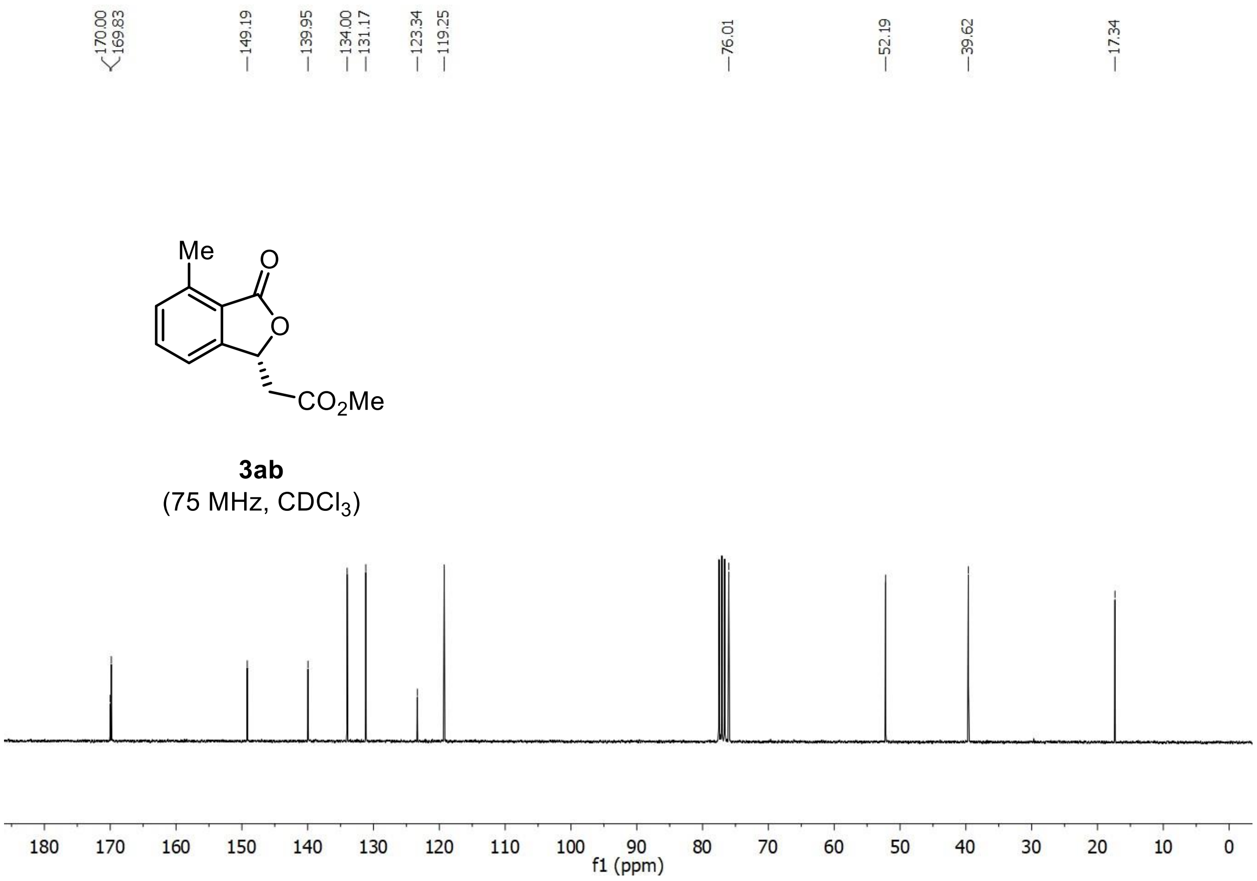

3ab
(75 MHz, $CDCl_3$)

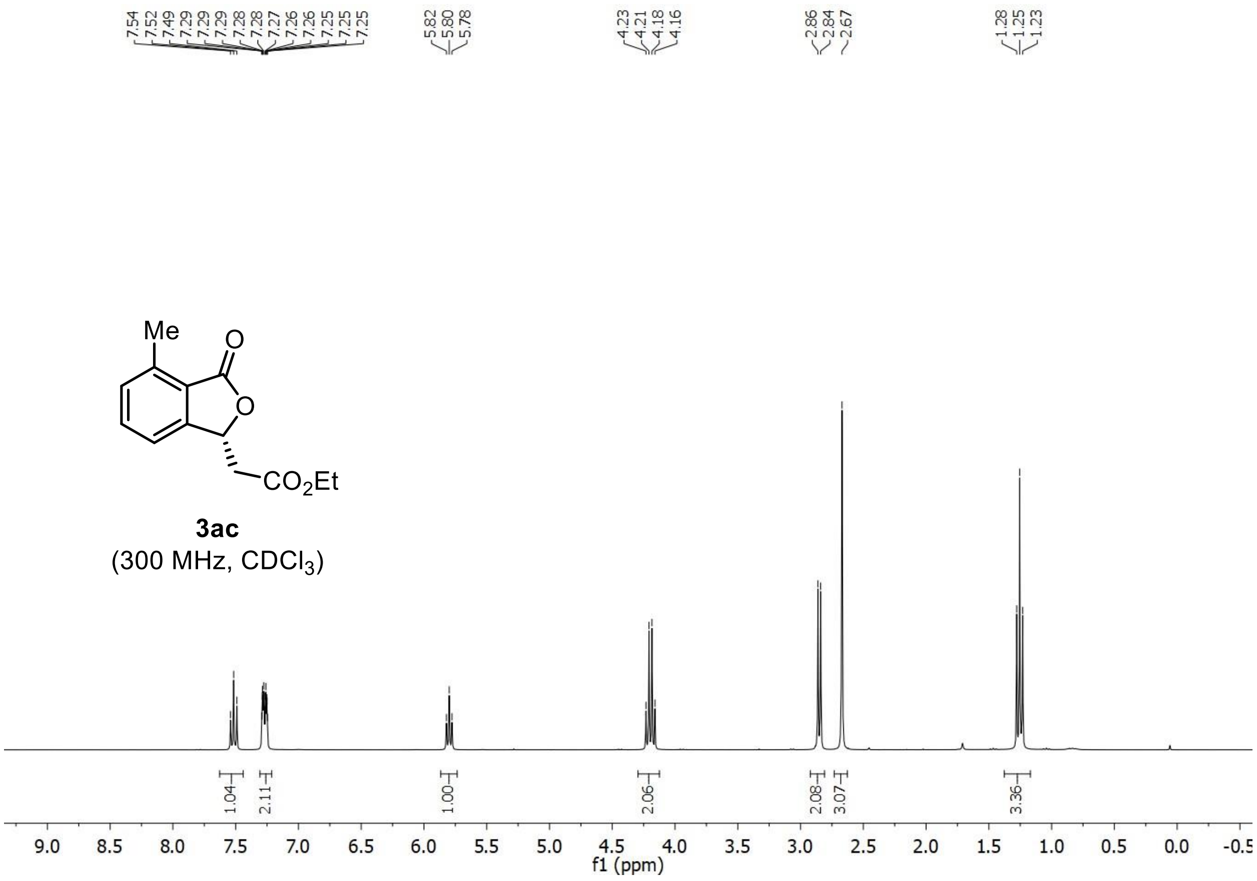
7.54
7.52
7.49
7.29
7.29
7.29
7.28
7.28
7.27
7.26
7.26
7.25
7.25
7.25
5.82
5.80
5.78
4.23
4.21
4.18
4.16
2.86
2.84
2.67
1.28
1.25
1.23
Me
O
O
CO_2Et
3ac
(300 MHz, $CDCl_3$)
1.04
2.11
1.00
2.06
2.08
3.07
3.36
9.0 8.5 8.0 7.5 7.0 6.5 6.0 5.5 5.0 4.5 4.0 3.5 3.0 2.5 2.0 1.5 1.0 0.5 0.0 -0.5
f1 (ppm)

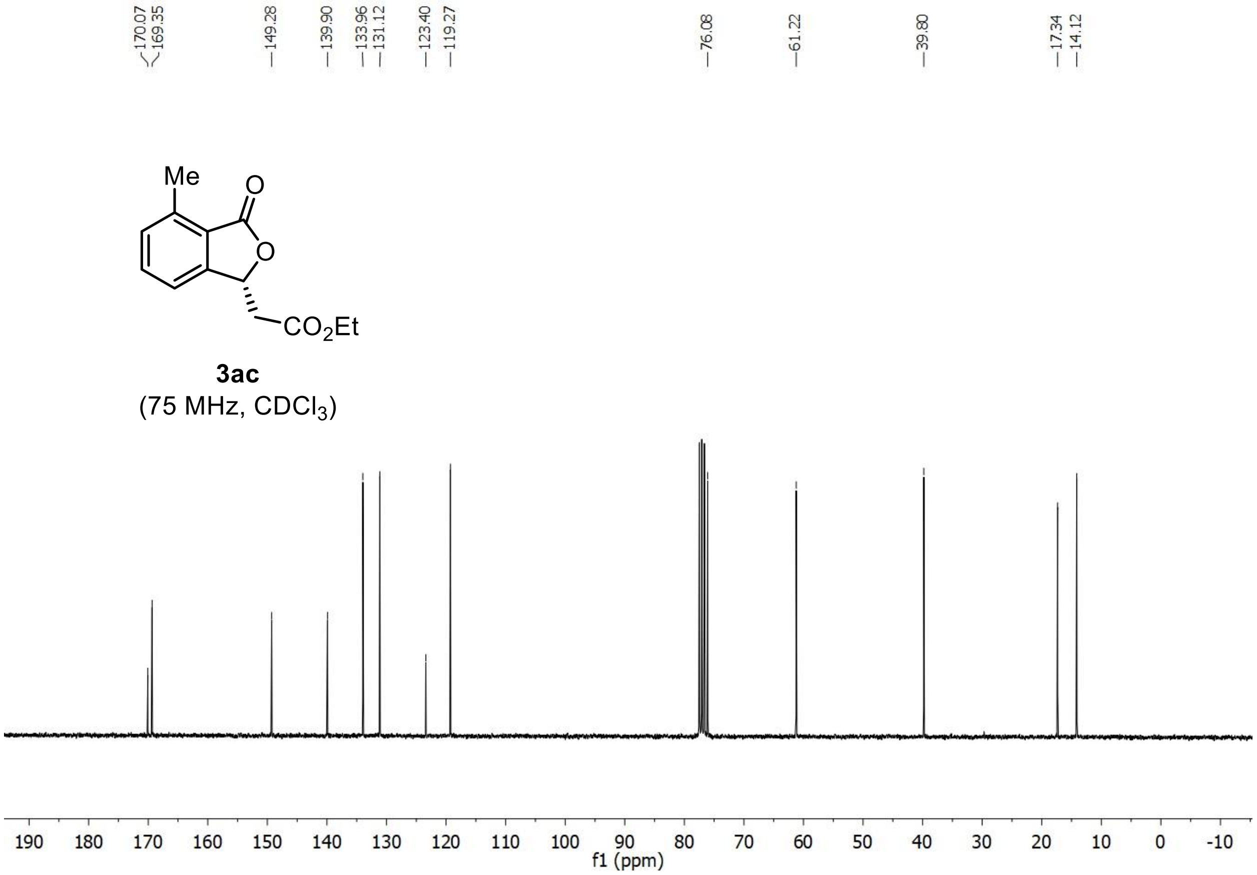
170.07
169.35
149.28
139.90
133.96
131.12
123.40
119.27
76.08
61.22
39.80
17.34
14.12
Me
O
O
CO_2Et
3ac
(75 MHz, $CDCl_3$)
190 180 170 160 150 140 130 120 110 100 90 80 70 60 50 40 30 20 10 0 -10
f1 (ppm)

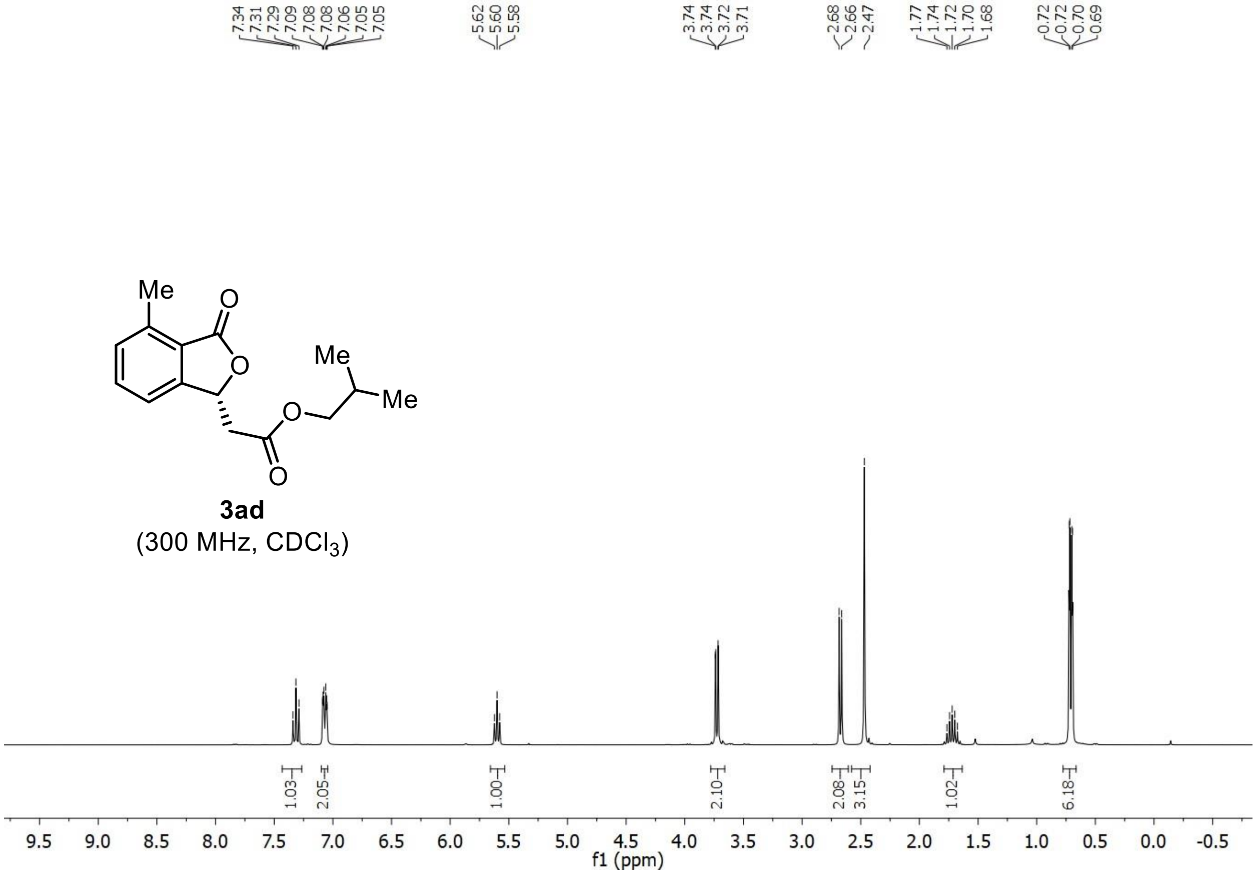
7.34
7.31
7.29
7.09
7.08
7.08
7.06
7.05
7.05
5.62
5.60
5.58
3.74
3.74
3.72
3.71
2.68
2.66
2.47
1.77
1.74
1.72
1.70
1.68
0.72
0.72
0.70
0.69
Me
O
O
Me
O
Me
O
3ad
(300 MHz, CDCl3)
1.03
2.05
1.00
2.10
2.08
3.15
1.02
6.18
f1 (ppm)

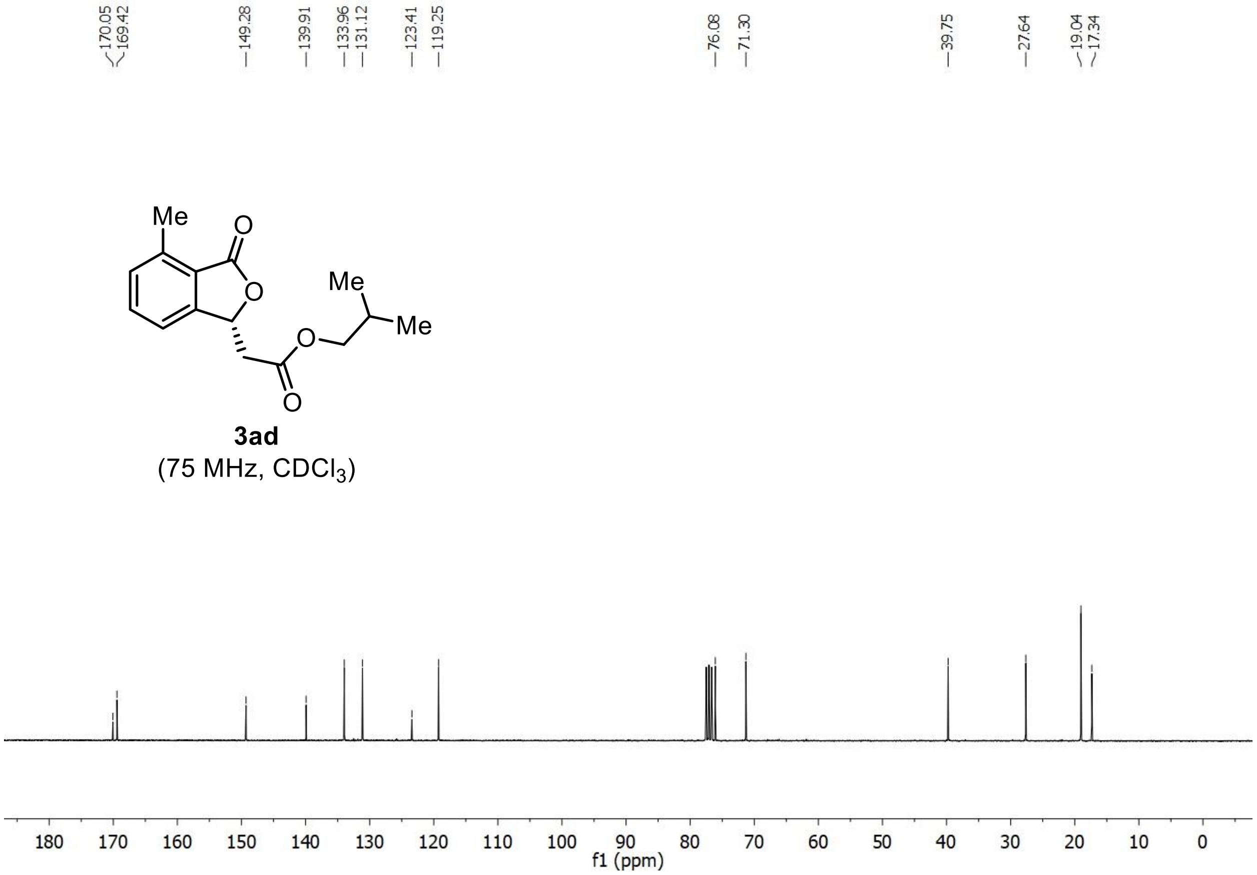
170.05
169.42
149.28
139.91
133.96
131.12
123.41
119.25
76.08
71.30
39.75
27.64
19.04
17.34
Me
O
O
Me
O
Me
O
3ad
(75 MHz, CDCl3)
f1 (ppm)

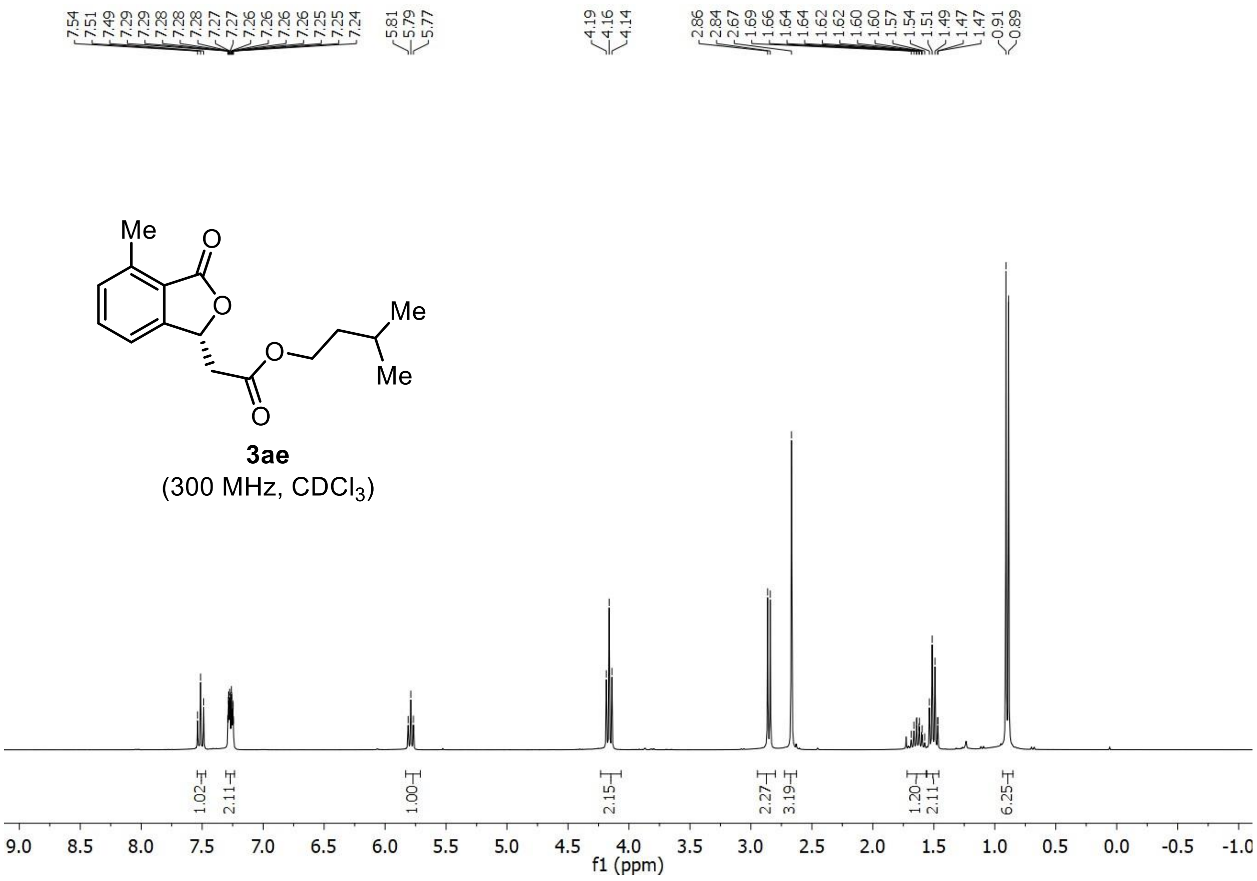
7.54
7.51
7.49
7.29
7.29
7.28
7.28
7.27
7.26
7.26
7.26
7.25
7.25
7.24
5.81
5.79
5.77
4.19
4.16
4.14
2.86
2.84
2.67
1.69
1.66
1.64
1.64
1.62
1.62
1.60
1.60
1.57
1.54
1.51
1.49
1.47
0.91
0.89
Me
O
O
Me
O
Me
O
3ae
(300 MHz, CDCl3)
1.02
2.11
1.00
2.15
2.27
3.19
1.20
2.11
6.25
f1 (ppm)

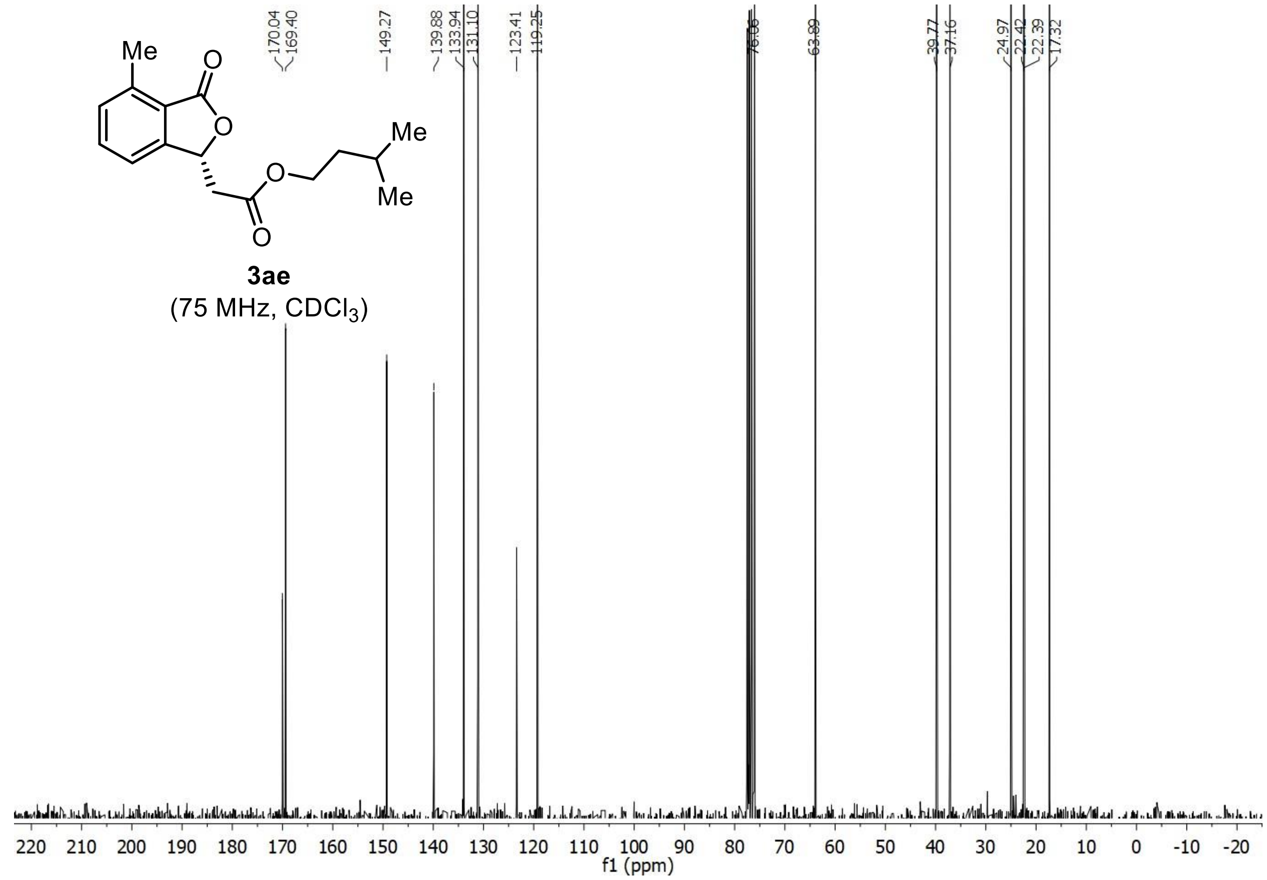
170.04
169.40
149.27
139.88
133.94
131.10
123.41
119.25
63.89
39.77
37.16
24.97
22.42
22.39
17.32
Me
O
O
Me
O
Me
O
3ae
(75 MHz, CDCl3)
f1 (ppm)

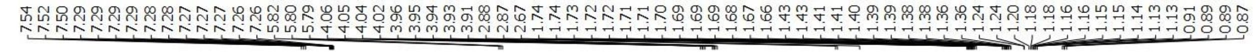

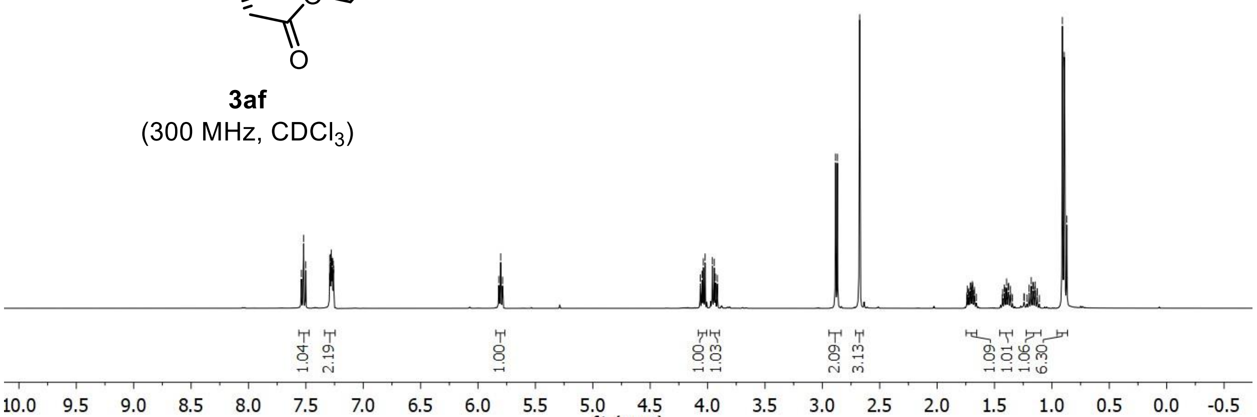

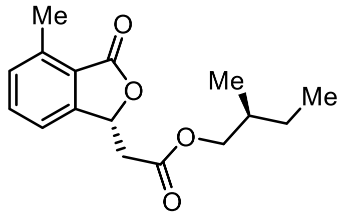

3af
(300 MHz, $CDCl_3$)

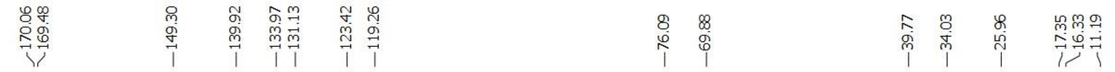

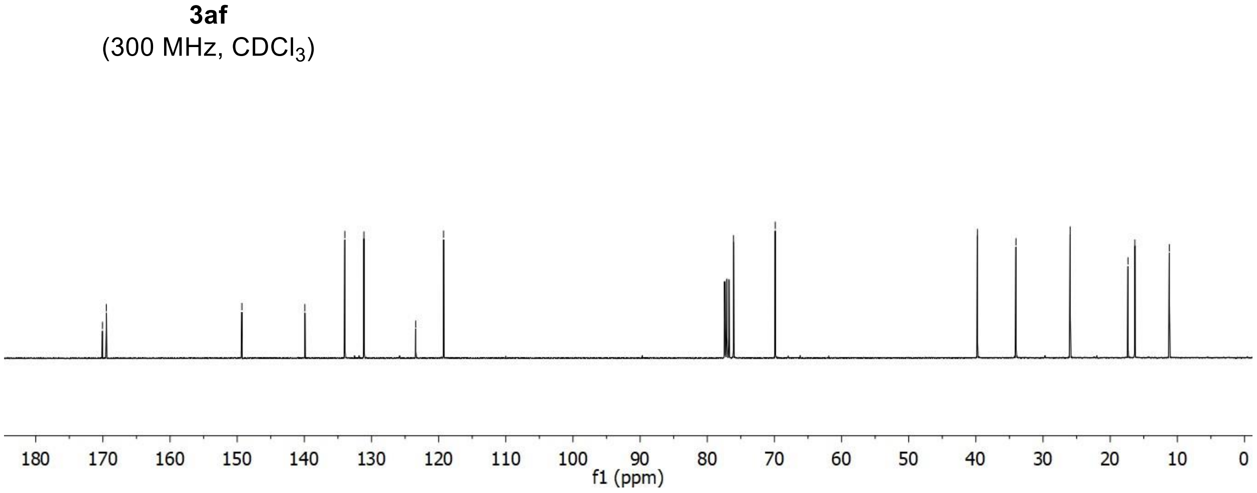

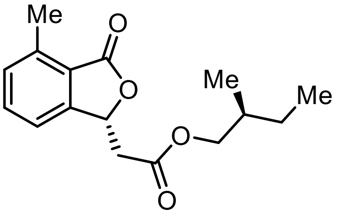

3af
(300 MHz, $CDCl_3$)

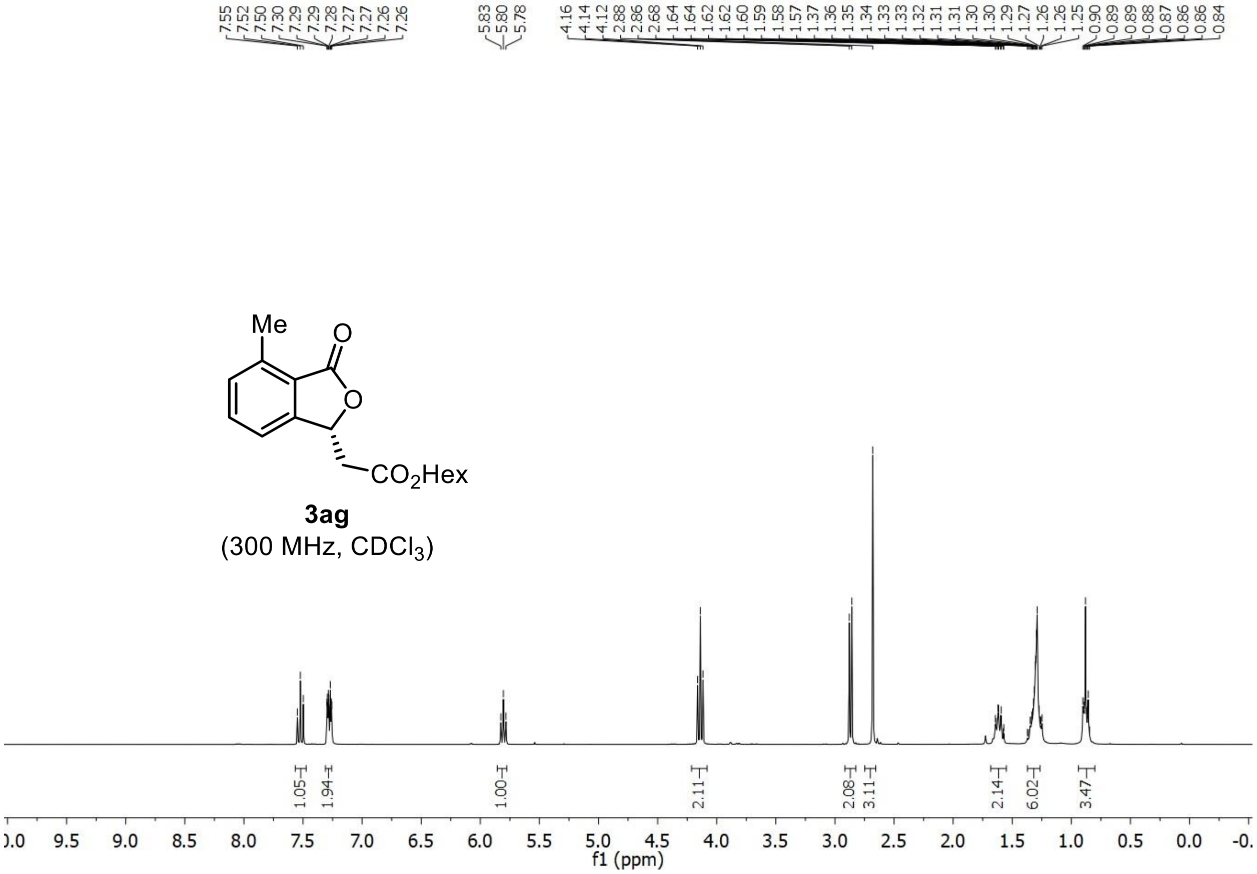
7.55 7.52 7.50 7.30 7.29 7.29 7.28 7.27 7.27 7.26 7.26
5.83 5.80 5.78
4.16 4.14 4.12 2.88 2.86 2.68 1.64 1.64 1.62 1.62 1.60 1.59 1.58 1.57 1.37 1.36 1.35 1.34 1.33 1.33 1.32 1.31 1.31 1.30 1.30 1.29 1.27 1.26 1.25 0.90 0.89 0.89 0.88 0.87 0.86 0.86 0.84
Me
O
O
CO_2Hex
3ag
(300 MHz, $CDCl_3$)
1.05 1.94 1.00 2.11 2.08 3.11 2.14 6.02 3.47
f1 (ppm)

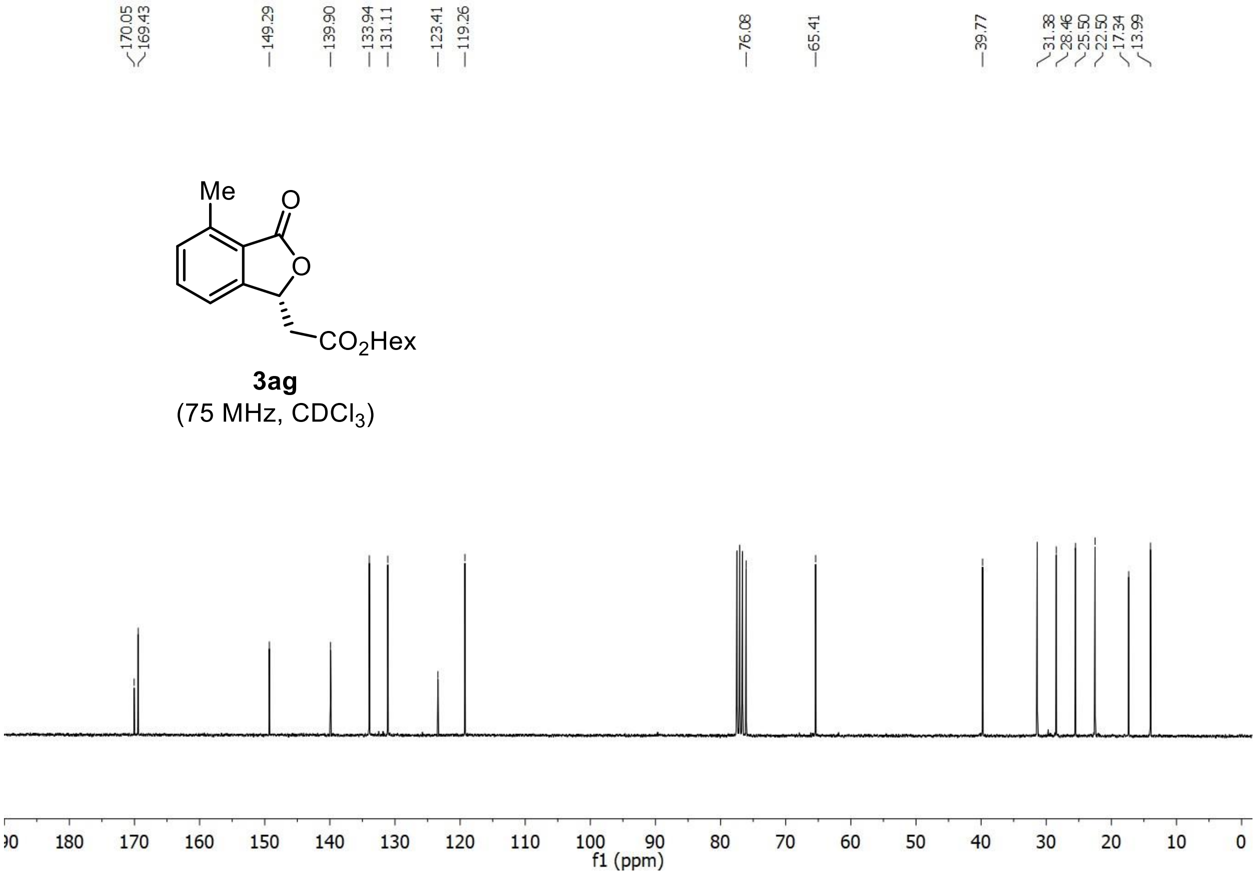
170.05 169.43 149.29 139.90 133.94 131.11 123.41 119.26 76.08 65.41 39.77 31.38 28.46 25.50 22.50 17.34 13.99
Me
O
O
CO_2Hex
3ag
(75 MHz, $CDCl_3$)
f1 (ppm)

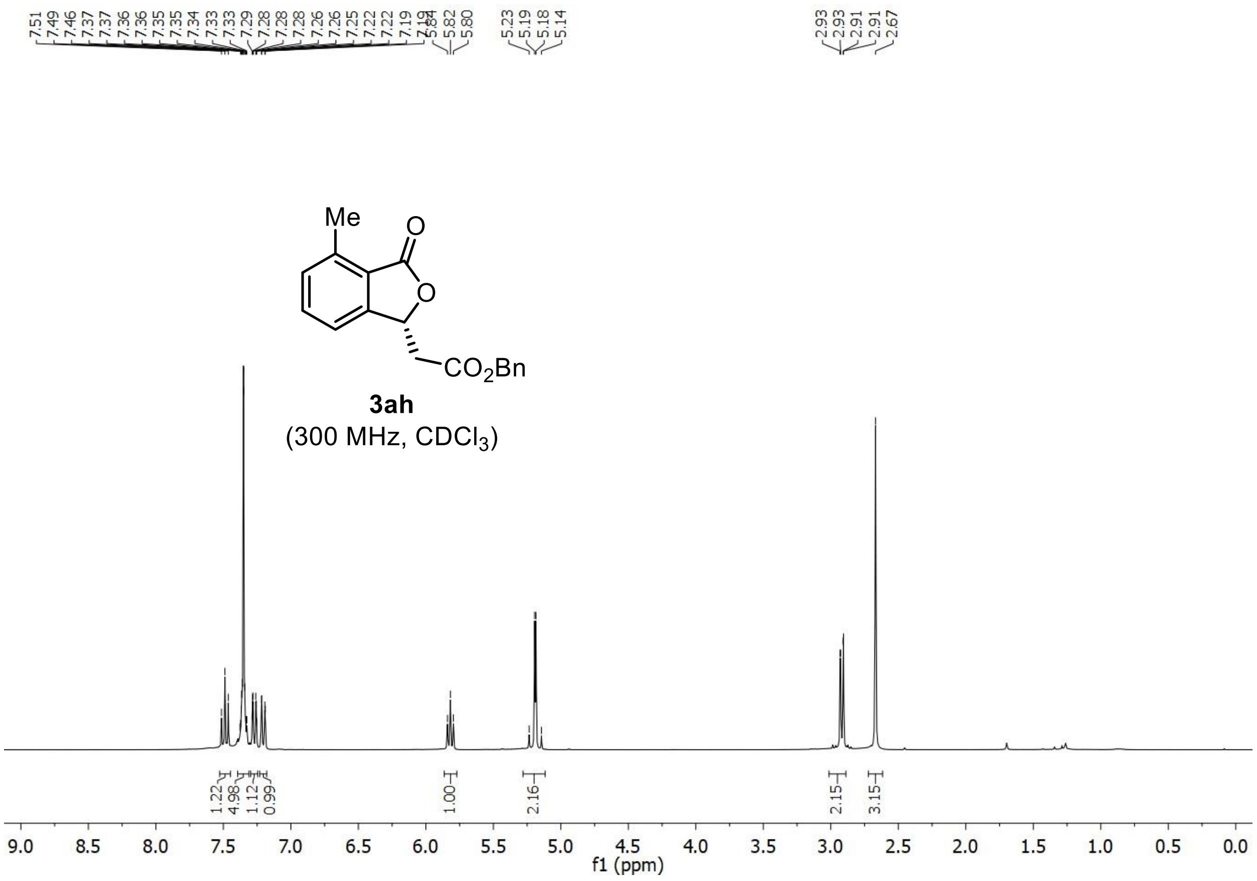
7.51
7.49
7.46
7.37
7.37
7.36
7.36
7.35
7.35
7.34
7.33
7.33
7.29
7.28
7.28
7.28
7.26
7.26
7.25
7.22
7.22
7.19
7.19
5.84
5.82
5.80
5.23
5.19
5.18
5.14
2.93
2.93
2.91
2.91
2.67
Me
O
O
CO2Bn
3ah
(300 MHz, CDCl3)
1.22
4.98
1.12
0.99
1.00
2.16
2.15
3.15
9.0
8.5
8.0
7.5
7.0
6.5
6.0
5.5
5.0
4.5
4.0
3.5
3.0
2.5
2.0
1.5
1.0
0.5
0.0
f1 (ppm)

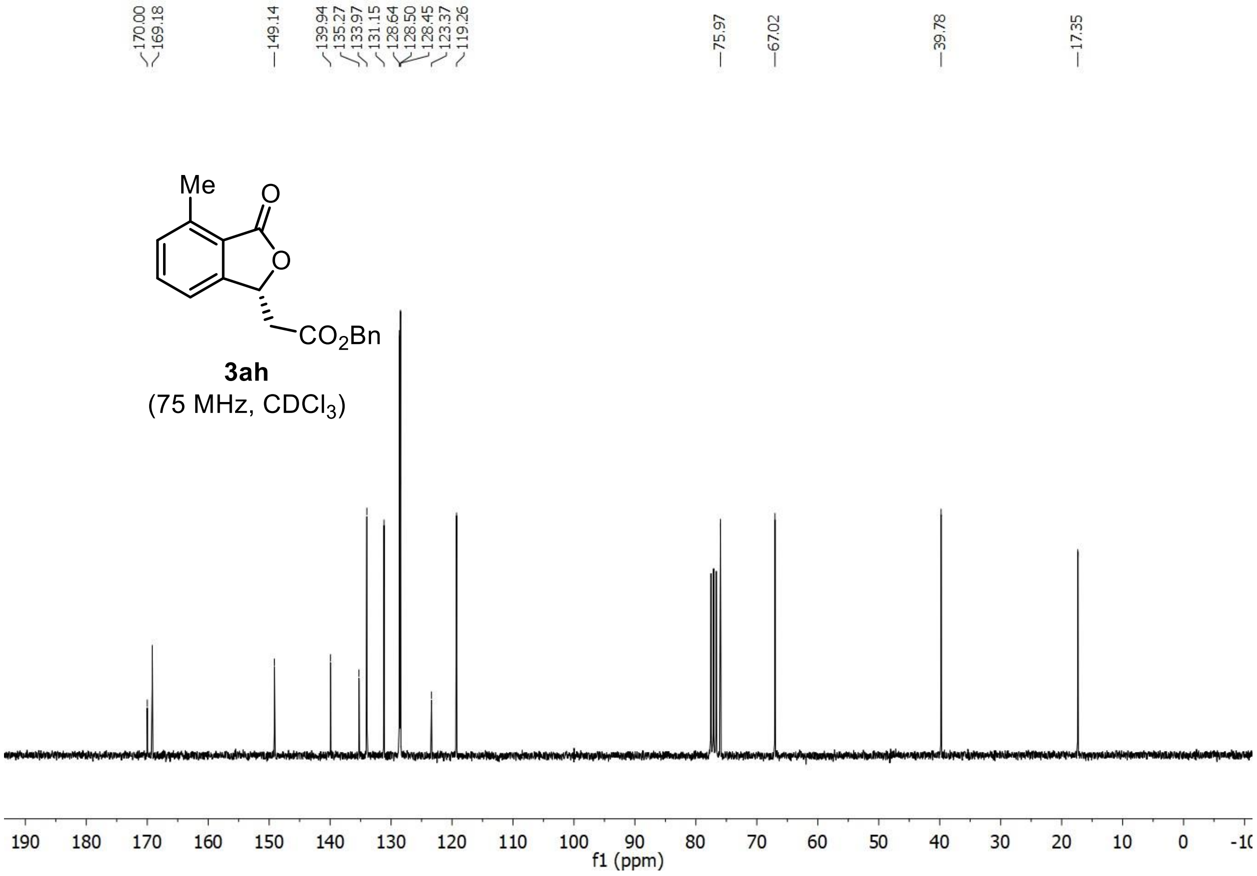
170.00
169.18
149.14
139.94
135.27
133.97
131.15
128.64
128.50
128.45
123.37
119.26
75.97
67.02
39.78
17.35
Me
O
O
CO2Bn
3ah
(75 MHz, CDCl3)
190
180
170
160
150
140
130
120
110
100
90
80
70
60
50
40
30
20
10
0
-10
f1 (ppm)

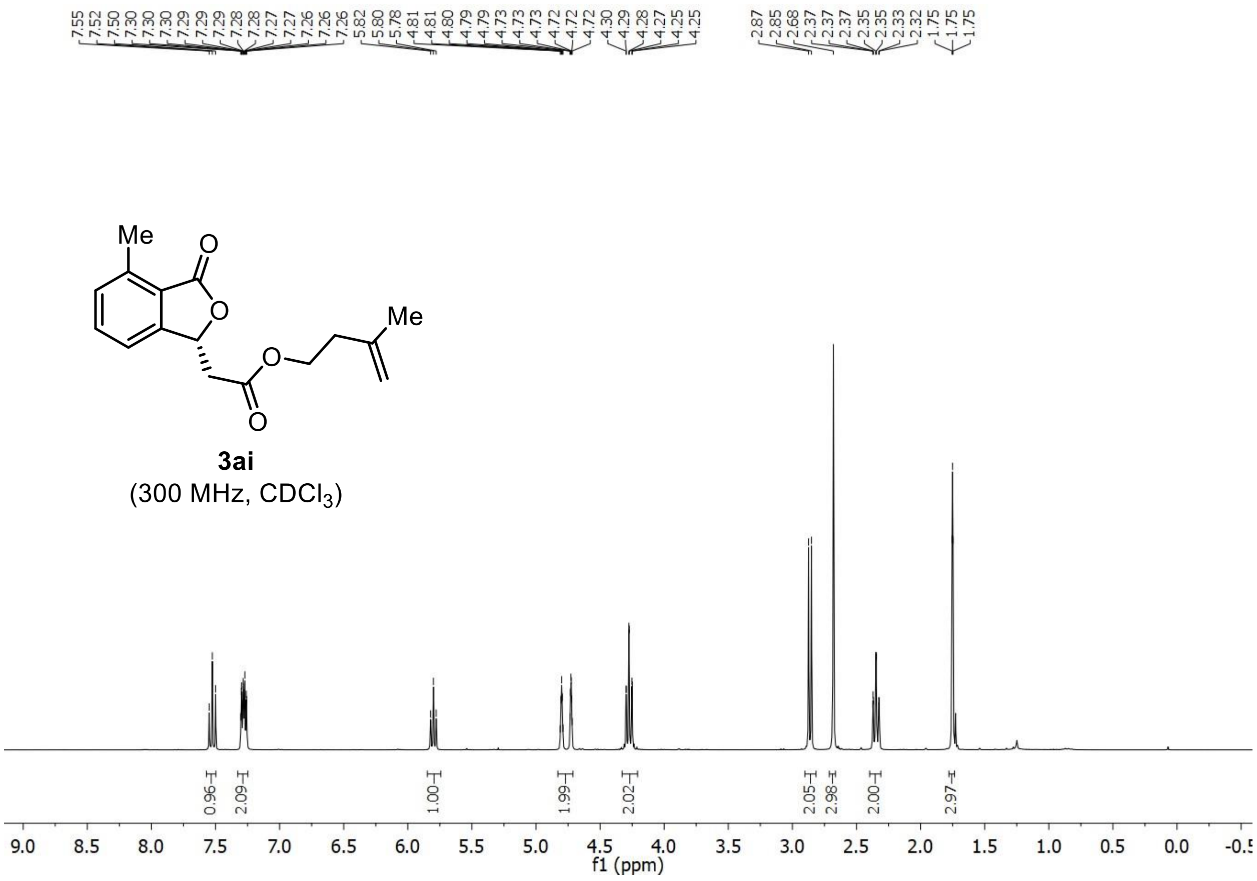
7.55 7.52 7.50 7.30 7.30 7.30 7.29 7.29 7.29 7.28 7.28 7.27 7.27 7.26 7.26 7.26 5.82 5.80 5.78 4.81 4.81 4.80 4.79 4.79 4.73 4.73 4.73 4.72 4.72 4.72 4.30 4.29 4.28 4.27 4.25 4.25 2.87 2.85 2.68 2.37 2.37 2.37 2.35 2.35 2.33 2.32 1.75 1.75 1.75
Me
O
O
O
Me
O
3ai
(300 MHz, CDCl3)
0.96 2.09 1.00 1.99 2.02 2.05 2.98 2.00 2.97
9.0 8.5 8.0 7.5 7.0 6.5 6.0 5.5 5.0 4.5 4.0 3.5 3.0 2.5 2.0 1.5 1.0 0.5 0.0 -0.5
f1 (ppm)

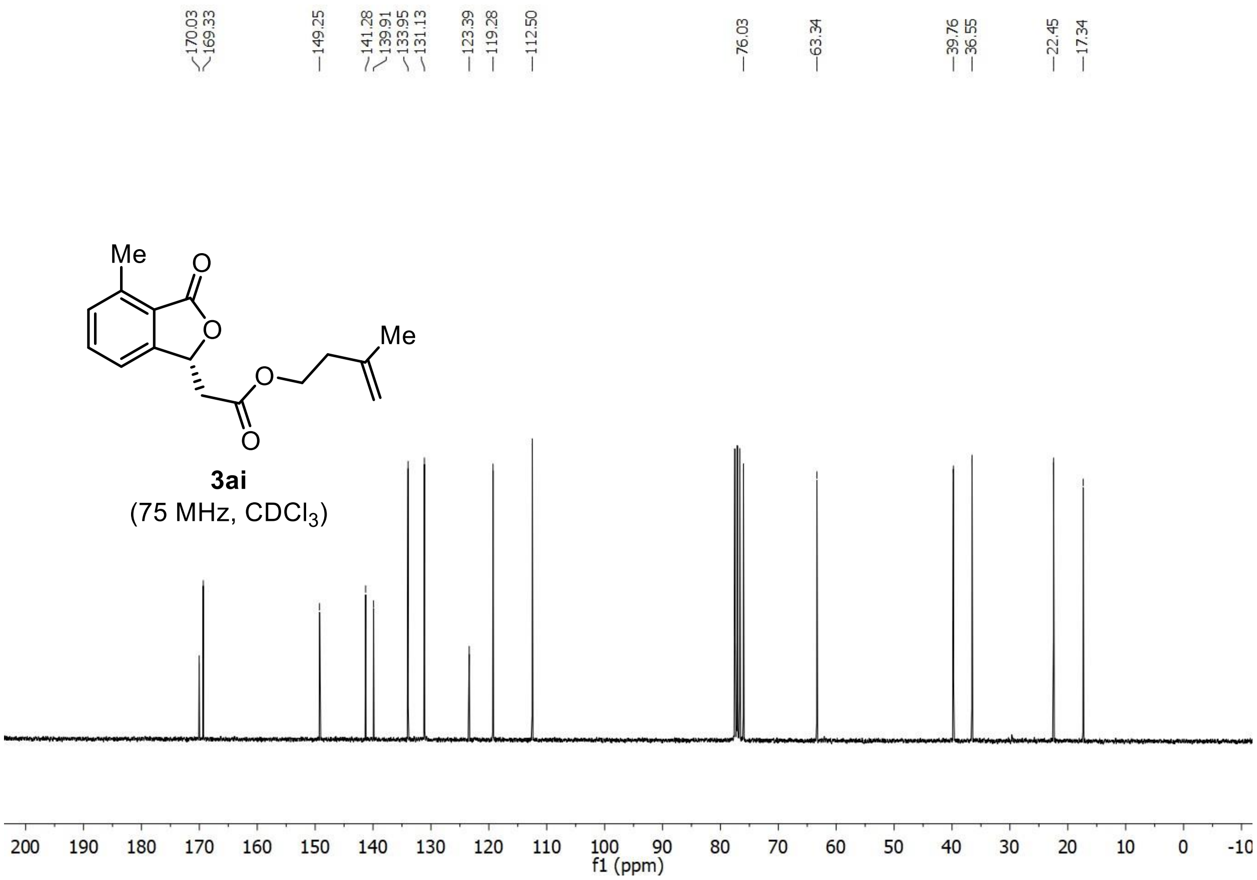
170.03 169.33 149.25 141.28 139.91 133.95 131.13 123.39 119.28 112.50 76.03 63.34 39.76 36.55 22.45 17.34
Me
O
O
O
Me
O
3ai
(75 MHz, CDCl3)
200 190 180 170 160 150 140 130 120 110 100 90 80 70 60 50 40 30 20 10 0 -10
f1 (ppm)

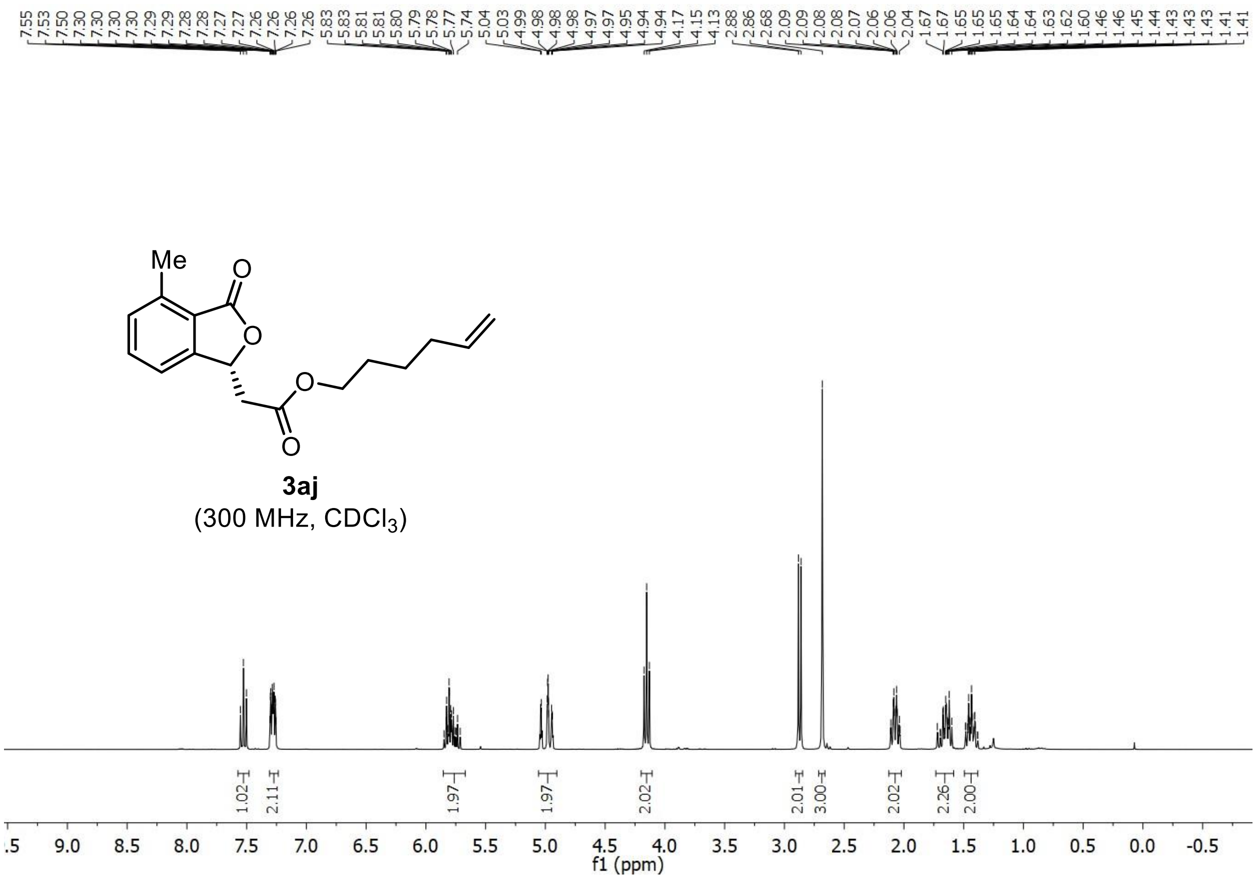
7.55 7.53 7.50 7.30 7.30 7.30 7.29 7.29 7.28 7.28 7.27 7.27 7.26 7.26 7.26 7.26 5.83 5.83 5.81 5.81 5.80 5.79 5.78 5.77 5.74 5.04 5.03 4.99 4.98 4.98 4.98 4.97 4.97 4.95 4.94 4.94 4.17 4.15 4.13 2.88 2.86 2.68 2.09 2.09 2.08 2.08 2.07 2.06 2.06 2.04 1.67 1.67 1.65 1.65 1.64 1.64 1.63 1.62 1.60 1.46 1.46 1.45 1.44 1.43 1.43 1.43 1.41 1.41
Me
O
O
O
O
3aj
(300 MHz, $CDCl_3$)
1.02
2.11
1.97
1.97
2.02
2.01
3.00
2.02
2.26
2.00
.5 9.0 8.5 8.0 7.5 7.0 6.5 6.0 5.5 5.0 4.5 4.0 3.5 3.0 2.5 2.0 1.5 1.0 0.5 0.0 -0.5
f1 (ppm)

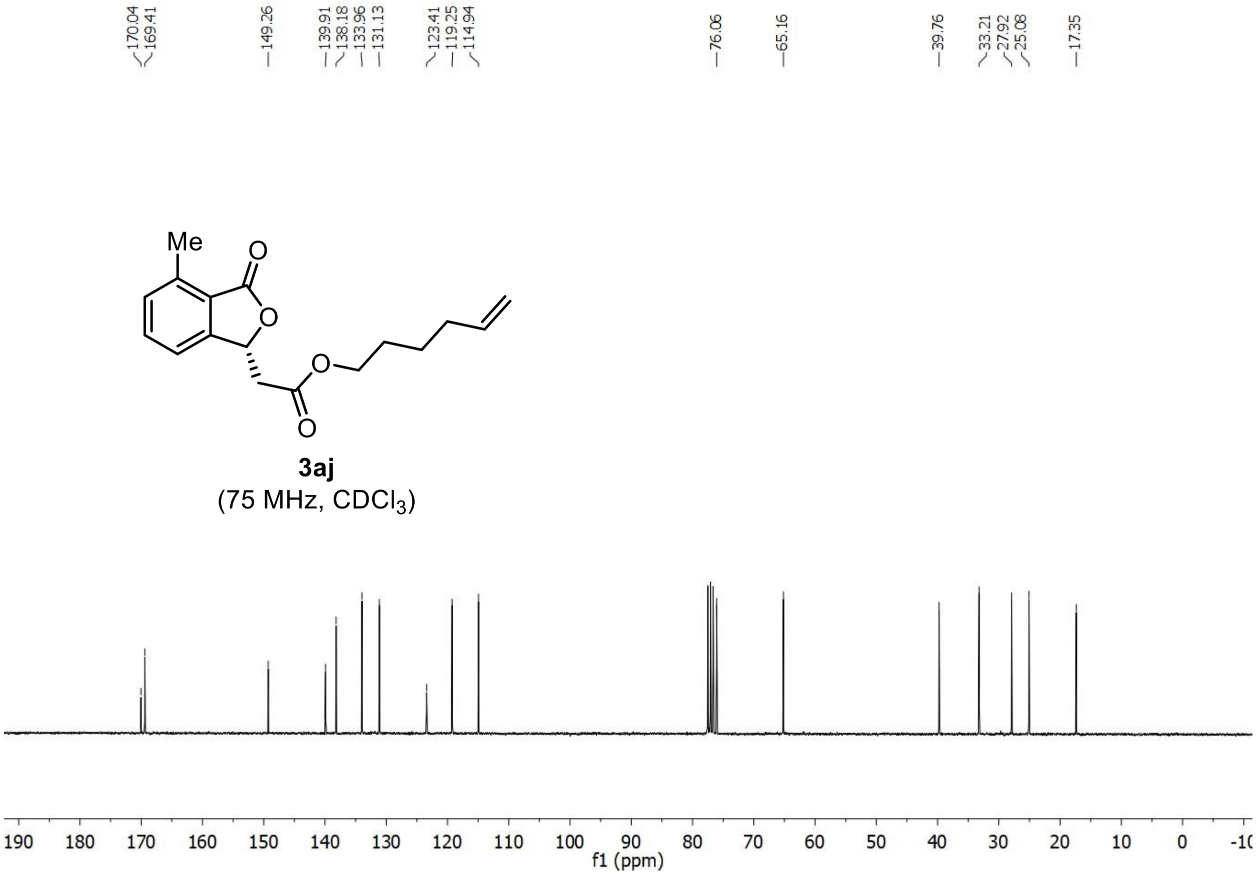
170.04 169.41 149.26 139.91 138.18 133.96 131.13 123.41 119.25 114.94 76.06 65.16 39.76 33.21 27.92 25.08 17.35
Me
O
O
O
O
3aj
(75 MHz, $CDCl_3$)
190 180 170 160 150 140 130 120 110 100 90 80 70 60 50 40 30 20 10 0 -1(
f1 (ppm)

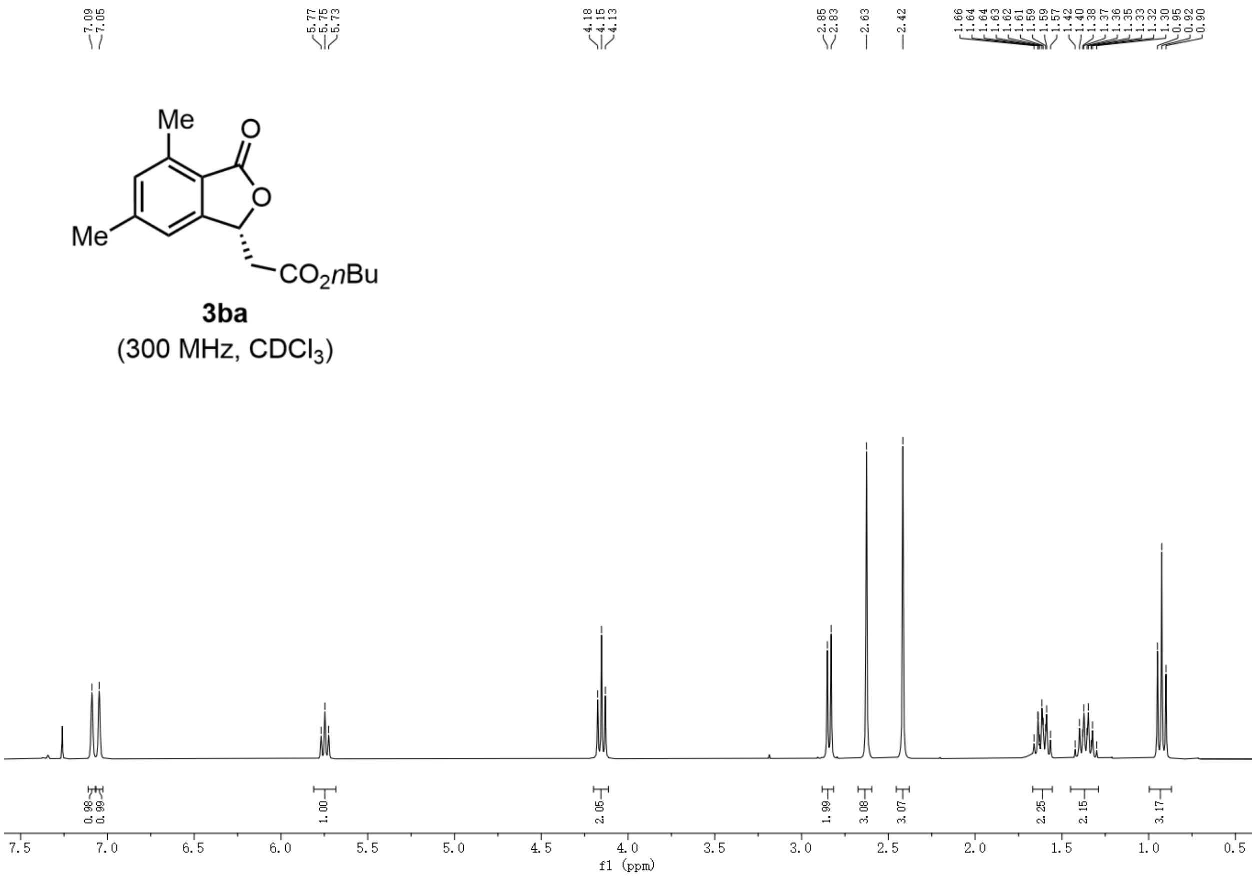
7.09
7.05
5.77
5.75
5.73
4.18
4.15
4.13
2.85
2.83
2.63
2.42
1.66
1.64
1.63
1.62
1.61
1.59
1.59
1.57
1.42
1.40
1.38
1.37
1.36
1.35
1.33
1.32
1.30
0.95
0.92
0.90
Me
O
O
Me
CO2nBu
3ba
(300 MHz, CDCl3)
0.98
0.99
1.00
2.05
1.99
3.08
3.07
2.25
2.15
3.17
7.5
7.0
6.5
6.0
5.5
5.0
4.5
4.0
3.5
3.0
2.5
2.0
1.5
1.0
0.5
f1 (ppm)

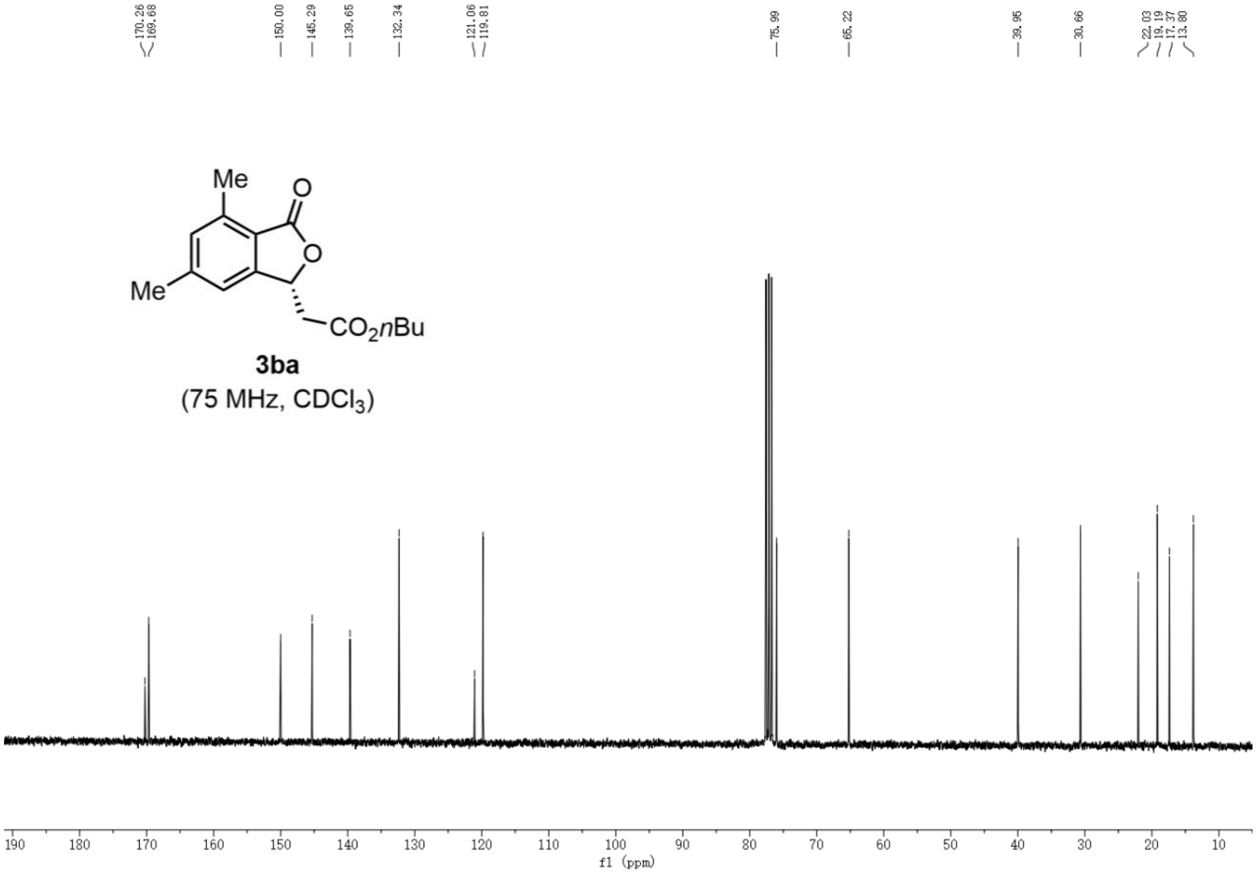
170.26
169.68
150.00
145.29
139.65
132.34
121.06
119.81
75.99
65.22
39.95
30.66
22.03
19.19
17.37
13.80
Me
O
O
Me
CO2nBu
3ba
(75 MHz, CDCl3)
190
180
170
160
150
140
130
120
110
100
90
80
70
60
50
40
30
20
10
f1 (ppm)

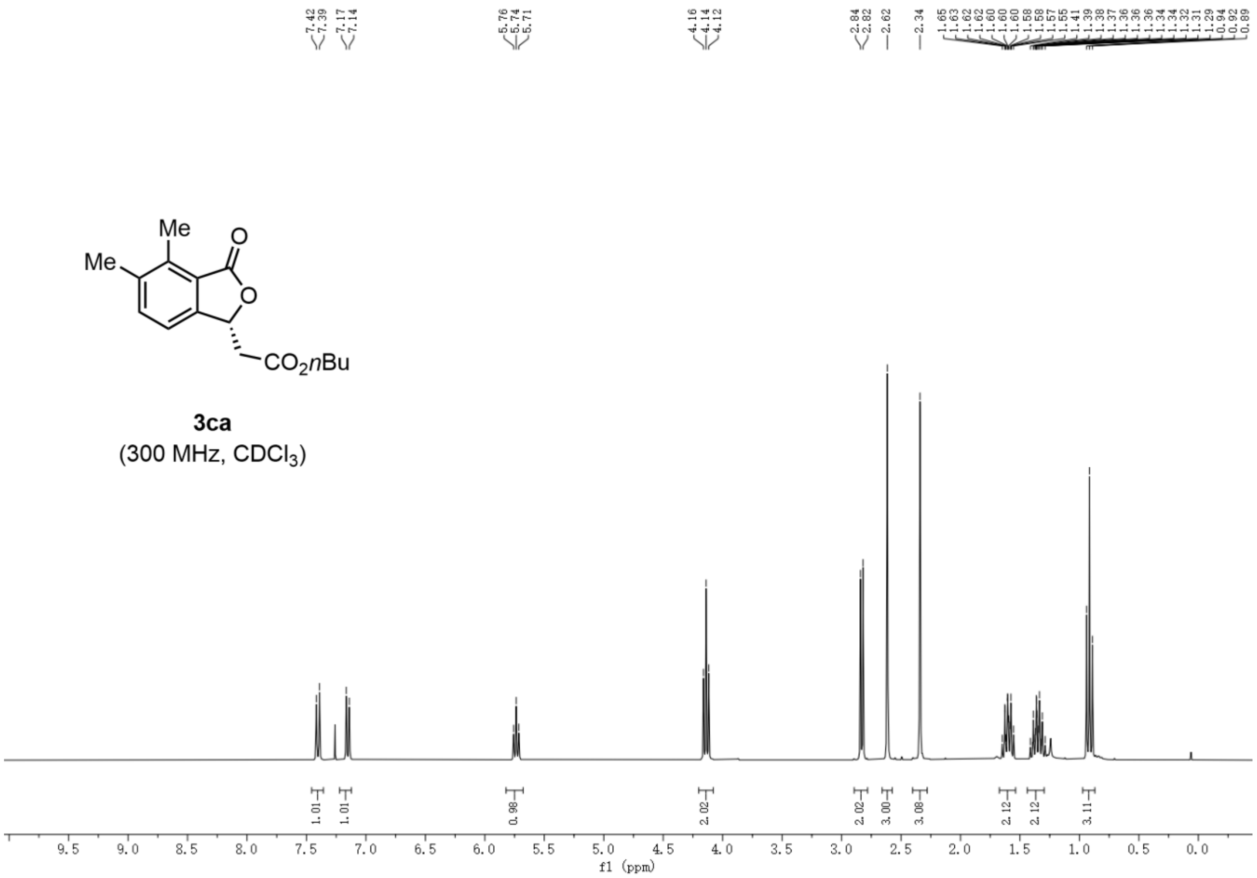
3ca
(300 MHz, CDCl3)
f1 (ppm)

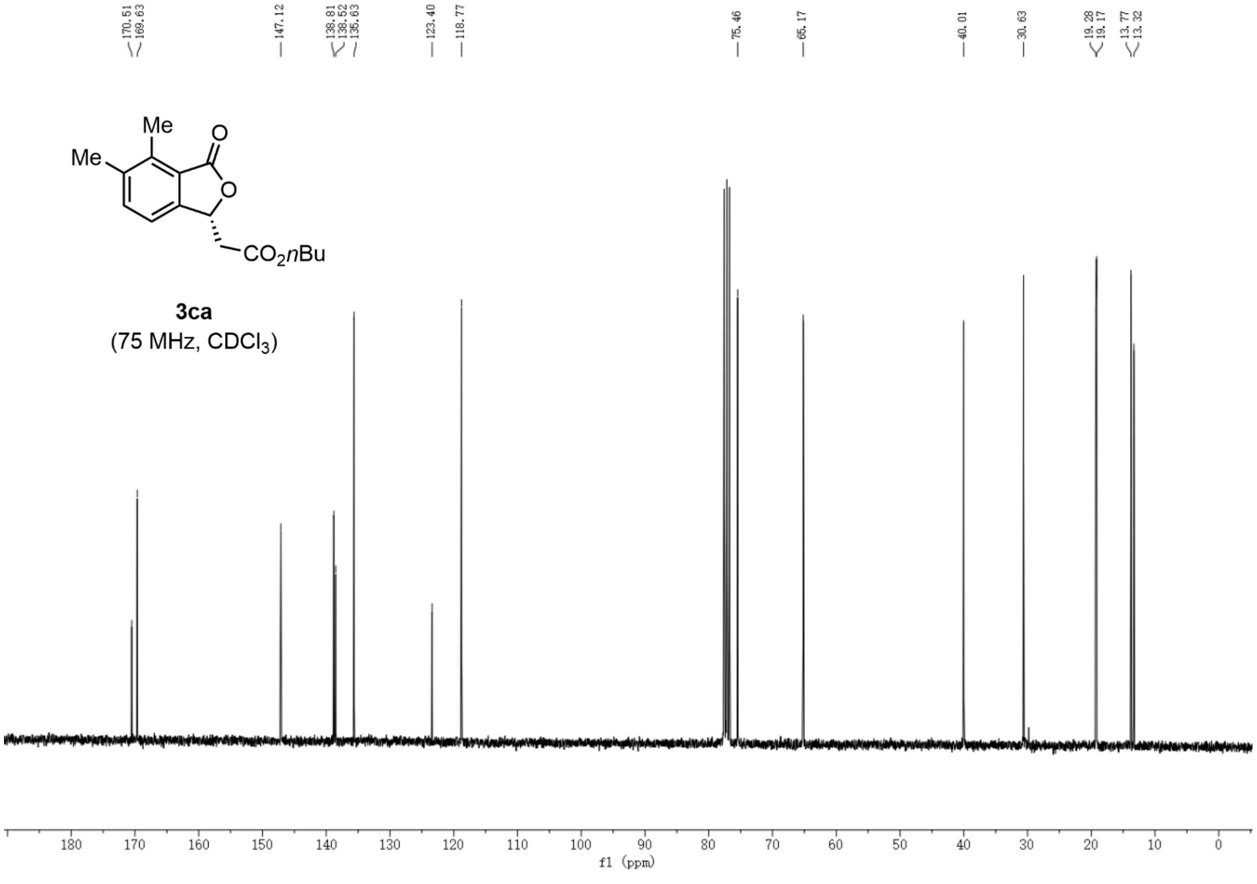
3ca
(75 MHz, CDCl3)
f1 (ppm)

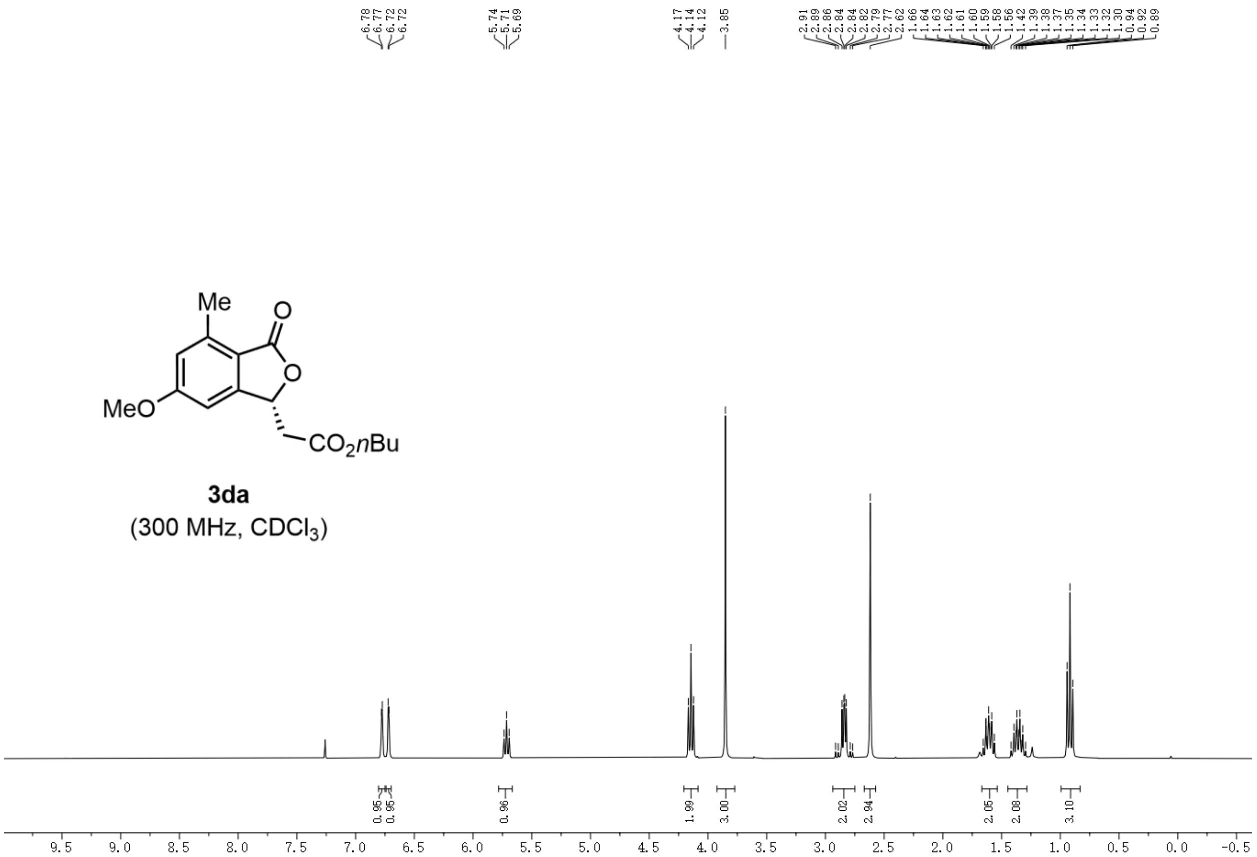
6.78
6.77
6.72
6.72
5.74
5.71
5.69
4.17
4.14
4.12
3.85
2.91
2.89
2.86
2.84
2.84
2.82
2.79
2.77
2.62
1.66
1.64
1.63
1.62
1.61
1.60
1.59
1.58
1.56
1.42
1.39
1.38
1.37
1.35
1.34
1.33
1.32
1.30
0.94
0.92
0.89
Me
O
O
MeO
CO2nBu
3da
(300 MHz, CDCl3)
0.95
0.95
0.96
1.99
3.00
2.02
2.94
2.05
2.08
3.10
9.5 9.0 8.5 8.0 7.5 7.0 6.5 6.0 5.5 5.0 4.5 4.0 3.5 3.0 2.5 2.0 1.5 1.0 0.5 0.0 -0.5
f1 (ppm)

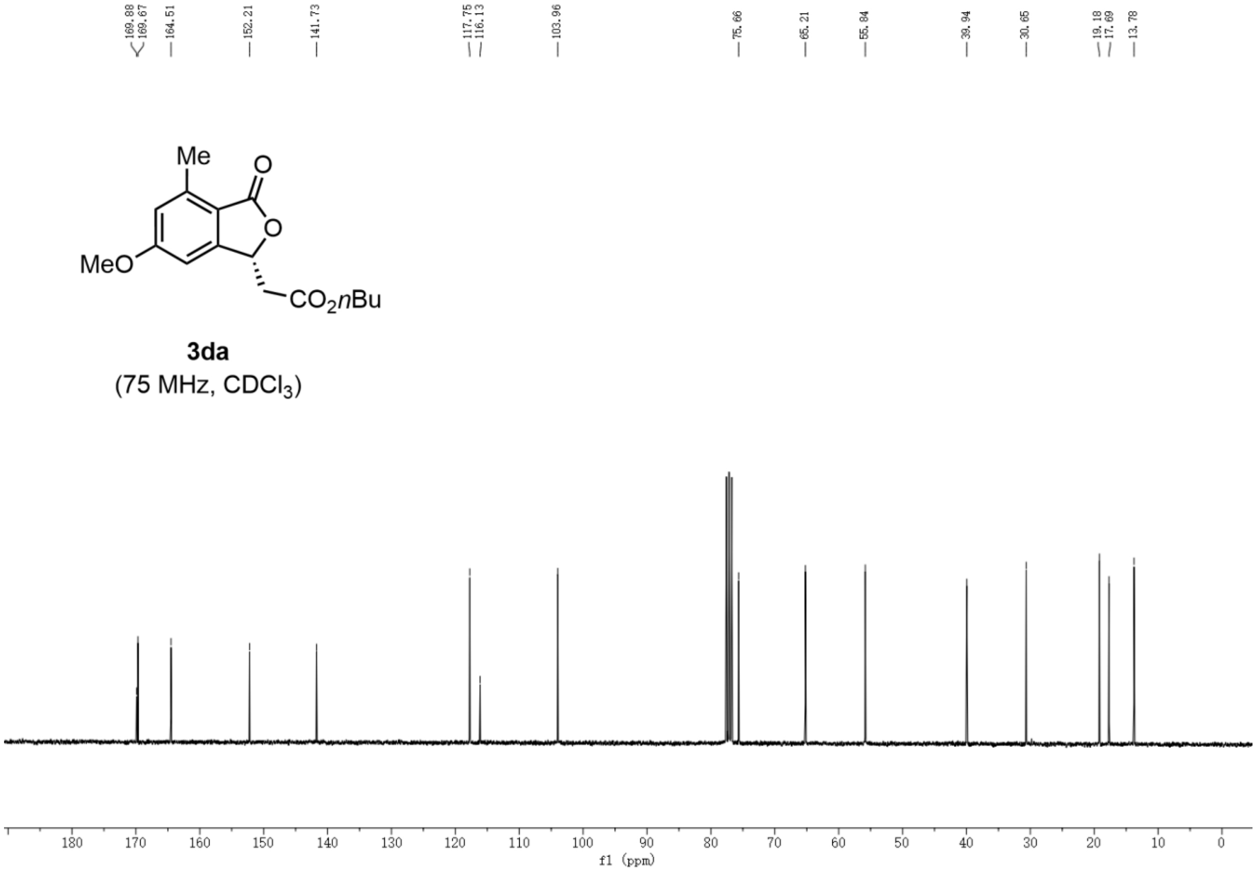
169.88
169.67
164.51
152.21
141.73
117.75
116.13
103.96
75.66
65.21
55.84
39.94
30.65
19.18
17.69
13.78
Me
O
O
MeO
CO2nBu
3da
(75 MHz, CDCl3)
180 170 160 150 140 130 120 110 100 90 80 70 60 50 40 30 20 10 0
f1 (ppm)

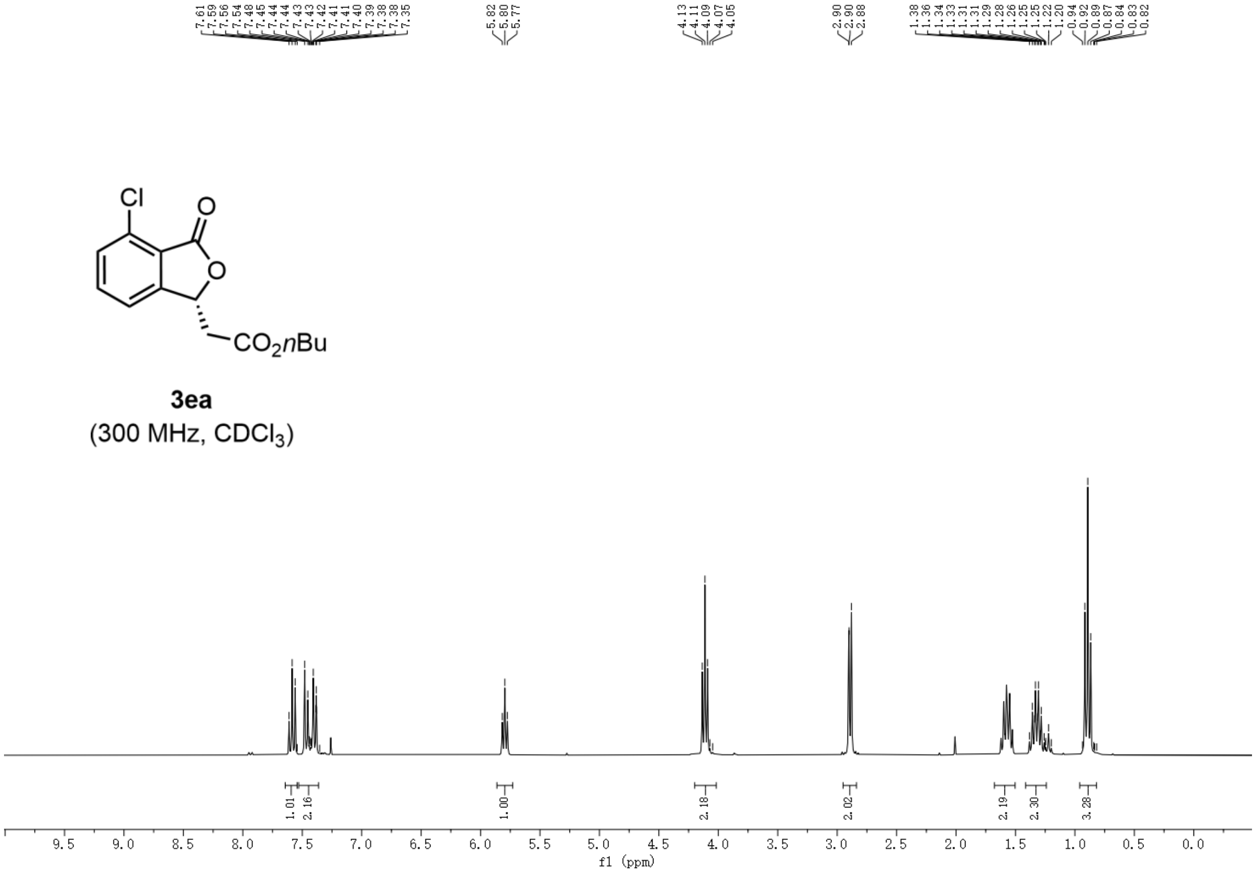

3ea
(300 MHz, $CDCl_3$)

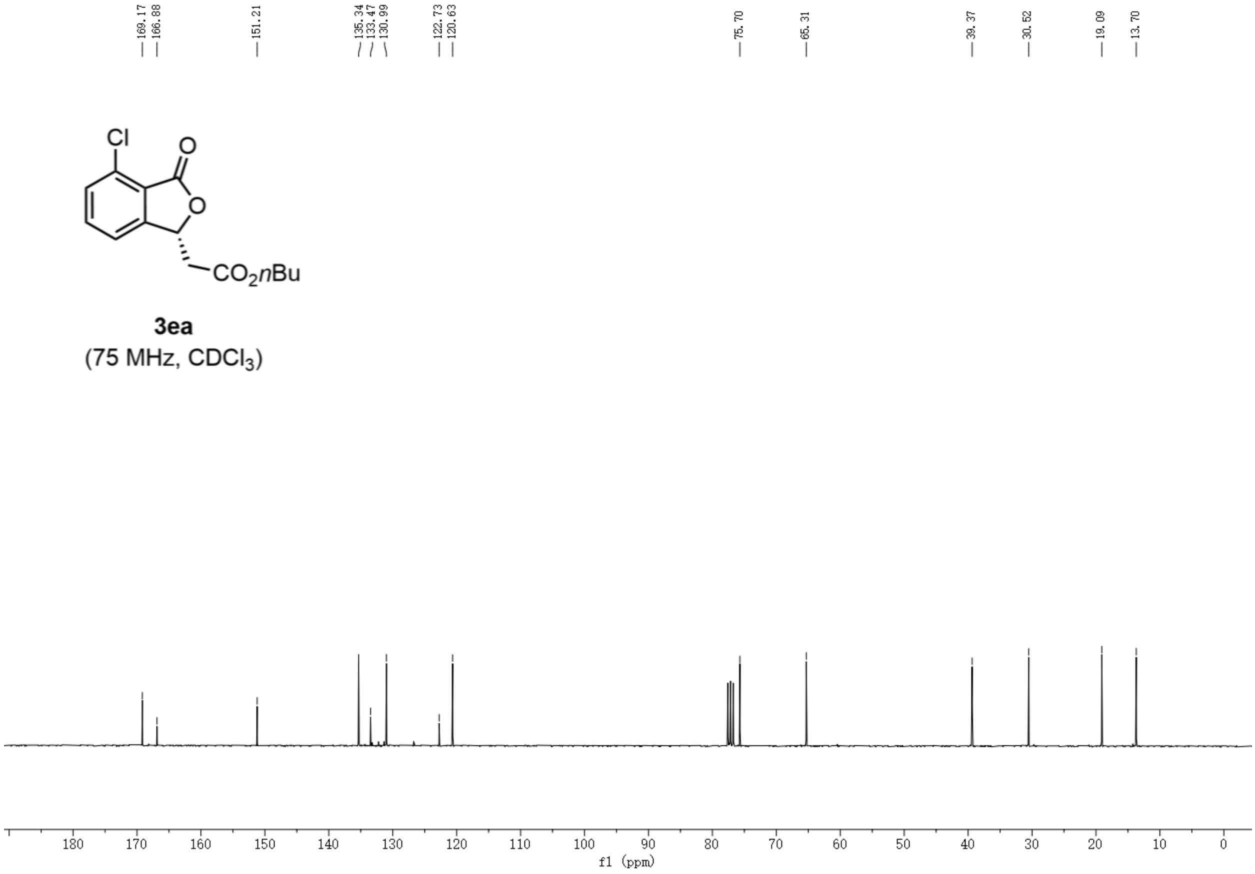

3ea
(75 MHz, $CDCl_3$)

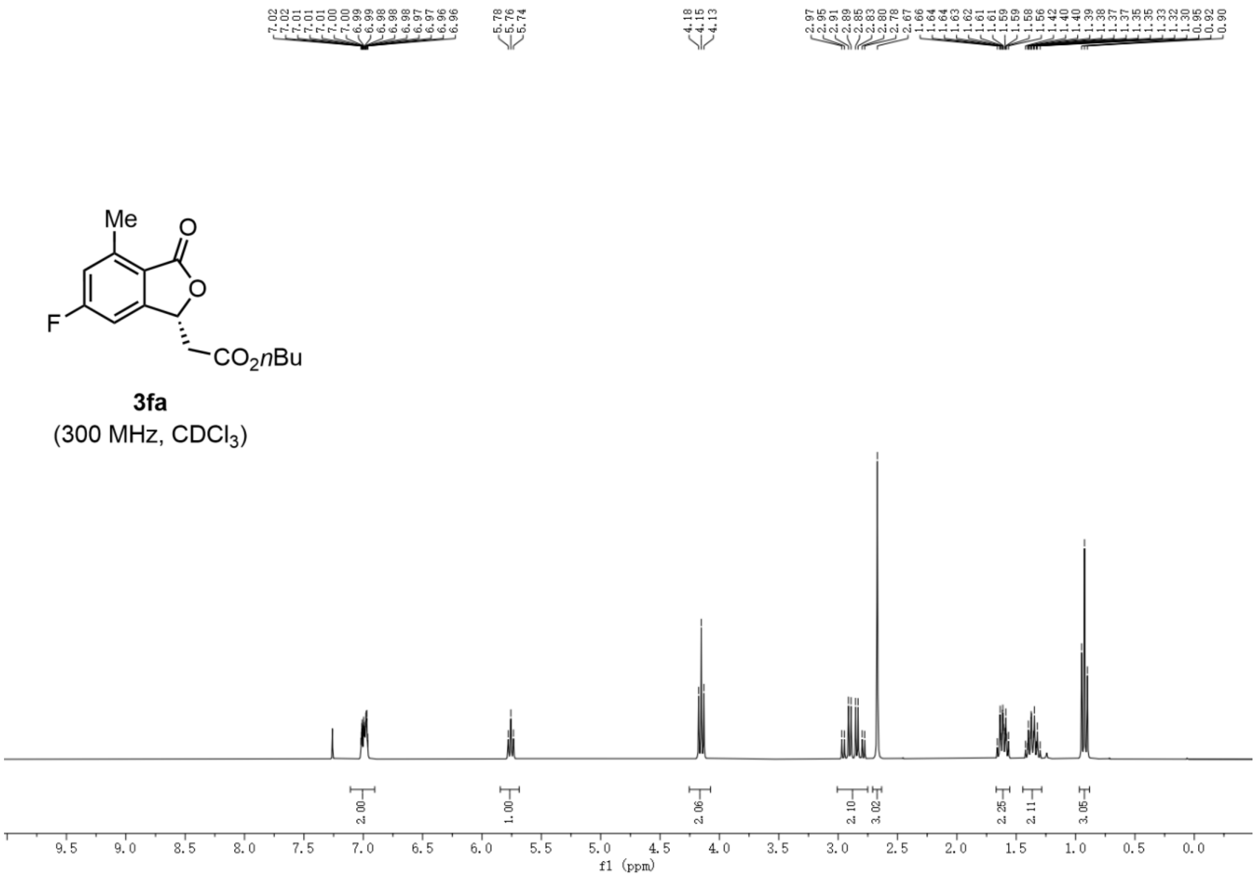
7.02
7.02
7.01
7.01
7.00
7.00
6.99
6.99
6.98
6.98
6.98
6.97
6.96
6.96
5.78
5.76
5.74
4.18
4.15
4.13
2.97
2.95
2.91
2.89
2.85
2.83
2.80
2.78
2.67
1.66
1.64
1.64
1.63
1.62
1.61
1.61
1.59
1.59
1.58
1.56
1.42
1.40
1.39
1.38
1.37
1.37
1.35
1.35
1.33
1.32
1.30
0.95
0.92
0.90
Me
O
O
F
CO2nBu
3fa
(300 MHz, CDCl3)
2.00
1.00
2.06
2.10
3.02
2.25
2.11
3.06
f1 (ppm)

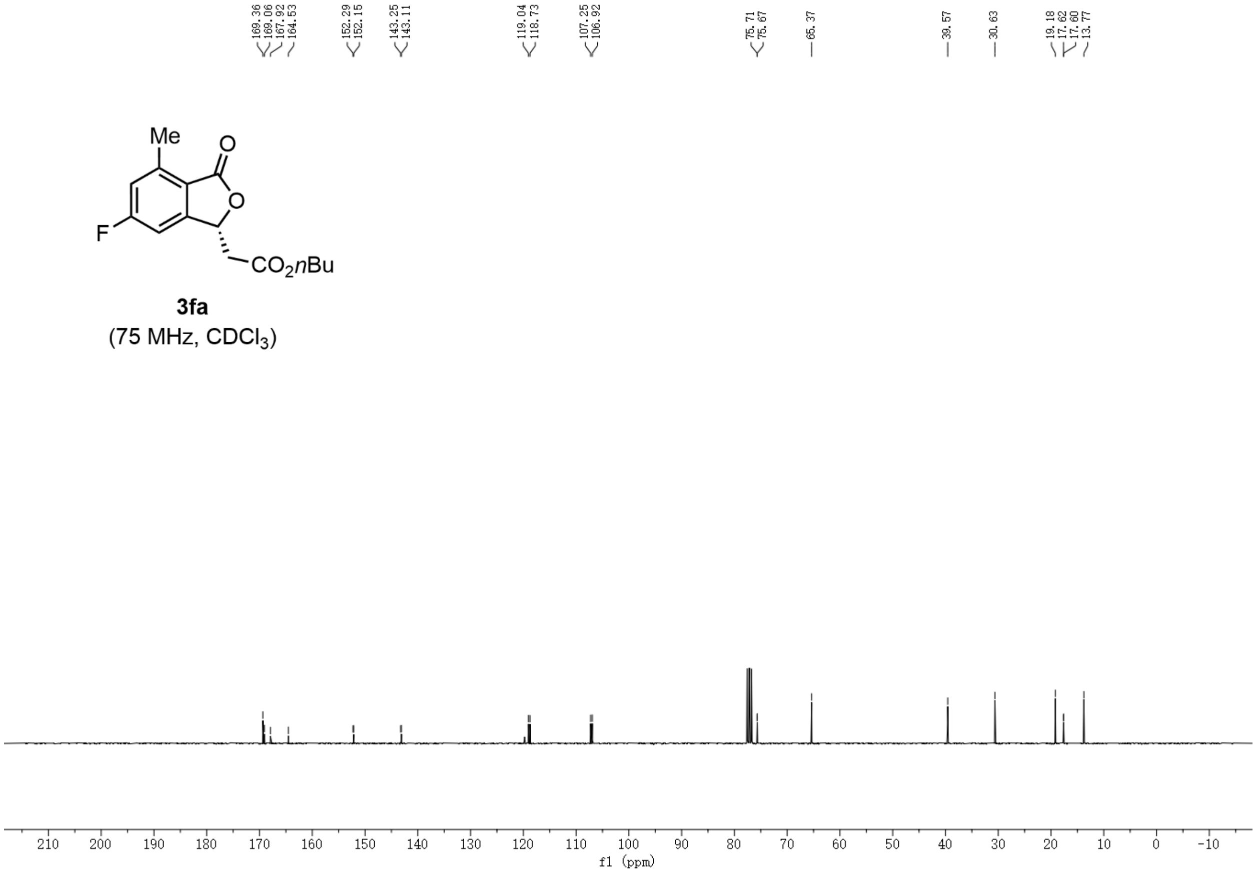
169.36
169.06
167.92
164.53
152.29
152.15
143.25
143.11
119.04
118.73
107.25
106.92
75.71
75.67
65.37
39.57
30.63
19.18
17.62
17.60
13.77
Me
O
O
F
CO2nBu
3fa
(75 MHz, CDCl3)
f1 (ppm)

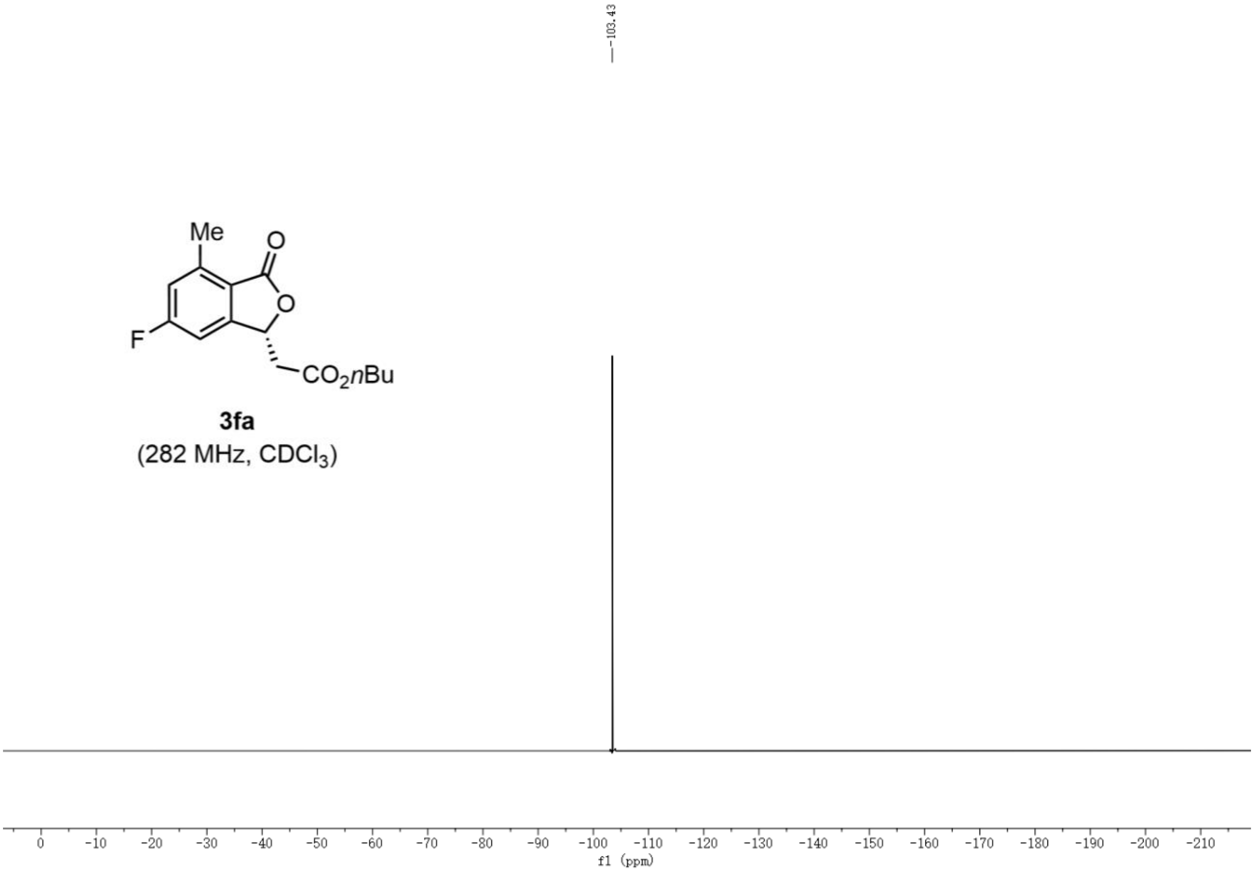

3fa
(282 MHz, $CDCl_3$)

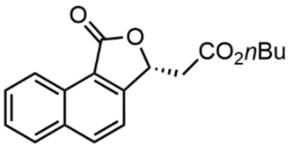

3ga
(400 MHz, $CDCl_3$)

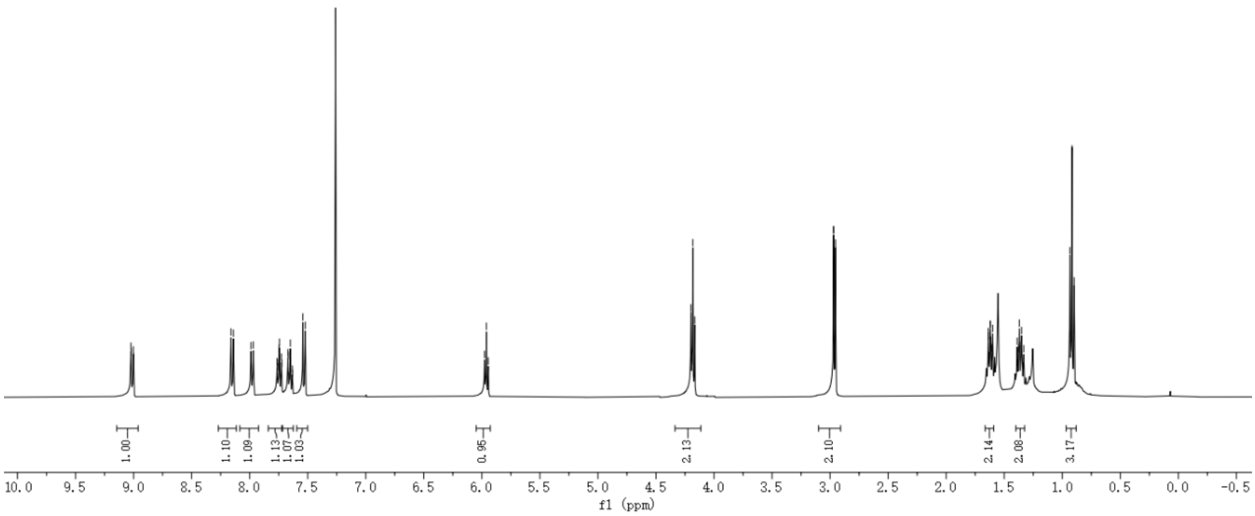

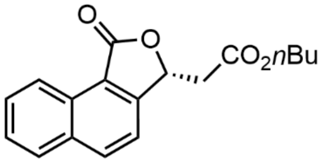

3ga
(101 MHz, $CDCl_3$)

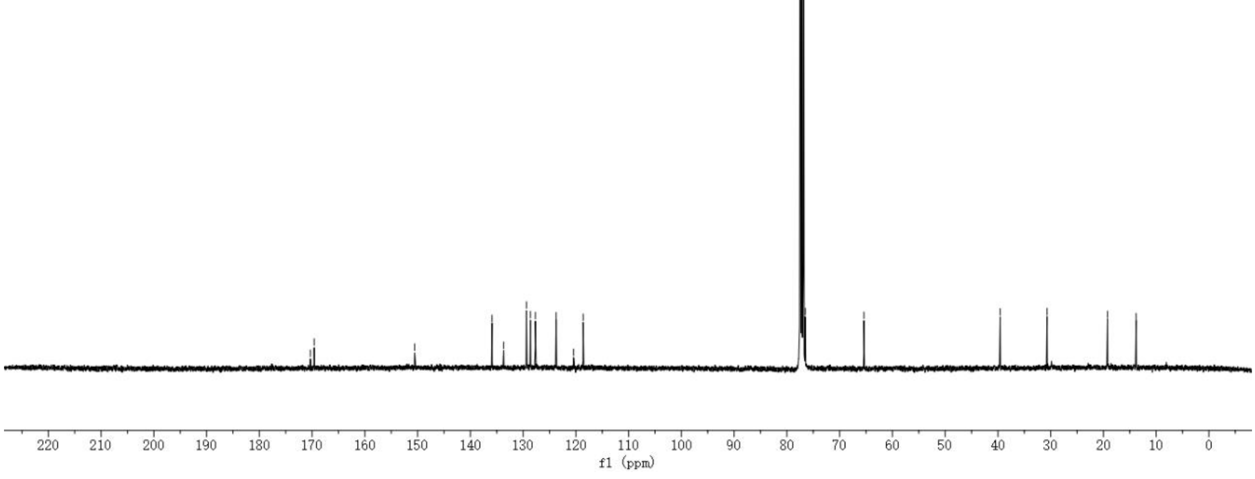

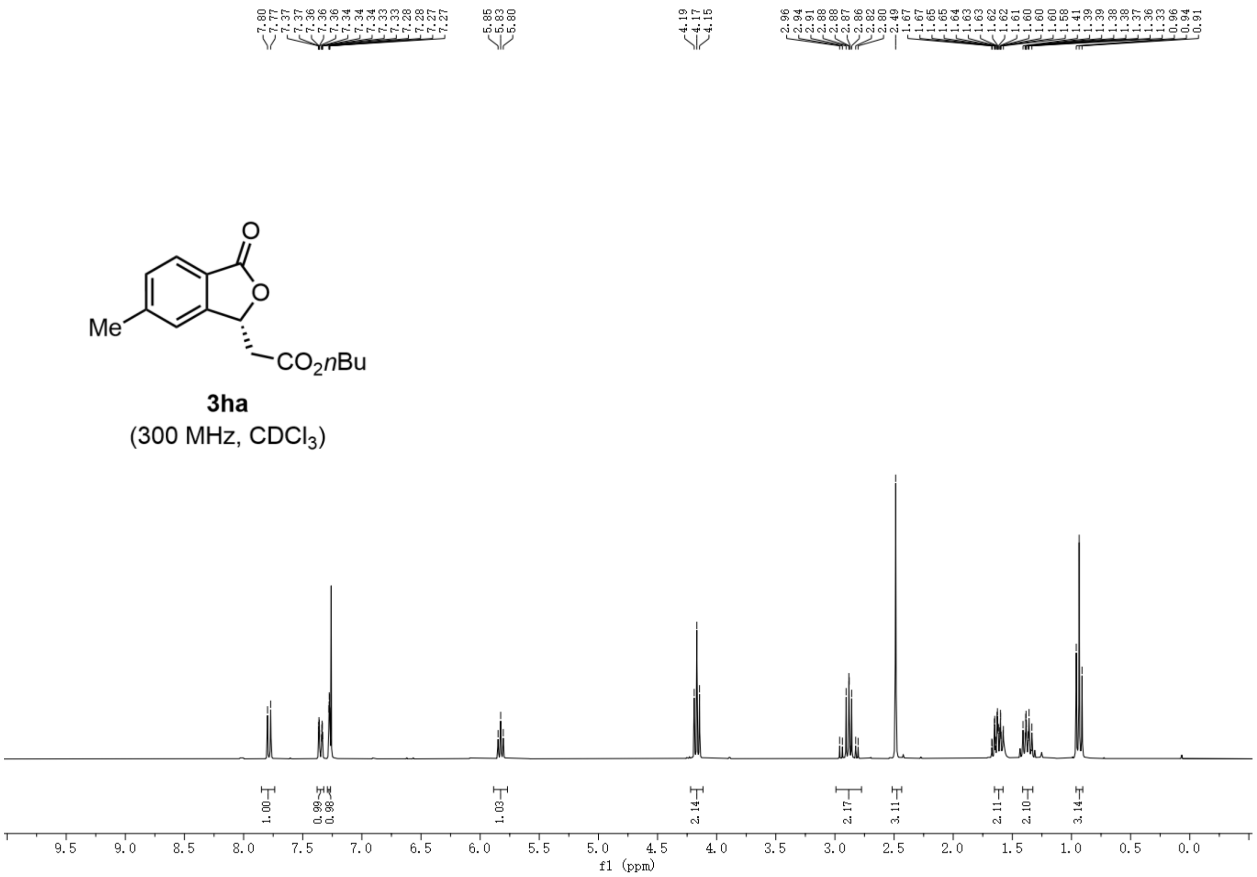

3ha
(300 MHz, $CDCl_3$)

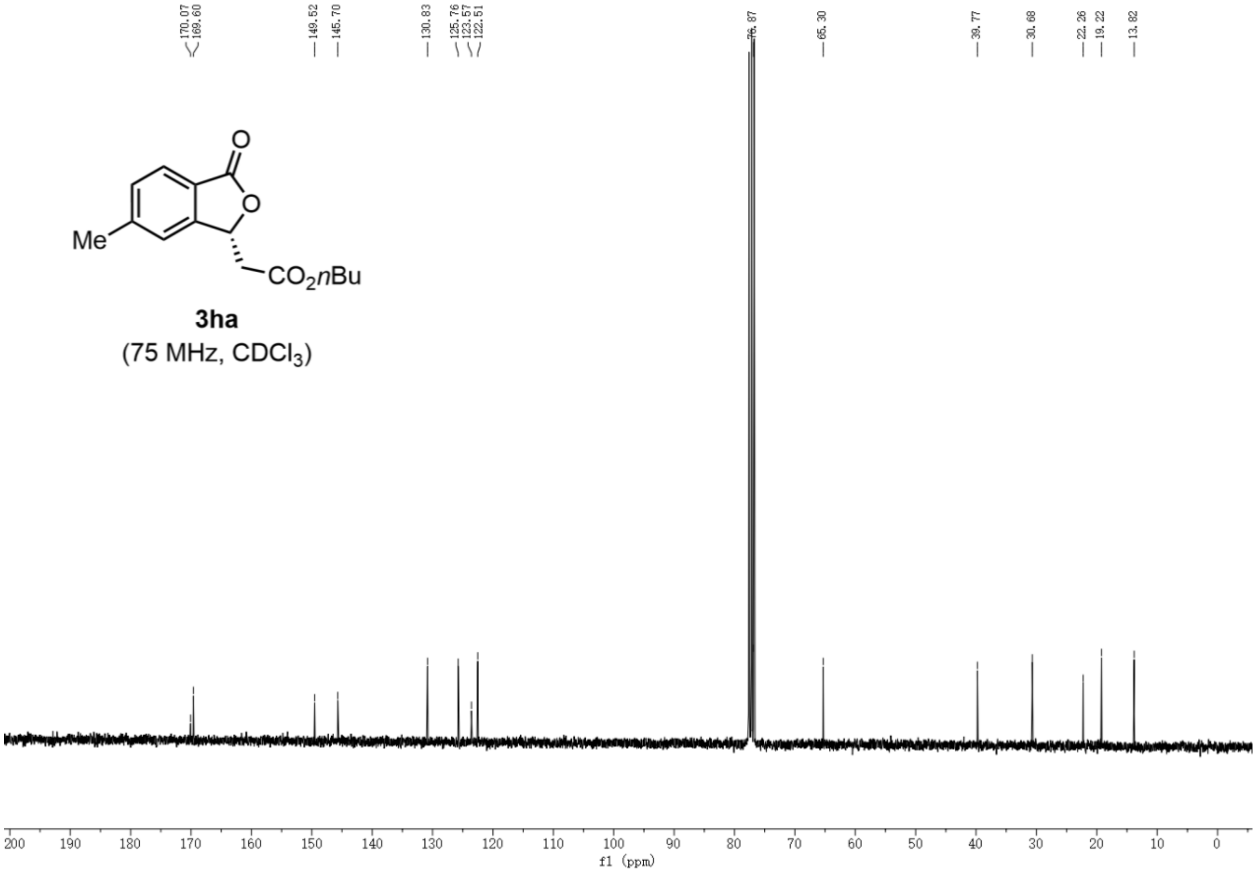

3ha
(75 MHz, $CDCl_3$)

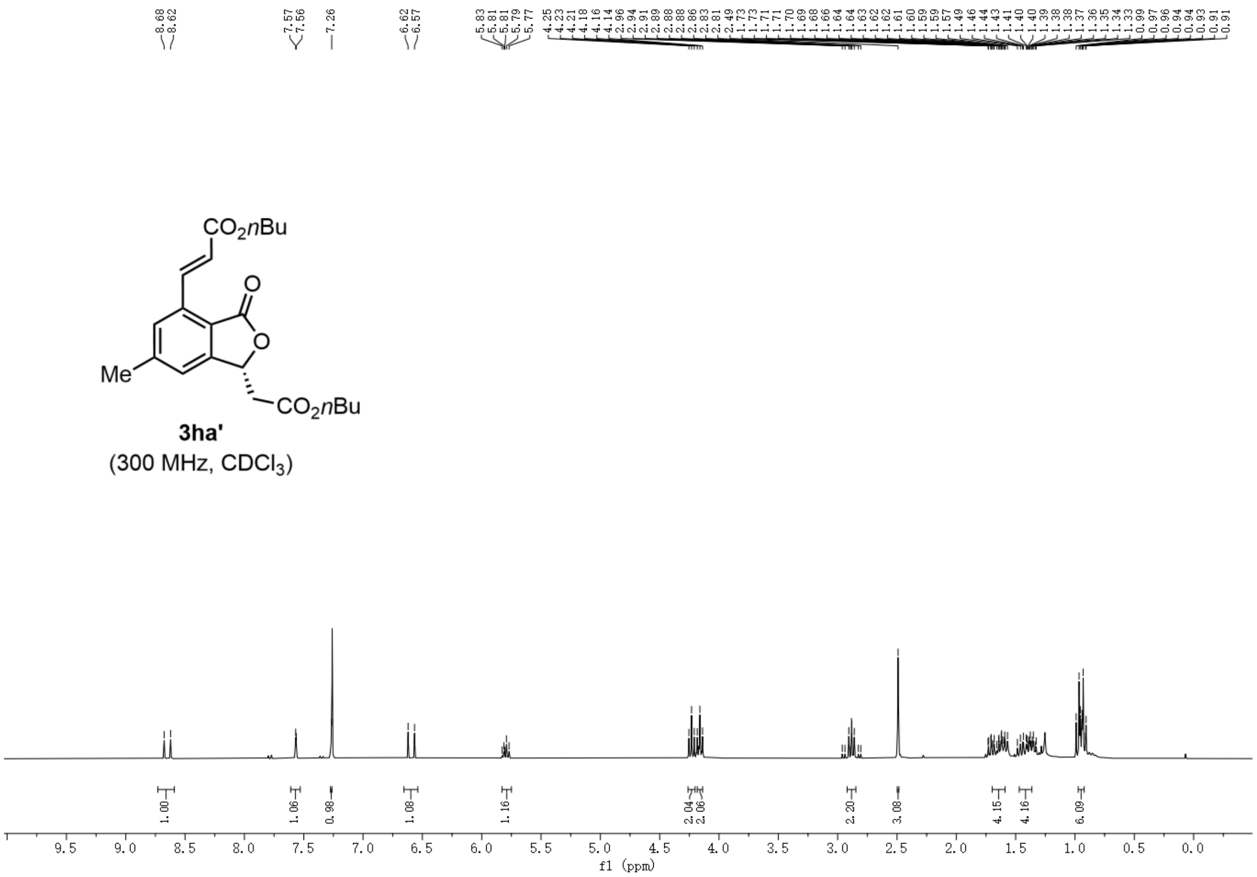

3ha'
(300 MHz, $CDCl_3$)

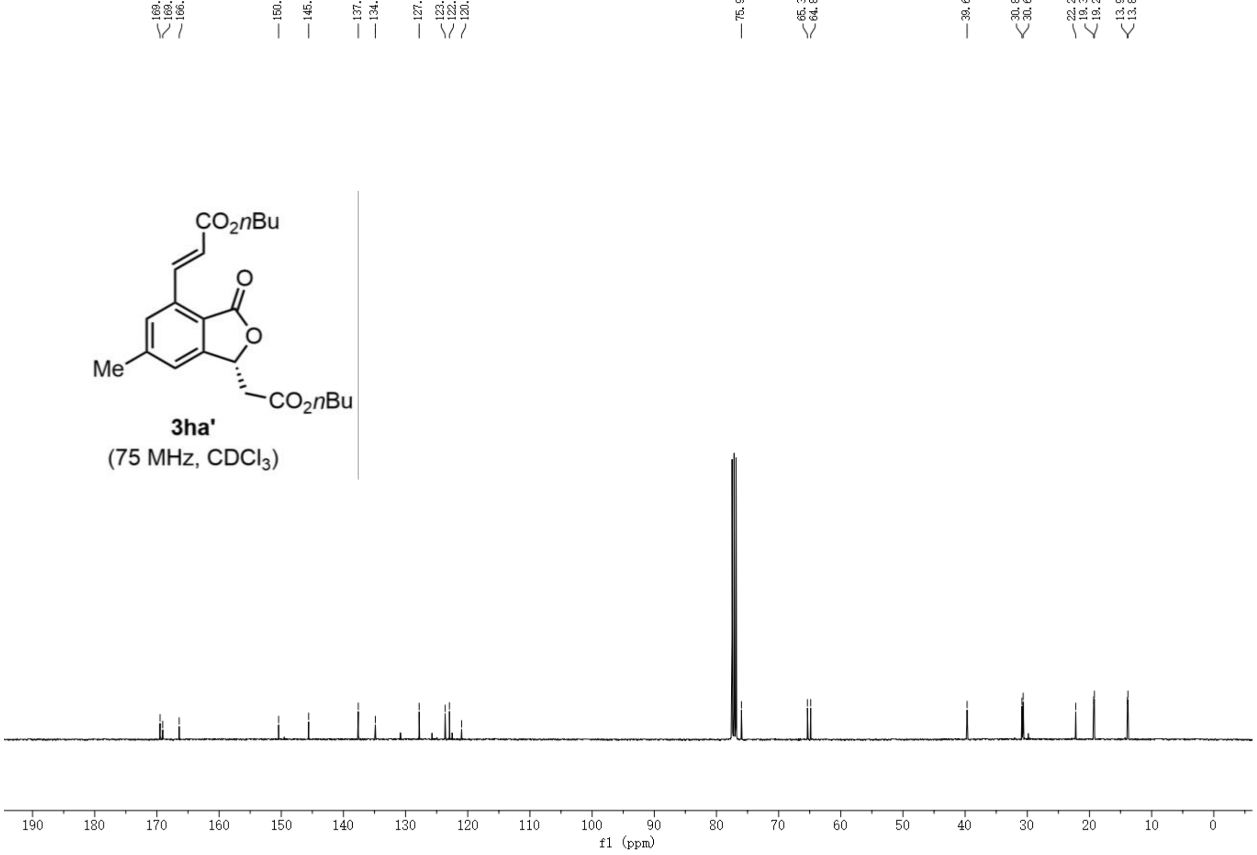

3ha'
(75 MHz, $CDCl_3$)

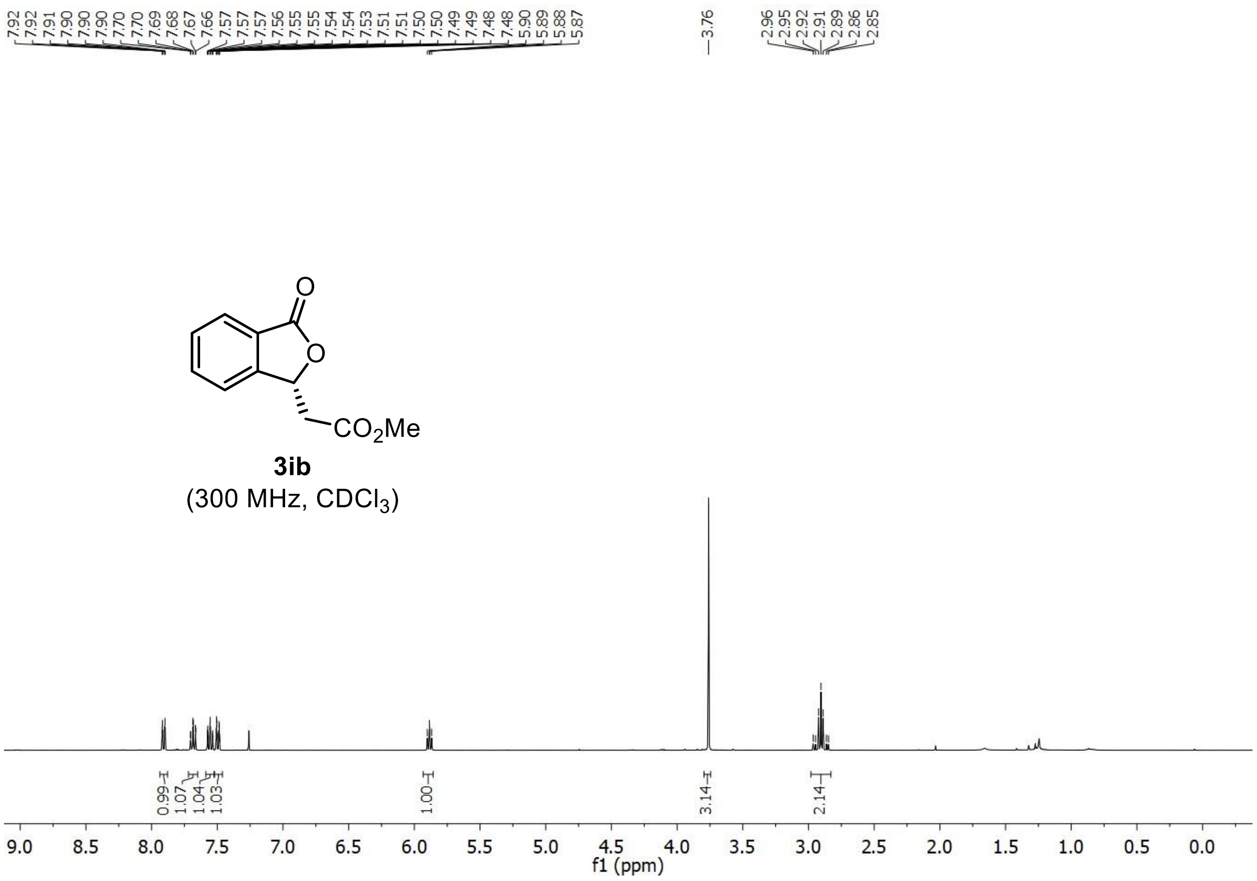
7.92 7.92 7.91 7.90 7.90 7.90 7.70 7.70 7.69 7.68 7.67 7.66 7.57 7.57 7.57 7.56 7.55 7.55 7.54 7.54 7.53 7.51 7.51 7.50 7.50 7.49 7.49 7.48 7.48 5.90 5.89 5.88 5.87
3.76
2.96 2.95 2.92 2.91 2.89 2.86 2.85
O
O
CO2Me
3ib
(300 MHz, CDCl3)
0.99 1.07 1.04 1.03
1.00
3.14
2.14
9.0 8.5 8.0 7.5 7.0 6.5 6.0 5.5 5.0 4.5 4.0 3.5 3.0 2.5 2.0 1.5 1.0 0.5 0.0
f1 (ppm)

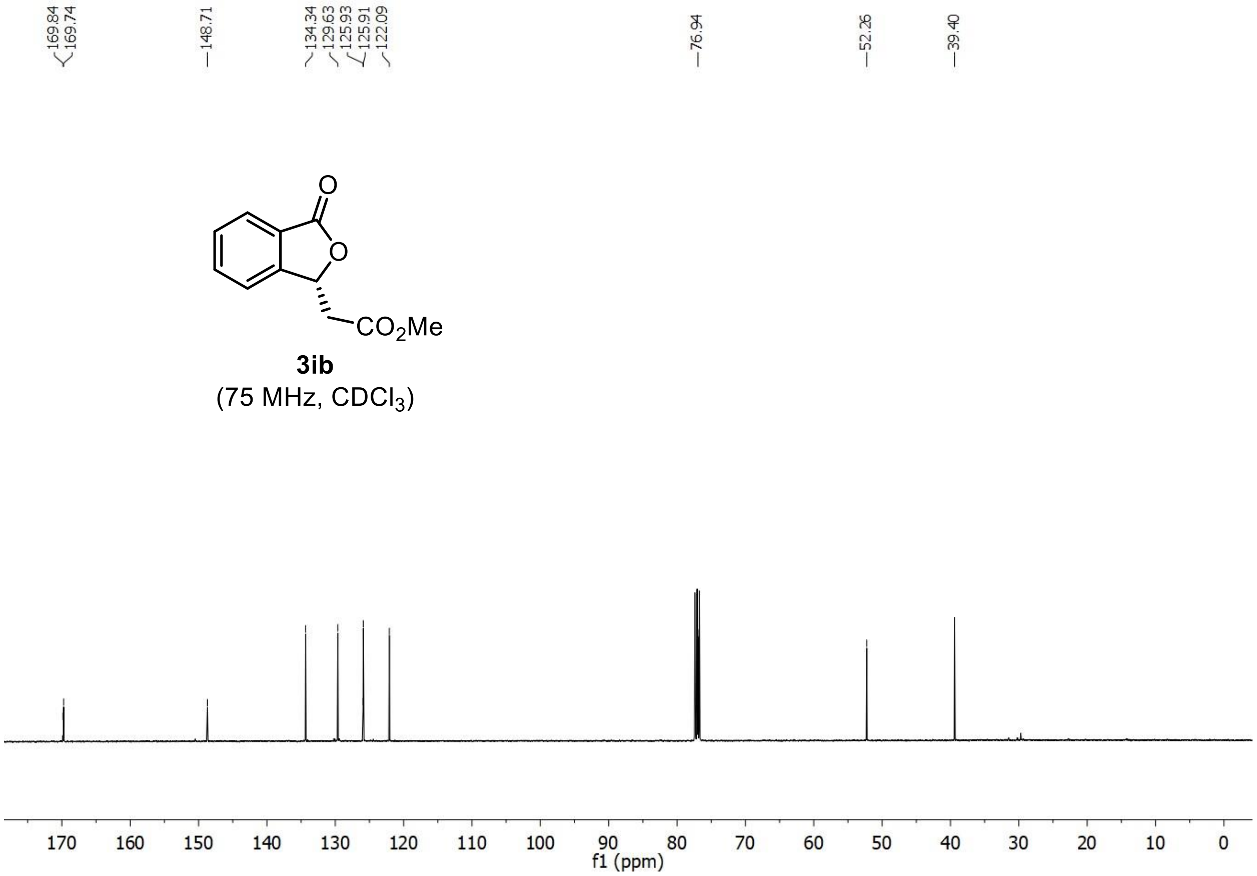
169.84 169.74
148.71
134.34 129.63 125.93 125.91 122.09
76.94
52.26
39.40
O
O
CO2Me
3ib
(75 MHz, CDCl3)
170 160 150 140 130 120 110 100 90 80 70 60 50 40 30 20 10 0
f1 (ppm)

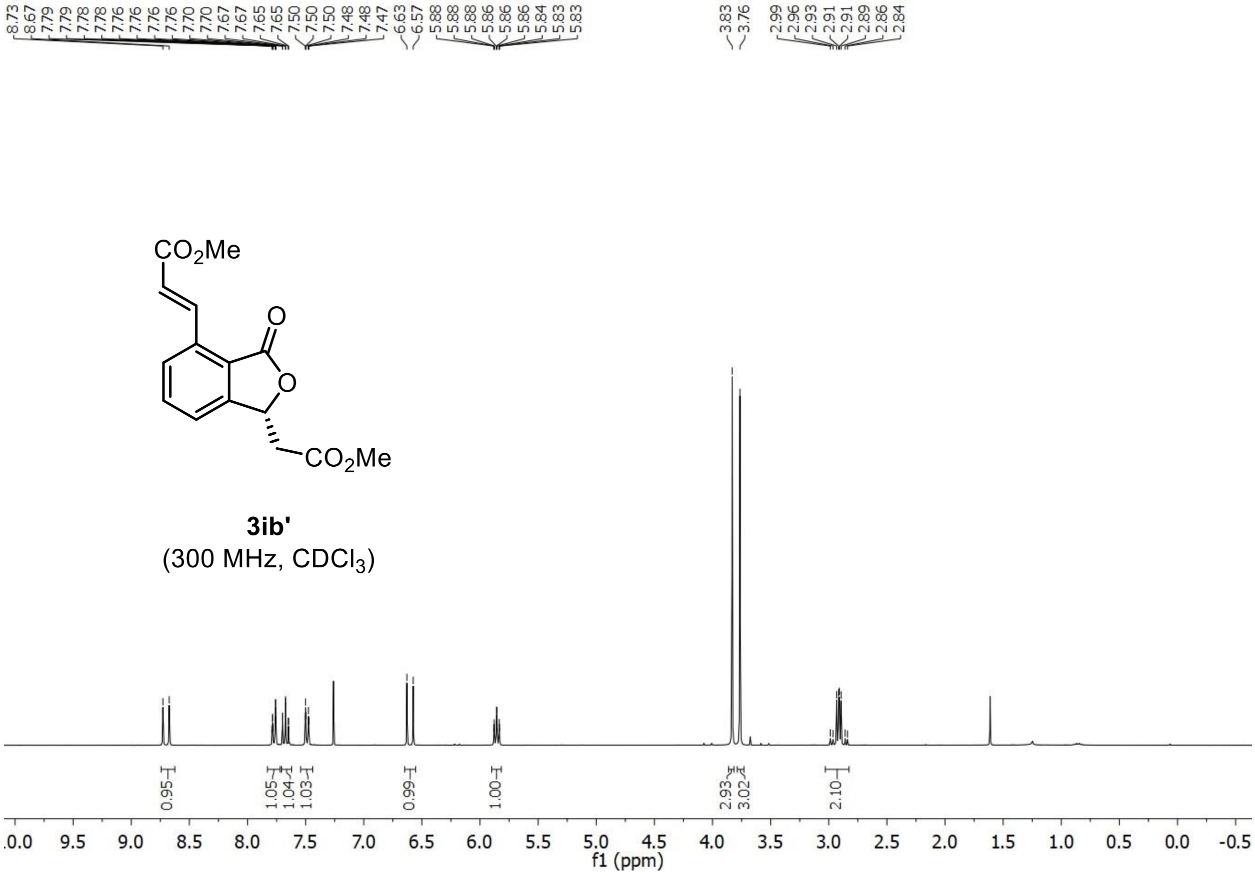
8.73 8.67 7.79 7.79 7.78 7.78 7.76 7.76 7.76 7.76 7.70 7.70 7.67 7.67 7.65 7.65 7.50 7.50 7.50 7.48 7.48 7.47 6.63 6.57 5.88 5.88 5.88 5.86 5.86 5.86 5.84 5.83 5.83
3.83 3.76
2.99 2.96 2.93 2.91 2.91 2.89 2.86 2.84
CO2Me
O
O
CO2Me
3ib'
(300 MHz, CDCl3)
0.95 1.05 1.04 1.03 0.99 1.00 2.93 3.02 2.10
f1 (ppm)

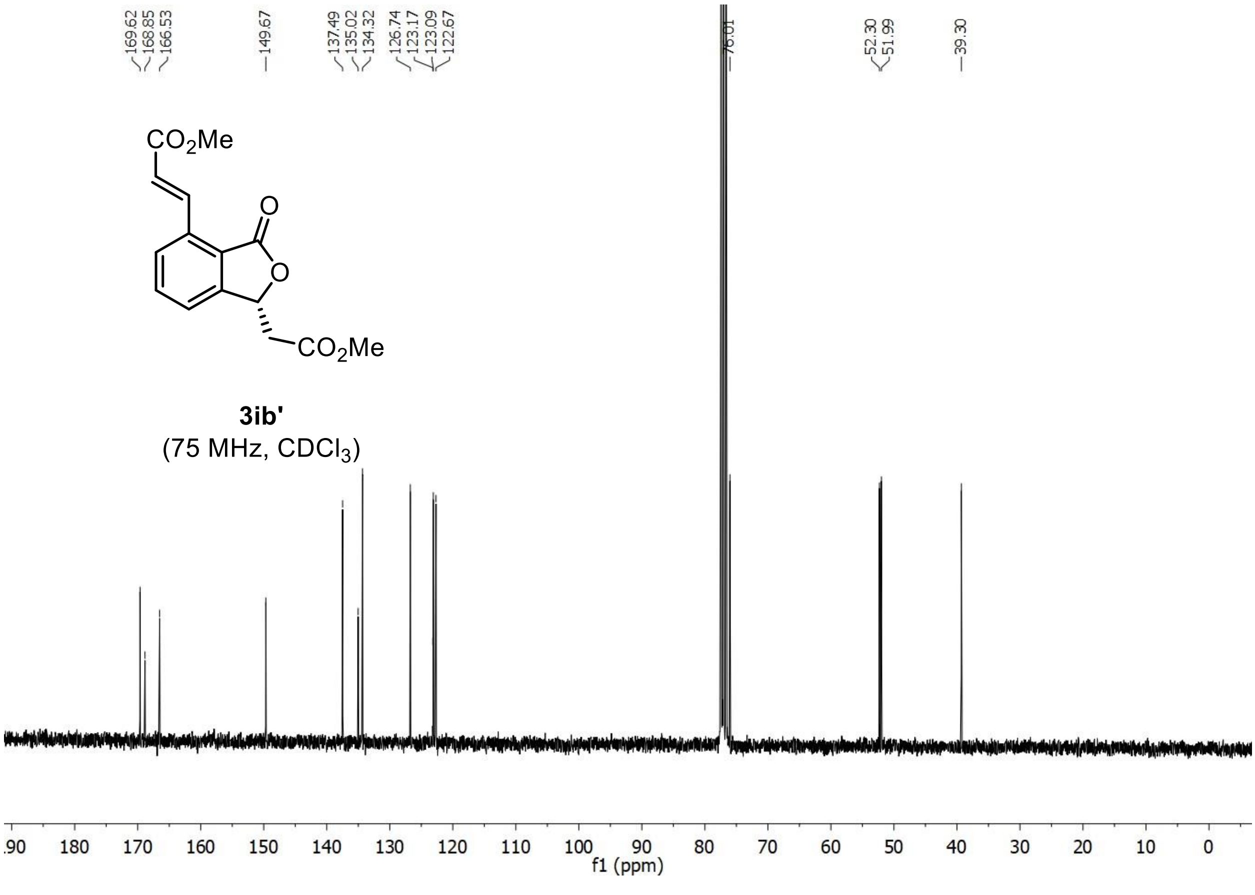
169.62 168.85 166.53
149.67
137.49 135.02 134.32 126.74 123.17 123.09 122.67
76.01
52.30 51.99
39.30
CO2Me
O
O
CO2Me
3ib'
(75 MHz, CDCl3)
f1 (ppm)
